# Potential Anticancer Agents against Melanoma Cells Based on an As-Synthesized Thiosemicarbazide Derivative

**DOI:** 10.3390/biom12020151

**Published:** 2022-01-18

**Authors:** Paweł Kozyra, Agnieszka Korga-Plewko, Zbigniew Karczmarzyk, Anna Hawrył, Waldemar Wysocki, Michał Człapski, Magdalena Iwan, Marta Ostrowska-Leśko, Emilia Fornal, Monika Pitucha

**Affiliations:** 1 Independent Radiopharmacy Unit, Faculty of Pharmacy, Medical University of Lublin, PL-20093 Lublin, Poland; pawekoz@interia.pl; 2 Independent Medical Biology Unit, Faculty of Pharmacy, Medical University of Lublin, PL-20093 Lublin, Poland; agnieszkakorga@umlub.pl; 3 Faculty of Science, Siedlce University of Natural Sciences and Humanities, PL-08110 Siedlce, Poland; kar@uph.edu.pl (Z.K.); waldemar.wysocki@uph.edu.pl (W.W.); michal-czlapski@wp.pl (M.C.); 4 Department of Inorganic Chemistry, Faculty of Pharmacy, Medical University of Lublin, PL-20093 Lublin, Poland; anna.hawryl@umlub.pl; 5 Department of Toxicology, Faculty of Pharmacy, Medical University of Lublin, PL-20093 Lublin, Poland; magda.iwan@umlub.pl (M.I.); marta.ostrowska-lesko@umlub.pl (M.O.-L.); 6 Department of Bioanalytics, Medical University of Lublin, PL-20090 Lublin, Poland; emilia.fornal@umlub.pl

**Keywords:** synthesis, thiosemicarbazide, X-ray investigation, melanoma, anticancer activity

## Abstract

In this paper, thiosemicarbazide derivatives were synthesized as potential anticancer agents. X-ray investigations for 1-(2,4-dichlorophenoxy)acetyl-4-(2-fluorophenyl) thiosemicarbazide, 1-(2,4-dichlorophenoxy)acetyl-4-(4-metylothiophenyl)thiosemicarbazide and 1-(2,4-di chlorophenoxy)acetyl-4-(4-iodophenyl)thiosemicarbazide were carried out in order to confirm the synthesis pathways, identify their tautomeric forms, analyze the conformational preferences of molecules, and identify intra- and intermolecular interactions in the crystalline state. TLC and RP-HPLC analyses were used to determine lipophilicity. The lipophilicity analysis revealed that the 4-substituted halogen derivatives of thiosemicarbazides showed greater lipophilicity compared with 2-substituted derivatives. The optimal range of lipophilicity for biologically active compounds logkw is between 4.14 and 4.78. However, as the analysis showed, it is not a decisive parameter. The cytotoxicity of the new compounds was evaluated against both the G-361 and BJ cell lines. Cytotoxicity analyses and cell-cycle and cell apoptosis assays were performed. The MTT test demonstrated that three compounds were cytotoxic to melanoma cells and not toxic to normal fibroblasts in the concentration range used. The cell cycle analysis showed that the compounds had no significant effect on the cell cycle inhibition. An extensive gene expression analysis additionally revealed that all compounds tested downregulated the expression of dihydroorotate dehydrogenase (DHODH). DHODH is a mitochondrial enzyme involved in the de novo synthesis of pyrimidines. Due to the rapid rate of cancer cell proliferation and the increased demand for nucleotide synthesis, it has become a potential therapeutic target.

## 1. Introduction

Cancer is one of the main causes of death worldwide [1,2]. The American Cancer Society estimates that, in 2021, in the United States alone, there were almost 1.9 million new cancer cases, of which almost one-third were fatal [3]. Antineoplastic drugs can be divided into inhibitors of mitotic pathways and/or DNA replication and inhibitors of molecular targets involved in tumor progression. The ability of some compounds to chelate metal ions has now been recognized as a major factor in their antiproliferative effects. For example, the complexation of thiosemicarbazones with Fe ions turned out to be of key importance for anti-tumor activity, causing oxidative damage and inhibiting ribonucleotide reductase [4]. Several anti-cancer mechanisms of action have been proposed for the new connections; for example, the destruction of the tyrosyl radical, inhibition of synthesized DNA, inhibition of Topo I/II α, and mobilization of intracellular Fe [5]. Additionally, complexes of thiosemicarbazones with Cu (II) ions turned out to be more effective against prostate cancer than the ligands themselves [6]. A novel approach to cancer treatment is targeted therapy, which focuses on inhibiting a specific molecular target. Its effectiveness is based on targeted cancer treatment, which minimizes the side effects over conventional chemotherapy. Drugs belonging to this group act as inhibitors of proliferation, inducers of apoptosis or autophagy, and as transporters of substances toxic to cancer cells [7].

Despite the advances in medicine, an effective and safe cure for cancer has still not been found. Most of the drugs currently available are chemotherapeutic agents with high cytotoxic activity but numerous side effects. The lack of selectivity of action is still a major problem in the treatment of neoplasms. Therefore, there is a need to search for new therapeutic agents.

In our research, we focus on the synthesis of new potential biological agents with a specific molecular target [8,9,10,11]. These compounds contain a thiosemicarbazide scaffold that has a documented antibacterial, antifungal, antioxidant [12,13,14], and anticancer activity [11,15,16].

In our previous studies, we have demonstrated the antiproliferative activity of thiosemicarbazide derivatives against human breast adenocarcinoma, human hepatocellular carcinoma cell lines and a significant inhibition of A549, HepG2 and MCF-7 cell division [10,11].

This work is a continuation of the research on the anticancer activities of 2,4-dichlorophenoxyacetic thiosemicarbazides. In previous studies, we demonstrated that these compounds exhibited cytotoxicity against a gastric cancer cell line without significant toxicity to normal cells. The tested substances generated DNA damage in the form of AP sites and the presence of DSB. Analysis of the UV spectrum indicated the possible intercalation of both compounds to DNA [17].

In this work, we extended the group of thiosemicarbazide derivatives containing the 2,4-dichlorophenoxyacetic group with new compounds. We examined their potential for melanoma which, according to the data of the National Cancer Institute (NCI), is malignant and the most common skin cancer [18,19]. The new compounds were characterized by means of spectroscopic methods and an X-ray analysis, and the lipophilicity parameters were determined. Biological studies were performed on G-361 human malignant melanoma.

## 2. Materials and Methods

All chemicals used for the synthesis were purchased from the companies Sigma-Aldrich (Sigma-Aldrich, MO, USA), AlfaAesar (Haverhill, MA, USA), POCH (Haverhill, MA, USA) and used without further purification. Melting points of obtained compounds were determined using Fisher-Johns block and presented without corrections. The ^1^H and ^l3^C NMR spectra were recorded on a Bruker Avance 600 spectrometer (Bruker BioSpin GmbH, Rheinstetten, Germany) in DMSO-d_6_. The chemical shifts are given in parts per million (ppm, δ scale), relative either to the inertial standard (TMS) or residual solvent peak. ATR-IR spectra were recorded over the range 4000–400 cm^−1^ on the Thermo Scientific Nicolet 6700 FTIR spectrophotometer. LC-QTOF HRMS measurements were performed using 1290 HPLC coupled with 6550 ifunnel QTOF LC/MS (Agilent Technologies, Santa Clara, CA, USA) equipped with a JetStream electrospray ion source.

### 2.1. General Procedure for the Synthesis of Thiosemicarbazide Derivatives (PK1-PK13)

Concentrations of 0.010 mole of 2,4-dichlorophenoxyacetic acid hydrazide (1), 0.010 mole of isothiocyanate and 15 mL methyl alcohol were heated at reflux temperature for 1–2 h. The desired compounds precipitated upon cooling the solution. The filtered and dried substances were crystallized from ethyl alcohol.

1-(2,4-dichlorophenoxy)acetyl-4-(2-fluorophenyl)thiosemicarbazide (PK1).

Summary formula: C_15_H_12_N_3_O_2_SCl_2_F (388.25), yield 76%, m.p. 171-172 °C. ^1^H NMR (DMSO-d_6_) δ: 4.79 (s, 2H, CH_2_); 7.22–7.60 (m, 7H, CHarom); 9.56 (s, 1H, NH); 10.07 (s, 1H, NH); 10.34 (s, 1H, NH). ^13^C NMR (DMSO-d_6_) δ: 67, 116, 122, 124, 125, 127, 128, 129, 131, 153, 166, 167, 170, 182. IR cm^−1^: 3262 (NH), 3188 (NH), 3117 (NH), 1669 (C=O). LC-QTOF HRMS (\emph{m}/\emph{z}): calculated monoisotopic mass: 388.0084, measured monoisotopic mass: 387.008.

1-(2,4-dichlorophenoxy)acetyl-4-(4-metylothiophenyl)thiosemicarbazide (PK2).

Summary formula: C_16_H_15_N_3_O_2_S_2_Cl_2_ (416.35), yield 76%, m.p. 161-162 °C. ^1^H NMR (DMSO-d_6_) δ: 2.27 (s, 3H, CH_3_); 4.92 (s, 2H, CH_2_); 7.09-7.60 (m, 7H, CHarom); 10.20 (d, 2H, 2NH); 10.24 (s, 1H, NH). ^13^C NMR (DMSO-d_6_) δ: 15, 67, 115, 122, 125, 126, 127, 128, 129, 135, 136, 153, 167, 170, 181. IR cm^−1^: 3308 (NH), 3209 (NH), 3015 (NH), 1662 (C=O). LC-QTOF HRMS (\emph{m}/\emph{z}): calculated monoisotopic mass: 416.0055, measured monoisotopic mass: 414.9978.

1-(2,4-dichlorophenoxy)acetyl-4-(4-nitrophenyl)thiosemicarbazide (PK3).

Summary formula: C_15_H_12_N_4_O_4_SCl_2_ (415.26), yield 76%, m.p. 185–186 °C. ^1^H NMR (DMSO-d_6_) δ: 4.88 (s, 2H, CH_2_); 7.15–8.25 (m, 7H, CHarom); 9.96 (s, 2H, 2NH); 10.33 (s, 1H, NH). ^13^C NMR (DMSO-d_6_) δ: 67, 115, 121, 122, 124, 125, 128, 129, 143, 145, 152, 167, 181. IR cm^−1^: 3234 (NH), 3129 (NH), 3036 (NH), 1667 (C=O). LC-QTOF HRMS (\emph{m}/\emph{z}): calculated monoisotopic mass: 415.002907, measured monoisotopic mass: 413.9954.

1-(2,4-dichlorophenoxy)acetyl-4-(3-iodophenyl)thiosemicarbazide (PK4).

Summary formula: C_15_H_12_N_3_O_2_SCl_2_I (496.16), yield 76%, m.p. 189–190 °C. ^1^H NMR (DMSO-d_6_) δ: 4.86 (s, 2H, CH_2_); 7.13–7.96 (m, 7H, CHarom); 9.78 (s, 2H, 2NH); 10.27 (s, 1H, NH). ^13^C NMR (DMSO-d_6_) δ: 67, 93, 115, 122, 125, 125, 128, 129, 130, 134, 140, 153, 167, 170, 181. IR cm^−1^: 3335 (NH), 3255 (NH), 3142 (NH), 1664 (C=O). LC-QTOF HRMS (\emph{m}/\emph{z}): calculated monoisotopic mass: 495.9145, measured monoisotopic mass: 494.9068.

1-(2,4-dichlorophenoxy)acetyl-4-(4-fluorophenyl)thiosemicarbazide (PK5).

Summary formula: C_15_H_12_N_3_O_2_SCl_2_F (388.25), yield 76%, m.p. 173–174 °C. ^1^H NMR (DMSO-d_6_) δ: 4.86 (s, 2H, CH_2_); 7.07–7.59 (m, 7H, CHarom); 9.72 (s, 2H, 2NH); 10.25 (s, 1H, NH). ^13^C NMR (DMSO-d_6_) δ: 67, 115, 115, 122, 125, 128, 129, 135, 153, 158, 16, 167, 170, 181. IR cm^−1^: 3324 (NH), 3235 (NH), 3130 (NH), 1664 (C=O). LC-QTOF HRMS (\emph{m}/\emph{z}): calculated monoisotopic mass: 388.0084, measured monoisotopic mass: 387.0002.

1-(2,4-dichlorophenoxy)acetyl-4-(2-iodophenyl)thiosemicarbazide (PK6).

Summary formula: C_15_H_12_N_3_O_2_SCl_2_F (396.16), yield 76%, m.p. 148–150 °C. ^1^H NMR (DMSO-d_6_) δ: 5.05 (s, 2H, CH_2_); 7.04–7.90 (m, 7H, CHarom); 9.60 (s, 1H, NH); 9.76 (s, 1H, NH); 10.31 (s, 1H, NH). ^13^C NMR (DMSO-d_6_) δ: 61, 100, 115, 123, 126, 128, 129, 130, 132, 136, 140, 147, 152, 168. IR cm^−1^: 3188 (NH), 3034 (NH), 2942 (NH), 1660 (C=O).

1-(2,4-dichlorophenoxy)acetyl-4-(4-iodophenyl)thiosemicarbazide (PK7).

Summary formula: C_15_H_12_N_3_O_2_SCl_2_I (496.16), yield 76%, m.p. 179–180. ^1^H NMR (DMSO-d_6_) δ: 4.86 (s, 2H, CH_2_); 7.13–7.71 (m, 7H, CHarom); 9.77 (s, 2H, 2NH); 10.26 (s, 1H, NH). ^13^C NMR (DMSO-d_6_) δ: 67, 90, 115, 122, 125, 128, 129, 137, 139, 153, 167, 170, 181. IR cm^−1^: 3296 (NH), 3212 (NH), 3133 (NH), 1666 (C=O). LC-QTOF HRMS (\emph{m}/\emph{z}): calculated monoisotopic mass: 495.9144, measured monoisotopic mass: 494.9063.

1-(2,4-dichlorophenoxy)acetyl-4-(3-bromophenyl)thiosemicarbazide (PK8).

Summary formula: C_15_H_12_N_3_O_2_SCl_2_Br (449.16), yield 76%, m.p. 183–184 °C. ^1^H NMR (DMSO-d_6_) δ: 4.86 (s, 2H, CH_2_); 7.13-7.84 (m, 7H, CHarom); 9.81 (s, 2H, 2NH); 10.28 (s, 1H, NH). ^13^C NMR (DMSO-d_6_) δ: 67, 115, 120, 122, 122, 125, 127, 128, 129, 130, 141, 153, 167, 170, 181. IR cm^−1^: 3337 (NH), 3249 (NH), 3143 (NH), 1666 (C=O). LC-QTOF HRMS (\emph{m}/\emph{z}): calculated monoisotopic mass: 447.92833, measured monoisotopic mass: 446.9204.

1-(2,4-dichlorophenoxy)acetyl-4-(1-naphtyl)thiosemicarbazide (PK9) [17].

Summary formula: C_19_H_15_N_3_O_2_SCl_2_ (420.32), yield 76%, m.p. 161–162 °C. ^1^H NMR (DMSO-d_6_) δ: 4.86 (s, 2H, CH_2_), 7.16-7.62 (m, 9H, CHaromat), 7.87-7.97 (m, 3H, CHar), 9.78 (s, 1H, NH), 9.94 (s, 1H, NH), 10.41 (s, 1H, NH). ^13^C NMR (DMSO-d_6_) δ: 67; 115; 122; 124; 125; 125; 126; 126; 127; 127; 128; 129; 131; 134; 135; 153; 167; 170; 182. IR cm^−1^: 3307 (NH), 3183 (NH), 3057 (NH), 1661 (C=O).

1-(2,4-dichlorophenoxy)acetyl-4-(3-fluorophenyl)thiosemicarbazide (PK10).

Summary formula: C_15_H_12_N_3_O_2_SCl_2_F (388.22), yield 76%, m.p. 163–164 °C. ^1^H NMR (DMSO-d_6_) δ: 4.80 (s, 2H, CH_2_); 7.02–7.61 (m, 7H, CH_arom_); 9.79 (s, 2H, 2NH); 10.26 (s, 1H, NH). ^13^C NMR (DMSO-d_6_) δ: 67, 112, 115, 121, 122, 125, 128, 130, 141, 153, 160, 163, 167, 170, 181. IR cm^−1^: 3261 (NH), 3229 (NH), 3162 (NH), 1664 (C=O). LC-QTOF HRMS (\emph{m}/\emph{z}): calculated monoisotopic mass: 388.0084, measured monoisotopic mass: 387.0002.

1-(2,4-dichlorophenoxy)acetyl-4-(2-bromophenyl)thiosemicarbazide (PK11).

Summary formula: C_15_H_12_N_3_O_2_SCl_2_Br (449.16), yield 76%, m.p. 161–162 °C. ^1^H NMR (DMSO-d_6_) δ: 4.79 (s, 2H, CH_2_); 7.11–7.68 (m, 7H, CH_arom_); 9.58 (s, 1H, NH); 9.80 (s, 1H, NH); 10.31 (s, 1H, NH). ^13^C NMR (DMSO-d_6_) δ: 66, 115, 122, 125, 128, 128, 129, 130, 131, 132, 138, 153, 167, 170, 182. IR cm^−1^: 3345 (NH), 3161 (NH), 2968 (NH), 1691 (C=O). LC-QTOF HRMS (\emph{m}/\emph{z}): calculated monoisotopic mass: 446.9211, measured monoisotopic mass: 447.9203.

1-(2,4-dichlorophenoxy)acetyl-4-(4-bromophenyl)thiosemicarbazide (PK12).

Summary formula: C_15_H_12_N_3_O_2_SCl_2_Br (449.16), yield 76%, m.p. 183–184 °C. ^1^H NMR (DMSO-d_6_) δ: 4.86 (s, 2H, CH_2_); 7.13-7.84 (m, 7H, CH_arom_); 9.80(s, 2H, 2NH); 10.28 (s, 1H, NH). ^13^C NMR (DMSO-d_6_) δ: 67, 115, 120, 125, 128, 129, 130, 141, 153, 167, 170, 181. IR cm^−1^: 3324 (NH), 3217 (NH), 3142 (NH), 1665 (C=O).

1-[[2-(2,4-Dichlorophenoxy)acetyl]amino]-3-[4-[[[2-(2,4-dichlorophenoxy)acetyl]amino]carbamoylamino]phenyl]thiourea (PK13) [17].

Summary formula: C_15_H_12_N_3_O_2_SCl_2_F (662.40), yield 76%, m.p. 158–159 °C. ^1^H NMR (DMSO-d_6_) δ: 4.80 (s, 4H, 2xCH_2_), 7.14-7.36 (m, 2H, CHaromat), 7.35–7.43 (m, 6H, CHaromat), 7.55–7.60 (m, 2H, CHaromat), 9.68–9.86 (m, 4H, 4NH), 10.26 (s, 2H, 2NH). ^13^C NMR (DMSO-d_6_) δ: 67; 115; 115; 122; 122; 125.0; 125; 126; 126; 128; 128; 129; 133; 136; 139; 152; 153; 167; 170; 182. IR cm^−1^: 3315 (NH), 3165 (NH), 1693 (C=O). LC-QTOF HRMS (\emph{m}/\emph{z}): calculated monoisotopic mass: 446.92811, measured monoisotopic mass: 446.9202.

### 2.2. X-Ray Structure Determination

X-ray data of PK1, PK2 and PK7 were collected on a KM4 CCD four-circle diffractometer: crystal sizes of 0.60 × 0.20 × 0.10 mm for PK1, 0.60 × 0.50 × 0.40 mm for PK2 and 0.50 × 0.40 × 0.40 for PK7, MoK*α* (*λ* = 0.71073 Å) radiation, *ω* scans, *T* = 296(2) K, multi-scan empirical absorption was corrected using CrysAlisPro program [20], with *T*_min_/*T*_max_ values of 0.7824/1.0000 for PK1, 0.8604/1.0000 for PK2 and 0.5259/0.7529 for PK7. The structures were solved by direct methods using SHELXS97 [21] and refined by full-matrix least-squares with SHELXL-2014/7 [21]. The N-bound H atoms were located by difference Fourier syntheses and refined freely. The remaining H atoms were positioned geometrically and treated as riding on their parent C atoms with C-H distances of 0.97 Å (CH_2_), 0.96 Å (CH_3_) and 0.93 Å (aromatic). All H atoms were refined with isotropic displacement parameters taken as 1.5 times those of the respective parent atoms. The residual density map for PK7 showed that the I24 atom is disordered over two positions designed as A and B with the occupancy factors (sof) refined to 0.54(2):0.46(2). All calculations were performed using the WINGX version 2014.1 package [22]. CCDC-2128292 for PK1, CCDC-2128293 for PK2 and CCDC-2128294 for PK7 contain the supplementary crystallographic data for this paper. These data can be obtained free of charge at www.ccdc.cam.ac.uk/conts/retrieving.html (accessed on 17 January 2022, or from the Cambridge Crystallographic Data Centre (CCDC), 12 Union Road, Cambridge CB2 1EZ, UK; fax: +44(0) 1223 336 033; email: deposit@ccdc.cam.ac.uk).

Crystal data of PK1: C_15_H_12_N_3_O_2_SCl_2_F, *M* = 388.24, triclinic, space group $P\bar{1}$, *a* = 7.3174(13), *b* = 8.2010(16), *c* = 15.629(2) Å, *α* = 82.251(14), *β* = 78.022(14), *γ* = 67.01(2)°, *V* = 843.1(3) Å^3^, *Z* = 2, *d*_calc_ = 1.529 Mg m^−3^, *F*(000) = 396, *μ*(Mo K*α*) = 0.532 mm ^− 1^, *T* = 296(2) K, 5915 measured reflections (*θ* range 2.69–28.80°), 3773 unique reflections, final *R* = 0.068, *wR* = 0.159, *S* = 1.011 for 1954 reflections with *I* > 2*σ*(*I*).

Crystal data of PK2: C_16_H_15_N_3_O_2_S_2_Cl_2_, *M* = 416.33, triclinic, space group $P\bar{1}$, *a* = 9.1146(7), *b* = 9.6738(8), *c* = 10.6620(8) Å, *α* = 85.735(6), *β* = 79.293(6), *γ* = 76.631(7)°, *V* = 898.21(13) Å^3^, *Z* = 2, *d*_calc_ = 1.539 Mg m^−3^, *F*(000) = 428, *μ*(Mo K*α*) = 0.609 mm^−1^, *T* = 296(2) K, 6597 measured reflections (*θ* range 2.16–28.85°), 4067 unique reflections, final *R* = 0.039, *wR* = 0.120, *S* = 1.026 for 3088 reflections with *I* > 2*σ*(*I*). 

Crystal data of PK7: C_15_H_12_N_3_O_2_SCl_2_I, *M* = 496.14, monoclinic, space group *C*2/*c a* = 14.7517(12), *b* = 11.2308(13), *c* = 22.628(3) Å, *β* = 103.123(10), *V* = 3651.0(7) Å^3^, *Z* = 8, *d*_calc_ = 1.805 Mg m^−3^, *F*(000) = 1936, *μ*(Mo K*α*) = 2.174 mm^−1^, *T* = 296(2) K, 8148 measured reflections (*θ* range 2.30–29.08), 4142 unique reflections, final *R* = 0.054, *wR* = 0.134, *S* = 1.058 for 3086 reflections with *I* > 2*σ*(*I*).

### 2.3. HPLC Procedure

RP-HPLC analysis was performed using a Merck-Hitachi LaChrom Elite System (Merck, Darmstadt, Germany) with diode array detector L-2455, thermostat L-2300, pump L-2130 and autosampler L-2200 with a Kinetex C18 (5µm; 150 × 4,6mm) chromatographic column (Phenomenex, Torrance, USA) as the stationary phase. The mobile phase consisted of methanol (Merck, Darmstadt, Germany) and water (double-distilled), which was degassed by use of the built-in membrane degasser. Subsequently, 5 μL methanolic solution (0.1%; *m*/*v*) of the selected samples was applied to the chromatographic column by use of a Hitachi L-2200 autosampler (LaChrom Elite, Hitachi-Merck, Darmstadt, Germany). The analysis was performed with a flow rate of 0.7 mL min^−1^ in isocratic mode using various concentrations of organic modifier (methanol) in binary polar mobile phases; percentages of methanol in water were 55–80% (%, *v*/*v*) and changed by 5% per step. Chromatograms were detected at 254 nm and the temperature of the column was 25 °C. All experiments were repeated in triplicate and the final results were taken to be the arithmetic means. Dead time was measured using uracil (Calbiochem. Merck, Darmstadt, Germany).

### 2.4. TLC Procedure

RP-TLC analysis was performed using RP-18 TLC silica gel 60 F254 plates (10 × 20 cm, Merck, Darmstadt, Germany) with a DS-II chromatographic horizontal chamber (CHROMDES, Lublin, Poland). The mobile phase consisted of methanol (POCH, Gliwice, Poland) and water with the following concentrations of methanol: 65–95% (%, *v*/*v*), with a concentration change every 5%. After drying, the spots were observed under a UV lamp with a wavelength of 254 nm.

### 2.5. Biological Assays

#### 2.5.1. Cell Culture

Biological studies were performed on human malignant melanoma G-361 (ATCC CRL-1424) cell lines and human fibroblast BJ (ATCC^®^ CRL-2522™) cell lines purchased from the American Type Culture Collection (ATCC, Manassas, VA USA). The G-361 cell lines were maintained in McCoy’s medium (Corning, NY, USA), and the fibroblast BJ cell line was cultured in EMEM (Corning, NY, USA). All media were supplemented with 10% heat-inactivated fetal bovine serum (PAN-Biotech, Aidenbach, Germany), 100 U/mL penicillin, 100 μg/mL streptomycin and 0.25 μg/mL amphotericin B (Sigma-Aldrich, St. Louis, MO, USA). Cultures were maintained at 37 °C in a humidified atmosphere of 95% air and 5% CO_2_. The experiments were performed using cells from passage 5 to 10.

#### 2.5.2. Cytotoxicity Analysis

The cytotoxicity of the new compounds was evaluated against both the G-361 and BJ cell lines. Cells were seeded into 96-well plates at a density of 3 × 10^4^ cells/well and 2 × 10^4^ cells/well. After 24 h of incubation, when 70–80% confluence was reached, the culture medium was removed and replaced with the fresh one containing the tested compounds in the concentration range of 10, 25, 50, 75, 100, 125 and 250 μM. Cells were cultured at 37 °C in the presence of 5% air with CO_2_ for another 24 h. The cytotoxicity of the test compounds was evaluated by the MTT colorimetric assay, based on the ability of live cells to convert yellow soluble tetrazolium salts (3-[4,5-dimethylthiazol-2-yl]- 2,5-diphenyltetrazolium bromide, MTT) to purple insoluble formazan by cellular dehydrogenase. After 24 h of incubation with solutions of the test compounds, 4 mg/mL MTT solution (Thermo Scientific, Waltham, MA, USA) was added to the cell cultures and incubation was continued for 4 h at 37 °C. The MTT medium was then removed, and the resulting crystals were dissolved in 200 µL of DMSO. The solution was measured for absorbance at 570 nm using a Power Wave xs spectrophotometric plate reader, BioTek Instruments (Winooski, VT, USA). The experiment was performed three times with three replicates for each concentration. IC_50_ values were determined using the AAT Bioquest IC_50_ calculator.

#### 2.5.3. Cell Cycle Assay

Cell-cycle assays was performed using NC 3000 system (Chemometec., Lillerød, Denmark) according to manufacturer’s protocol for the two-step cell cycle analysis. The G-361 cells were seeded in 12-well plates at a density of 1 × 10^5^ cells/well, and the tested compounds with concentration IC_50_ were added when 70–80% of confluence was achieved. After 24 h of incubation with tested compounds, the cells were washed with PBS and thoroughly resuspended in 250 μL lysis buffer supplemented with 10 μg/mL DAPI. After 5 min incubation at 37 °C, cells were mixed with 250 μL stabilization buffer and loaded into the 8-chamber slide NC-Slide A8 (Chemometec., Lillerød, Denmark). Cellular fluorescence was quantified using a NucleoCounter^®^ NC-3000™ image cytometer (Chemometec., Lillerød, Denmark). The obtained DNA content histograms were used to demarcate different phases of the cell cycle in tested samples. The experiments were performed three times with three replicates.

#### 2.5.4. Cell Apoptosis Assay

Cell apoptosis assays were performed using an NC 3000 system (Chemometec., Lillerød, Denmark) according to the manufacturer’s protocol for the Annexin V Assay. The G-361 cells were seeded in 6-well plates at a density of 2 × 10^5^ cells/mL and the tested compounds with concentration IC50 were added when 70–80% of confluence was achieved. After 24 h incubation, cells were suspended in 100 μL of Annexin V binding buffer with 2 µL of Annexin V-CF488A conjugate (FITC-labeled annexin V) and 2 µL of Solution 15 (Hoechst33342 stains). In the next step, the cells were incubated for 15 min at 37 °C and subsequently centrifuged at 400× *g* for 5 min. Following the removal of the supernatant, the cell pellets were resuspended in 300 µL of Annexin V binding buffer and centrifuged twice under the conditions described above. Eventually, the cell pellets were resuspended in 100 µL of Annexin V binding buffer and 2 µL of Solution 16 (propidium iodide, which stains late apoptotic and necrotic cells) was added. The prepared samples were analyzed immediately using 2-chamber NC-Slides A2™ (Chemometec., Lillerød, Denmark) and the Annexin V Assay protocol. The obtained scatterplots were used to demarcate the percentage of healthy cells and early and late apoptotic cells. The experiment was performed three times with three replicates.

#### 2.5.5. Analysis of Intracellular Thiol Levels

Analyses of the levels of cellular thiols were performed using NC 3000 system (Chemometec., Lillerød, Denmark) according to the manufacturer’s protocol for the cell vitality assay. The G-361 cells were seeded in 12-well culture plates at a density of 1 × 10^5^ cell/mL and the tested compounds in concentrations corresponding to IC_50_ value were added when 70–80% of confluence was achieved. The cells were counted, and suspended in 1 mL of culture medium. A representative sample of 190 uL was pipetted into the new Eppendorf tubes and 10 µL of Solution 5 (which contains acridine orange (AO) that stains all nucleated cells; PI which stains dead cells only; and VitaBright-48 TM (VB-28TM) which stains viable cells in an intensity-dependent manner reliant on the level of thiols) was added to the cell suspension. Next, the stained cells were loaded into 8-chamber NC-Slides A8™ (Chemometec., Lillerød, Denmark) and measured using the Vitality (VB-48) Assay protocol in the NC-3000 image cytometer. The experiment was performed three times with three replicates.

#### 2.5.6. Quantitative Real-Time PCR Analysis (qRT-PCR)

G631 cell line was seeded into 6-well plates in the concentration of 4 × 10^5^ cells/well. Thiosemicarbazide derivatives were added when 70–80% of confluence was achieved. After 48 h of incubation, 1 mL of TRIzol™ Reagent (Invitrogen, Carlsbad, CA, USA) was added to lyse the cells. Lysates were centrifuged for 5 min at 12,000× *g* at 4 °C, and clear supernatants were processed according to the manufacturer’s protocol. Then, reverse transcription was performed using NG dART RT-PCR kit (EURx, Gdańsk, Poland) and a Mastercycler gradient thermocycler (Eppendorf, Hamburg, Germany). Thermal profile of the reaction: 10 min at 25 °C, then 50 min at 50 °C, and 5 min at 85 °C. mRNA levels of the genes *DHODH, 18SRNA,* and *BACT* were assessed using TaqMan Fast Advanced Master Mix (ThermoFisher, Waltham, MA, USA). mRNA levels of the following genes: *CAT, SOD2,18SRNA TBP* were assessed using PowerUp SYBR Green Master Mix (ThermoFisher, Waltham, MA, USA) in a 7500 Fast Real-Time PCR System (ThermoFisher, Waltham, MA, USA). The primer sequences were summarized in Table 1. Thermal profile of the reactions performed: 20 s at 95 °C, followed by 40 cycles of 3 s at 95 °C and 30 s at 60 °C. The reaction was carried out in triplicates. Relative expression of the tested genes was determined by qRT-PCR and the ΔΔCt method. The statistical analysis was performed with RQ values (relative quantification, RQ = 2^−∆ΔCt^).

## 3. Results and Discussion

**Scheme 1:**
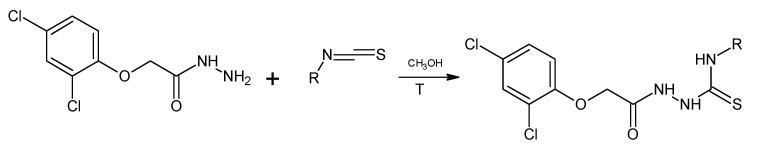
Synthesis of new thiosemicarbazide (PK1-PK12).

The title 1,4-disubstituted thiosemicarbazides (PK1–PK12) were obtained in the reaction of 2,4-dichlorophenylacetic acid hydrazide with isothiocyanates at the boiling point of methyl alcohol. The reaction proceeded in accordance with the Scheme 1.

**R** = 2-FC_6_H_4_ (PK1), 4-CH_3_SC_6_H_4_ (PK2), 4-NO_2_C_6_H_4_ (PK3), 3-IC_6_H_4_ (PK4), 4-FC_6_H_4_ (PK5), 2-IC_6_H_4_ (PK6), 4-IC_6_H_4_ (PK7), 3-BrC_6_H_4_ (PK8), C_10_H_9_ (PK9), 3-FC_6_H_4_ (PK10), 2-BrC_6_H_4_ (PK11), 4-BrC_6_H_4_ (PK12).

**Scheme 2:**
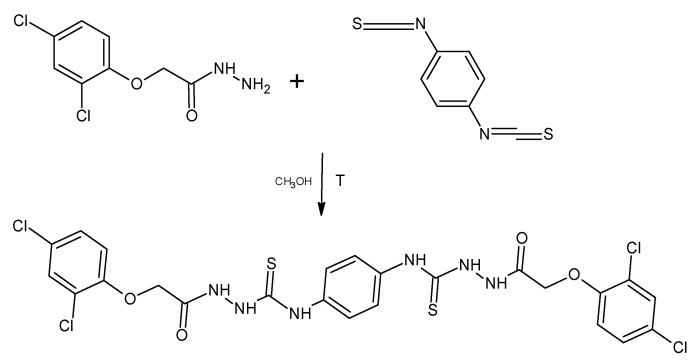
Synthesis of 1-[[2-(2,4-Dichlorophenoxy)acetyl]amino]-3- [4-[[[2-(2,4-dichlorophenoxy) acetyl]amino]carbamoylamino]phenyl]thiourea (PK13).

For the derivative PK 13, 1,4-phenylenediiothiocyanate was used for the reaction. The reactions were carried out in a methyl alcohol medium by heating the substrates at reflux temperature for 2 h (Scheme 2).

**Figure 1:**
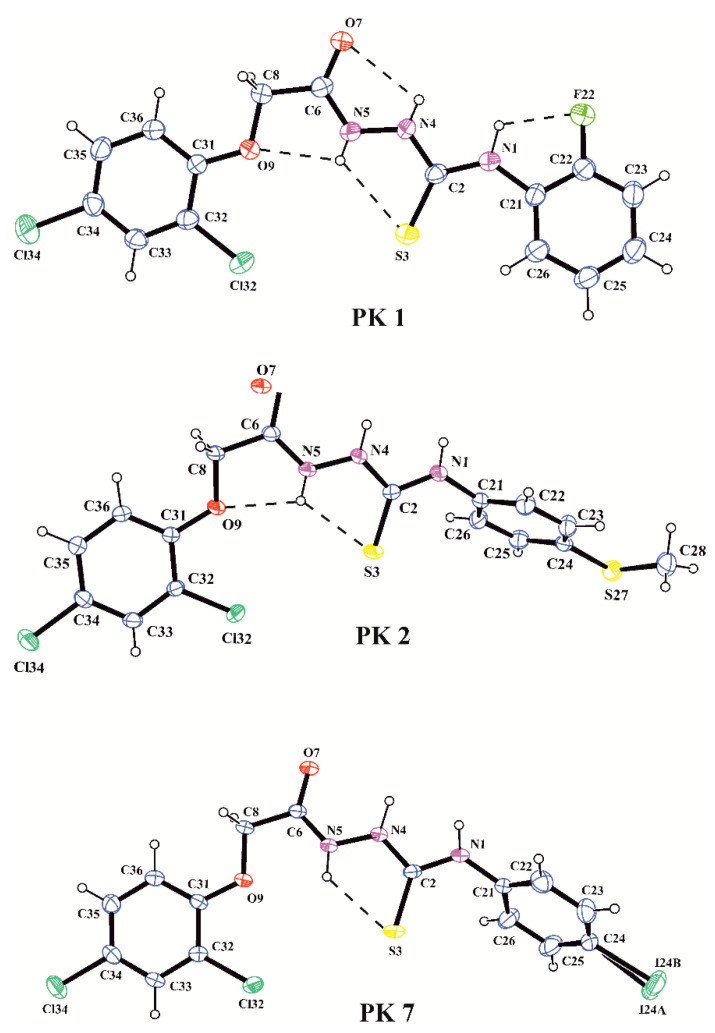
A view of the X-ray molecular structures of PK1, PK2 and PK7 with the atomic labeling scheme (probability 30%). Dashed lines indicate intramolecular hydrogen bonds.

All compounds were characterized by their melting points and ^1^H, ^13^C NMR spectra. X-ray analyses performed for PK1, PK2 and PK7 unambiguously confirmed the synthesis pathway and molecular structures observed in the crystalline state of the investigated acetylthiosemicarbazide derivatives. The molecular structures of the molecules PK1, PK2 and PK7 in the conformation observed in the crystal are shown in Figure 1. The bond distances and angles in PK1, PK2 and PK7 are in normal ranges [23] and they are very similar to those observed in our previous study on acetylthiosemicarbazide derivatives [24]. Analysis of the geometry of the investigated molecules showed that PK1, PK2 and PK7 exist in N1-amino/S3-thione/N4-amino/N5-amino/O7-keto tautomeric forms in the crystalline state. This is evidenced by the C2–S3 and C6–O7 bond lengths of 1.676(4) and 1.233(5) Å in PK1, 1.668(2) and 1.238(5) Å in PK2 and 1.674(3) and 1.234(4) Å in PK7, respectively, typical for the thione and carbonyl groups (1.681(20) Å in thioureas and 1.234(12) Å in amides; [23]) and the position of the H atom in the difference electron-density map in the vicinity of N1, N4 and N5 atoms of the thiosemicarbazide chain. The conformation of the molecules is described by the torsion angles presented in Table 2. The values of the torsion angles C21–N1–C2–N4, N1–C2–N4–N5, C2–N4–N5–C6, N4–N5–C6–C8, N5–C6–C8–09 and C6–C8–O9–C31 show that the nearly planar acetylthiosemicarbazide chain adopted the trans-trans-trans-trans-cis-trans conformation in all investigated molecules. Consequently, the torsion angles C21–N1–C2–S3 and N4–N5–C6–O7 show coplanarness of the thione and carbonyl groups with the acetylthiosemicarbazide chain. A similar position to the acetylthiosemicarbazide system is taken by the 2,4-dichlorophenyl substituent in PK1, PK2 and PK7 and the 2-fluorophenyl substituent in PK1, whereas the 4-methylthiophenyl and 4-iodophenyl substituents adopted gauche conformations in PK2 and PK7, as shown by the torsion angles C22–C21–N1–C2 and C8–O9–C31–C32, respectively. The conformations of the investigated molecules are stabilized by the intramolecular N–H…X (X = O, S, F) hydrogen bonds, as shown in Figure 1 and Table 3.

**Figure 2:**
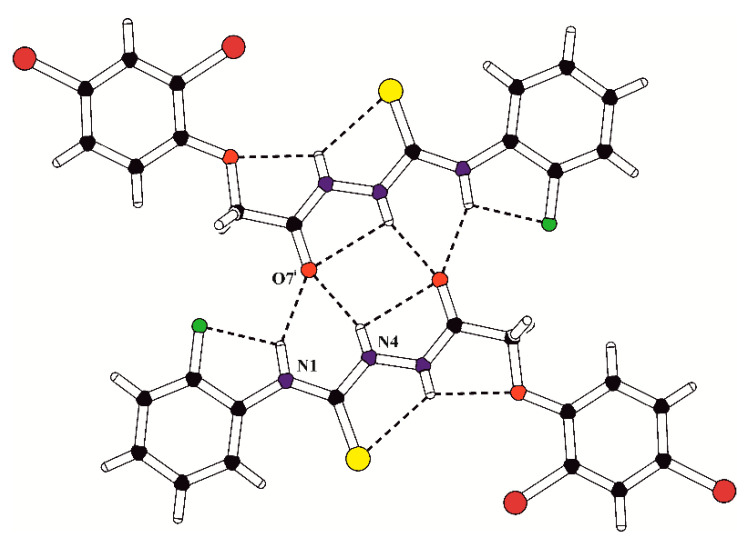
The structural motif (molecular dimer) formed by inversion-related molecules in the crystal of PK1. The intra- and intermolecular hydrogen bonds are marked with dashed lines; (*i*) = −*x*, 2 − *y*, −*z*.

In the crystal structures of PK1, PK2 and PK7, the inversion-related molecules are linked into molecular dimers via the pair of bifurcated intermolecular hydrogen bonds N4–H4…O7 and N1–H1…O7 (Table 2). The structural motif formed by intra- and intermolecular hydrogen-bound molecules in the crystal of PK1 is presented in Figure 2. Moreover, the overlapping of the pairs of parallel benzene rings belonging to inversion-related molecules in PK1 is observed with the *π*…*π* distances of 3.652(2) and 3.506(2) Å and the angle between the stacked rings of 8.3(3)°.

Notably, the intra- and intermolecular interactions occurring in the crystalline state may play a key role in the interaction of investigated thiosemicarbazides with the active site of their potential molecular target associated with the observed biological activity. Moreover, the hydrogen bonds formed by molecules in the crystals of PK1, PK2 and PK7 with the amino N–H groups as a proton donors and O-carbonyl and S-thione groups as proton acceptors are factors which stabilize the tautomeric equilibrium in the crystalline state.

Lipophilicity plays an important role in the design of new drug candidates. This parameter has implications for the ADME system: absorption, distribution, metabolism and toxicity. Lipophilicity is the affinity of a molecule or moiety for a lipophilic environment; the most frequently used parameter to quantify lipophilicity is the logP octanol-water partition coefficient. The logP parameter can be determined by reversed phase chromatographic systems (high-performance liquid chromatography, HPLC, or thin-layer chromatography, TLC) where the mechanism of substance division between the stationary and mobile phases is analogous [25,26,27,28,29]. The appropriate formulas and calculations of the logk and RM values of the tested compounds are provided in Tables S1 and S2 (Supplementary Material).

The next step of the research was to create graphs of linear relationships between the retention coefficient (logk or RM) and the concentration of the organic modifier. The values of chromatographic lipophilicity parameters and selected statistical parameters are presented in Table S3 (for HPLC and TLC). The goodness of fit values obtained for the experimental data were determined according to Jaffe [30]. By analyzing the values of the chromatographic parameters of lipophilicity (logk_W_ and R_MW_), it was discovered that for the HPLC method, the highest lipophilicity (the highest value of logk_W_) was observed for compounds PK2 (4.7804), PK9 (4.6425), PK7 (4.5363), PK12 (4.3897), PK6 (4.1439). Having analyzed the structure of the mentioned compounds, we observed that they have substituents such as: methylthiophenyl with a thiomethyl group in the para position, an iodophenyl group with the iodine atom in ortho or para positions, and a naphthalene group and bromophenyl group with a bromine atom in the para position. These substituents increase the lipophilicity of the compound by increasing the molecular weight of the compound and its branching. The compound with the lowest lipophilicity value was PK3 (2.7667), which has a nitrophenyl group in its structure, where the nitro group is in the para position.

The second part of Table S3 (Supplementary Material) shows the lipophilicity values (R_MW_) of the tested compounds obtained using the TLC method. In this method, the compound PK7 exhibited the highest lipophilicity, with an R_MW_ value = 3.8527. Other compounds with high lipophilicity, similarly to the HPLC method, were PK2 (3.2058), PK9 (3.1398) and PK12 (3.5706). The lowest values of lipophilicity were exhibited by the PK1 (2.6706) and PK3 (2.4459) samples. Moreover, the chromatographic lipophilicity parameters of the bromo-, iodo- and fluoro-thiosemicarbazide derivatives were compared (Table S3). In the case of bromo derivatives (substances PK11 and PK12) a higher value of logk_W_ was obtained for para derivative (logk_W_ = 4.3897) in comparison with ortho, where the value of logk_W_ was 3.93. Similar results were obtained for iodine derivatives (PK6 and PK7) and fluoro-derivatives (PK1 and PK5. For the ortho iodine derivative PK6, a lower value of logk_W_ (4.1439) was obtained compared with para iodine derivative (PK7), where the value of logk_W_ was 4.5363. The results for the fluorine derivatives are analogous; for the compound PK5 (para position), the value of logk_W_ was 3.8993, and for the substance PK1 (ortho position), logk_W_ was 3.4437 (Table S3). In the case of the TLC method, the values of the RM_W_ parameter for iodo-, bromo- and fluoro-derivatives were always higher for para derivatives (Table S3).

In summary, para halogen derivatives of the analyzed thiosemicarbazides demonstrated greater lipophilicity compared with ortho derivatives. The next stage of the research was the calculation of lipophilicity parameters using computational methods with the available computer programs (Table S4). The obtained lipophilicity values (milogP, cLogP, ACD/logP, ogPcons, logPChemAxon) are provided in Table S4 (Supplementary Material).

To confirm the usefulness of the applied chromatographic methods for predicting the lipophilicity of thiosemicarbazide derivatives, a correlation matrix was created based on the Pearson correlation coefficient for all experimentally determined and calculated lipophilicity parameters (Table S5). High values of Pearson’s correlation coefficient (r > 0.97) were obtained for the chromatographic parameters (logk_w_*,* R_MW_, S), which confirmed that the analyzed 1-(2,4-dichlorophenoxy)thiosemicarbazide derivatives belonged to the same congeneric compounds [31].

In addition, the experimental and calculated lipophilicity parameters were also compared using principal component analysis (PCA) and hierarchical cluster analysis (HCA). Due to the large dispersion of points, it was difficult to identify the relationship between the analyzed parameters in Figures S1 and S2 (Supplementary Materials).

Therefore, to facilitate the interpretation of the results, another chemometric tool—hierarchical cluster analysis—was used. The obtained HCA plot for the lipophilicity parameters is presented in Figure S2.

Having summarized the results of the PCA and HCA, it can be concluded that the selected chromatographic techniques are appropriate for determining the lipophilicity parameters due to the relatively high correlation between the experimental data and the obtained computational methods.

For all compounds, biological activity was assessed, using cytotoxicity analysis and cell-cycle and cell apoptosis assays. The MTT test demonstrated that compounds PK2, PK6, PK7, PK9, PK10, PK11 and PK12 were cytotoxic to melanoma cells, whereas only compounds PK2, PK6 and PK9 were not toxic to normal fibroblasts in the concentration range used (Table 4).

**Figure 3:**
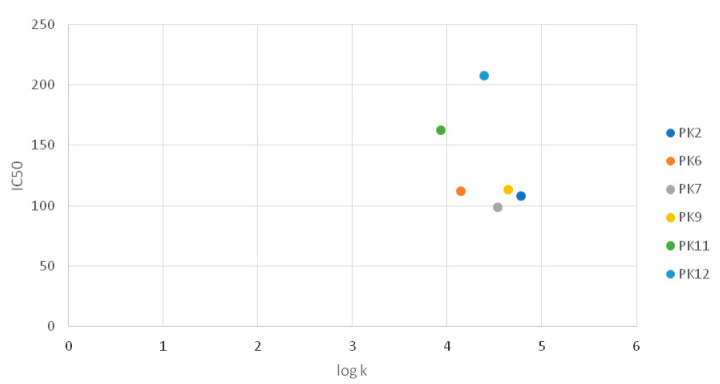
Graph comparing IC_50_ values with logk.

Taking into account the importance of lipophilicity for biological activity, analysis of the dependence of the lipophilicity logk coefficient on the IC_50_ values obtained for the tested compounds was performed based on the results of the MTT test after 24 h treatment of G-361 melanoma. The analysis was performed for compounds for which specific IC_50_ values have been determined. However, no significant correlation was found between the biological activity and lipophilicity of the compounds tested (Figure 3).

Further analysis of these particular compounds revealed that the IC_50_ values for fibroblast were 828 ± 8, 631 ± 11 and 894 ± 9 µg/mL, respectively. Therefore, compounds PK2, PK6 and PK9 were selected for further analysis.

**Figure 4:**
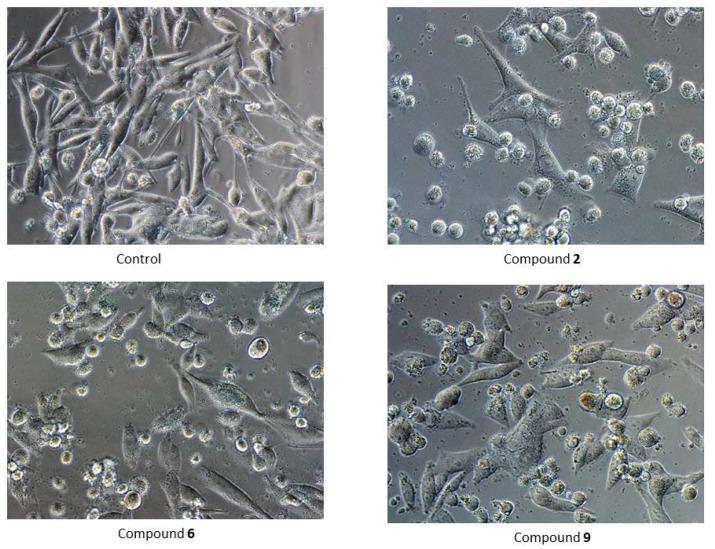
Morphology of G-361 cells after 24 h treatment with compounds PK2, PK6 and PK9 in concentrations corresponding to appropriate IC_50_ values or DMSO as a vehicle in control culture (magnification ×400).

Microscopic examination of cells treated with the tested compounds confirmed the results of the MTT assay. In each case, shrunken and detached cells were observed (Figure 4).

**Figure 5:**
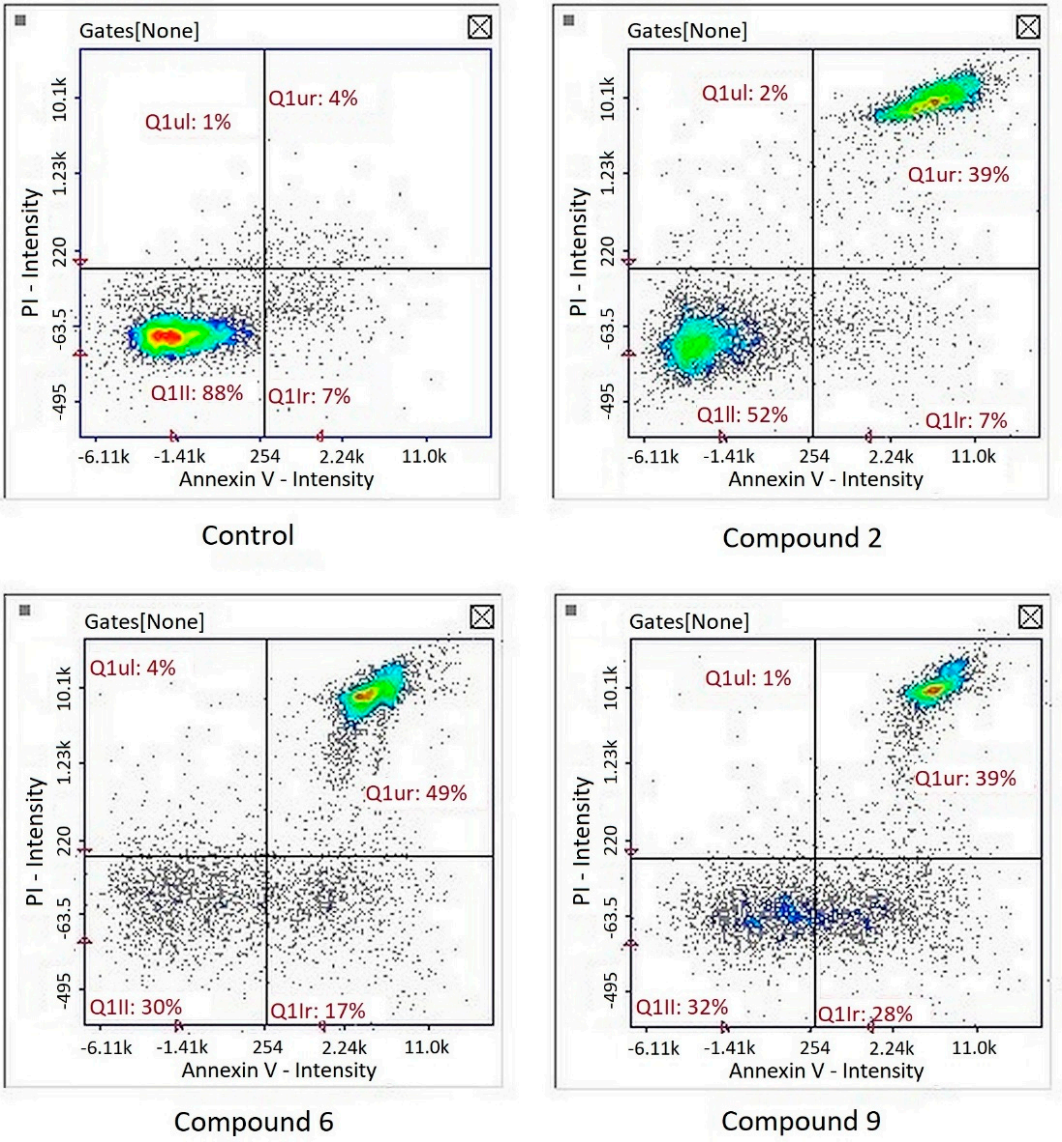
Detection of apoptotic and necrotic cells on the representative histograms in cultures of melanoma line G-361 after 24 h treatment with compounds PK2, PK6 and PK9 in concentrations corresponding to appropriate IC_50_ values or DMSO as a vehicle in control culture, using image cytometry (propidium iodide and Annexin V-FITC staining). Q1II, normal cells; Q1Ir, early apoptotic cells; Q1ur, late apoptotic cells; Q1uI, necrotic cells.

Image cytometry analysis of the cells stained with propidium iodide and Annexin V-FITC demonstrated that in the cultures treated with tested compound, the majority of the population were late apoptotic cells (Figure 5).

**Figure 6:**
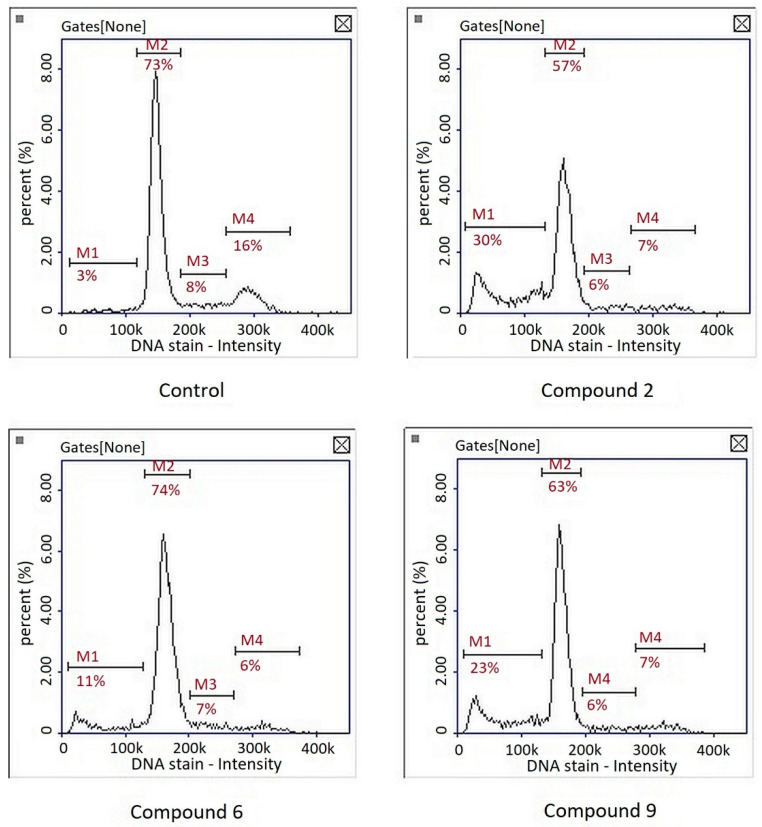
Representative histograms of cell cycle analysis in cultures of melanoma line G-361 after 24 h treatment with compounds PK2, PK6 and PK9 in concentrations corresponding to appropriate IC_50_ values or DMSO as a vehicle in control culture using image cytometry. M1, subG1 phase (apoptotic cells); M2, G1 phase; M3, S phase; M4, G2/M phase.

Cell cycle analysis showed that the compounds had no significant effect on cell cycle inhibition. A subG1 phase, corresponding to the population of apoptotic cells, was observed after treatment of the three compounds (Figure 6). Interestingly, in the studies on the gastric cancer cell line MKN74, our team showed that compound PK9 inhibited the cell cycle in S and G2 phases [9]. This was consistent with the observation that the compound had the ability to intercalate into DNA. In the case of melanoma cells, the mechanism of action of this compound seems to be different—no cell cycle arrest was observed; only a cytotoxic effect.

**Figure 7:**
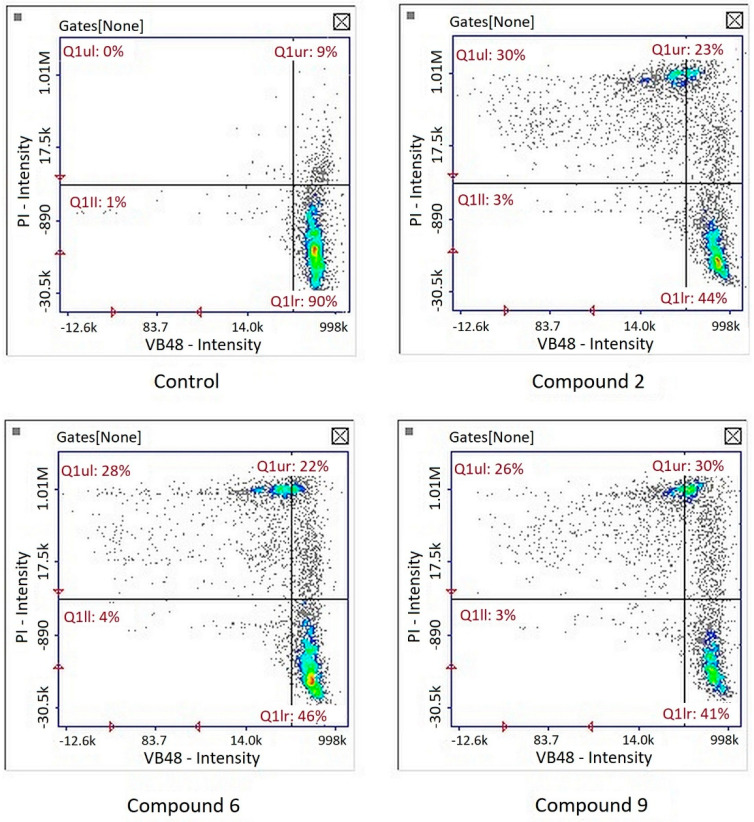
Representative histograms of reduced glutathione (GSH) content analysis in cultures of melanoma line G-361 after 24 h treatment with compounds PK2, PK6 and PK9 in concentrations corresponding to appropriate IC_50_ values or DMSO as a vehicle in control culture using image cytometry.

Reduced glutathione is one of the most important small-molecule free radical scavengers. Under oxidative stress conditions, the level of GSH in the cell decreases, which is converted to its oxidized form, GSSG [32]. Image cytometry analysis revealed decreased GSH levels in cells treated with all tested compounds, which indicates that they disrupted the redox balance (Figure 7).

Increased production of ROS has been shown to occur in melanoma cells, which has been linked to the activation of pathways associated with cell survival and tumor progression. However, this makes the redox balance in these cells less stable, and further increases in ROS generation can lead to cell death [33]. In accordance with this observation, pro-oxidants have been shown to exert cytotoxic effects against melanoma [34,35]. Therefore, concerning reduced GSH levels, the expression of enzymes related to the first line of cell defense against ROS was investigated: superoxide dismutase (*SOD2*) and catalase (*CAT*). After treatment of all three compounds, there was an increase in *SOD2* expression—an enzyme that catalyzes the dismutation of superoxide in the mitochondria into hydrogen peroxide (H_2_O_2_), which is subsequently converted to water by catalases [36]. However, *CAT* expression increased only after compound 6 treatment (Table 5). It is difficult to explain the significance of the reduction in *CAT* expression after compounds 2 and 9 treatment.

One of the enzymes that has received attention in melanoma in recent years is DHODH [37,38,39]. DHODH is a mitochondrial enzyme involved in the de novo synthesis of pyrimidines. Due to the rapid rate of cancer cell proliferation and the increased demand for nucleotide synthesis, it has become a potential therapeutic target [40]. Gene expression analysis revealed that all compounds tested downregulated the expression of dihydroorotate dehydrogenase (DHODH). Given the results of the cell cycle analysis, the inhibition of pyrimidine synthesis does not appear to be relevant to the observed cytotoxic effect. However, an association with DHODH inhibition and ROS formation has been demonstrated [41], which is consistent with these results, indicating redox imbalance. Fang et al. demonstrated that DHODH depletion partially inhibited the mitochondria complex III, decreased the mitochondrial membrane potential, and increased the generation of ROS. Research on the molecular mechanism of action will be continued.

## 4. Conclusions

The X-ray analysis performed for PK1, PK2 and PK7 confirmed the synthesis pathway and N1-amino/S3-thione/N4-amino/N5-amino/O7-keto tautomeric form in the crystalline state. The tautomeric form, nearly flat and elongated conformation of the molecules and molecular packing in the crystals were stabilized by N–H…X (X = O, S) intramolecular hydrogen bonds. Lipophilicity analysis proved that 4-substituted halogen derivatives of thiosemicarbazides showed greater lipophilicity compared with 2-substituted derivatives. The optimal range of lipophilicity for biologically active compounds logkw is between 4.14 and 4.78. However, as the analysis revealed, this is not a decisive parameter. The MTT test demonstrated that PK2, PK6 and PK9 compounds were cytotoxic to melanoma cells and not toxic to normal fibroblasts in the concentration range used. The cell cycle analysis showed that the compounds had no significant effect on cell cycle inhibition. Extensive gene expression analysis additionally revealed that all compounds tested downregulated the expression of dihydroorotate dehydrogenase (DHODH). DHODH is a mitochondrial enzyme involved in the de novo synthesis of pyrimidines. Due to the rapid rate of cancer cell proliferation and the increased demand for nucleotide synthesis, it has become a potential therapeutic target.

## Figures and Tables

**Table 1:** Primer sequences.

**Gene Symbol.**	**Gene Name**	**RefSeq**	**Assay ID**	
*DHODH*	dihydroorotate dehydrogenase (quinone)	NM_001361.4, XM_005255827.3, XM_005255829.3, XM_017022990.1	Hs00361406_m1	
*18SRNA*	Eukaryotic 18S rRNA	X03205.1	Hs99999901_s1	
*BACT*	actin beta	NM_001101.3	Hs99999903_m1	
**Gene Symbol**	**Gene Name**	**RefSeq**	**Forward**	**Reverse**
*CAT*	catalase	NM_001752	agcttagcgttcatccgtgt	tccaatcatccgtcaaaaca
*SOD2*	superoxide dismutase 2, mitochondrial	NM_000636, NM_001024465.2, NM_001024466.2, NM_001322814.1, NM_001322817.1, NM_001322819.1, NM_001322820.1	cttcagggtggtatggctgt	tggccagaccttaatgttcc
*18SRNA*	Eukaryotic 18S rRNA	X03205.1	gaaactgcgaatggctcattaaa	cacagttatccaagtgggagagg
*TBP*	TATA-box binding protein	NM_001172085.1, NM_003194.4	cggctgtttaacttcgcttc	ttcttggcaaaccagaaacc
mRNA				

**Table 2:** Selected torsion angles (°).

Torsion Angle	PK1	PK2	PK7
C22–C21–N1–C2	175.3(4)	−121.0(3)	−122.9(5)
C21–N1–C2–N4	172.2(4	179.3(2)	−175.4(4)
N1–C2–N4–N5	178.5(4)	176.2(2)	177.6(4)
C2–N4–N5–C6	−174.4(4)	−164.8(2)	−170.0(4)
N4–N5–C6–C8	176.8(4)	178.50(19)	178.5(4)
N5–C6–C8–09	0.4(5)	−10.0(3)	−8.5(5)
C6–C8–O9–C31	174.5(3)	172.41(18)	177.2(3)
C8–O9–C31–C32	−176.9(4)	170.36(18)	−177.8(4)
C21–N1–C2–S3	−8.7(7)	0.2(4)	7.5(7)
N4–N5–C6–O7	−4.1(6)	−2.0(3)	−1.6(6)

**Table 3:** Hydrogen-bond geometry (Å, °).

D–H...A	D–H	H...A	D...A	D–H...A
PK1				
N5–H5...S3	0.78(5)	2.49(5)	2.932(4)	117(4)
N5–H5...O9	0.78(5)	2.27(5)	2.563(5)	103(4)
N1–H1...F22	0.92(5)	2.15(5)	2.633(4)	111(4)
N4–H4...O7	0.79(5)	2.41(5)	2.697(5)	103(4)
N1–H1...O7*^i^*	0.92(5)	2.11(5)	2.944(5)	150(4)
N4–H4...O7*^i^*	0.79(5)	2.02(5)	2.760(5)	156(5)
(*i*) = 1 − *x*, 1 − *y*, 1 − *z*				
PK2				
N5–H5...S3	0.83(3)	2.50(2)	2.911(2)	112(2)
N5–H5...O9	0.83(3)	2.19(3)	2.552(20	107(2)
N1–H1...O7*^i^*	0.85(3)	2.19(3)	2.975(2)	154(3)
N4–H4...O7*^i^*	0.84(3)	2.12(3)	2.902(3)	155(2)
(*i*) = 1 − *x*, 1 − *y*, 1 − *z*				
PK5				
N5–H5...S3	0.86(6)	2.33(5)	2.915(4)	126(4)
N1–H1...O7*^i^*	0.85(6)	2.10(6)	2.936(4)	167(4)
N4–H4...O7*^i^*	0.98(6)	1.93(6)	2.833(4)	152(4)
(*i*) = 1 − *x*, 1 − *y*, −*z*				

**Table 4:** IC_50_ values obtained for the tested compounds based on MTT test results after 24 h treatment of G-361 melanoma and BJ normal fibroblast cell lines. The IC_50_ value was determined using the AAT Bioquest IC_50_ calculator.

Compound	IC_50_ [µM]	
	G-361	BJ
PK1	>250	>250
PK2	108 ± 6	>250 (828 ± 8)
PK3	>250	>250
PK4	>250	>250
PK5	>250	>250
PK6	112 ± 4.76	>250 (631 ± 11)
PK7	99 ± 4	216±76
PK8	>250	>250
PK9	113 ± 4	>250 (894 ± 9)
PK10	218 ± 5.34	223 ± 7
PK11	162 ± 2	217.46 ± 64.33
PK12	2078 ± 4	189 ± 7
PK13	>250	>250

**Table 5:** Relative mRNA expression level of selected genes. The results were calculated as RQ values and are presented as the mean ± SD value of three independent experiments. Significantly changed median RQ levels are marked with a color scale.

	*DHODH*			*CAT*			*SOD2*			Scale (RQ)	
Control	1.000 ± 0.016			1.016 ± 0.206			1.001 ± 0.053			>2.00	
PK2	0.463 ± 0.016			0.107 ± 0.013			1.486 ± 0.056			1.51–1.99	
PK6	0.824 ± 0.030			2.691 ± 0.127			1.249 ± 0.080			1.11–1.50	
PK9	0.375 ± 0.018			0.354 ± 0.022			1.293 ± 0.100			0.91–1.10	
										0.61–0.90	
										0.21–0.6	
										<0.21	

## Data Availability

Not applicable.

## Supplementary Materials

The following are available online at https://www.mdpi.com/article/10.3390/biom12020151/s1, Table S1: The retention time tR and logk coefficients of the tested compounds for the respective concentrations of methanol in water (*v/v*), Table S2: R*_f_* and R*_M_* parameters values of the tested compounds for the respective concentrations of methanol in water (*v/v*), Table S3: Linear equa-tion parameters for the RP-HPLC and RP-TLC systems, Table S4: logP values calculated using computational methods. Table S5: Correlation matrix of the lipophilicity parameters experimen-tally determined and calculated using different computer programs. Figure S1: The relationship of PC1 vs. PC2 for the parameters of lipophilicity (experimentally determined and calculated). Figure S2: Dendrogram of experimentally and computationally obtained lipophilicity parameters (based on the Pearson correlation coefficient).
