# Supplementary material for: Potential Anticancer Agents against Melanoma Cells Based on an As-Synthesized Thiosemicarbazide Derivative
Source: Biomolecules. 2022 Jan 18;12(2):151. doi: 10.3390/biom12020151 (PMC8961551; doi:10.3390/biom12020151)

Suplementary material

Lipophilicity analysis:

In the case of HPLC the chromatographic lipophilicity parameter logkw were obtained by the extrapolation of the retention parameter logk to pure water, according to Equation:

$$logk = logk_w - S \cdot \phi \quad (1)$$

where $logk_W$ is the value of the retention factor of a substance in pure water, S is the slope of the regression curve, and ϕ is the concentration of the organic modifier.

The values of $logk$ were calculated based on the raw HPLC data using the formula:

$$logk = \log\left(\frac{t_R - t_0}{t_0}\right) \quad (2)$$

where t_R is the retention time and t_0 is the dead retention time (determined for uracil).

Whereas, in the case of TLC the retention parameter (R_M) is calculated using the formula:

$$R_M = \log\left(\frac{1 - R_f}{R_f}\right) \quad (3)$$

where R_f is the retardation factor.

Table S1. The retention time t_R and $logk$ coefficients of the tested compounds for the respective concentrations of methanol in water (v/v)

	RP-HPLC											
name	t_R						$logk$					
	55	60	65	70	75	80	55	60	65	70	75	80
**PK1**	14.86	6.32	5.54	3.94	3.08	2.54	0.8183	0.3504	0.2685	0.0176	-0.2248	-0.5097
**PK2**	39.71	19.15	10.41	6.23	4.21	2.79	1.2847	0.9455	0.6401	0.3479	0.0724	-0.3584
**PK3**	10.72	7.48	5.47	4.2	3.41	2.87	0.6502	0.4527	0.2599	0.0705	-0.1132	-0.3193
**PK5**	16.53	9.41	5.90	4.08	3.13	2.51	0.8712	0.5827	0.3099	0.0469	-0.2064	-0.5319
**PK6**	31.20	16.51	9.47	5.29	4.19	3.21	1.1737	0.8731	0.5889	0.2408	0.0686	-0.1839
**PK7**	34.38	24.38	12.41	6.90	4.42	3.13	1.2186	1.0608	0.7321	0.4108	0.1106	-0.2123
**PK9**	36.90	21.67	9.41	5.71	3.97	3.01	1.2511	1.0049	0.5855	0.2919	0.0241	-0.2584
**PK11**	24.03	10.51	7.54	4.92	3.58	2.89	1.0515	0.6424	0.4604	0.1901	-0.0681	-0.3384
**PK12**	27.89	18.37	9.60	5.68	3.85	2.89	1.1215	0.9253	0.5964	0.2885	-0.0023	-0.3101

Table S2. R_f and R_M parameters values of the tested compounds for the respective concentrations of methanol in water (v / v)

name	RP-TPLC													
	R_f							R_M						
	65	70	75	80	85	90	95	65	70	75	80	85	90	95
**PK1**	0.19	0.28	0.35	0.45	0.52	0.60	0.70	0.6297	0.4102	0.2688	0.0872	-0.0348	-0.1761	-0.3679
**PK2**	0.15	0.23	0.32	0.44	0.53	0.64	0.71	0.7533	0.5248	0.3274	0.1047	-0.0522	-0.2499	-0.3889
**PK3**	0.24	0.25	0.38	0.48	0.55	0.61	0.68	0.5006	0.4771	0.2126	0.0348	-0.0872	-0.1943	-0.3274
**PK5**	0.21	0.32	0.39	0.51	0.59	0.71	0.73	0.5754	0.3274	0.1943	-0.0174	-0.1581	-0.3889	-0.4319
**PK6**	0.18	0.27	0.28	0.45	0.55	0.65	0.65	0.6585	0.4319	0.4102	0.0872	-0.0872	-0.2688	-0.2689
**PK7**	0.10	0.15	0.22	0.37	0.47	0.61	0.68	0.9542	0.7533	0.5497	0.2311	0.0522	-0.1943	-0.3274
**PK9**	0.18	0.27	0.33	0.47	0.57	0.70	0.74	0.6585	0.4319	0.3076	0.0522	-0.1224	-0.3679	-0.4543
**PK11**	0.19	0.28	0.35	0.48	0.57	0.59	0.73	0.6297	0.4102	0.2688	0.0348	-0.1224	-0.1581	-0.4319
**PK12**	0.11	0.20	0.28	0.40	0.52	0.63	0.69	0.9079	0.6021	0.4102	0.1761	-0.0348	-0.2311	-0.3475

Table S3. Linear equation parameters for the RP-HPLC and RP-TLC systems.

name	RP-HPLC						RP-TLC					
	$logk_W$	$-S$	r	n	F	SD	R_{MW}	$-S$	r	n	F	SD
**PK1**	3.4437	4.9238	0.9871	6	151.45	0.084	2.6706	3.1923	0.9975	7	1001.77	0.0270
**PK2**	4.7804	6.3581	0.9979	6	983.51	0.042	3.2058	3.8253	0.9977	7	1094.25	0.0306
**PK3**	2.7667	3.8522	0.9999	6	28991.50	0.005	2.4459	2.9474	0.9881	7	205.81	0.0544
**PK5**	3.8993	5.5119	0.9995	6	3666.90	0.019	2.7612	3.4335	0.9926	7	332.22	0.0498
**PK6**	4.1439	5.4574	0.9963	6	537.65	0.049	2.8125	3.3436	0.9795	7	117.92	0.0815
**PK7**	4.5363	5.9005	0.9963	6	542.17	0.053	3.8527	4.4553	0.9966	7	734.09	0.0435
**PK9**	4.6425	6.1619	0.9975	6	787.41	0.046	3.1398	3.8345	0.9959	7	599.05	0.0414
**PK11**	3.9300	5.3438	0.9958	6	478.75	0.051	2.7832	3.3663	0.9908	7	269.45	0.0542
**PK12**	4.3897	5.8565	0.9983	6	1184.60	0.035	3.5706	4.1984	0.9945	7	455.64	0.0520

Table S4. logP values calculated using computational methods.

name	*milogP*	*cLogP*	*ACD/logP*	$logP_{cons}$	$logP_{ChemAxon}$
**PK1**	3.51	2.9	3.76+/- 0.67	4.05	4.13
**PK2**	3.85	3.27	4.19+/- 0.66	4.53	4.65
**PK3**	3.35	1.87	4.26+/- 0.65	2.18	1.51
**PK5**	3.56	2.9	4.25+/- 0.67	2.61	1.70
**PK6**	4.43	3.23	4.74+/- 0.67	3.55	2.69
**PK7**	4.48	3.23	5.23+/- 0.67	3.40	2.55
**PK9**	4.55	3.99	5.03+/- 0.64	4.89	5.00
**PK11**	4.16	3.52	4.48+/- 0.67	4.67	4.79
**PK12**	4.20	3.52	4.97+/- 0.67	4.67	4.79

Table S5. Correlation matrix of the lipophilicity parameters experimentally determined and calculated using different computer programs.

	$logk_w$	***$-S_{HPLC}$***	***R_{MW}***	***$-S_{TLC}$***	***milogP***	***cLogP***	***ACD/logP***	***Log Pcons***	***logP ChemAxon***
$logk_w$	1								
$-S_{HPLC}$	**0.985827**	1							
R_{MW}	**0.734769**	0.691854	1						
$-S_{TLC}$	**0.753878**	**0.725243**	**0.990428**	1					
milogP	**0.74034**	0.65753	0.634226	0.627778	1				
cLogP	**0.827746**	**0.841247**	0.559446	0.606069	**0.793463**	1			
ACD/logP	0.574755	0.467179	0.667558	0.645095	**0.848456**	0.499167	1		
logPcons	0.646951	0.652426	0.329624	0.328501	0.537054	**0.799155**	0.260655	1	
logPChemAxon	0.539421	0.560854	0.275706	0.286048	0.401846	**0.738152**	0.116878	**0.974213**	1

High values Pearson correlation coefficient (r > 0.97) were obtained for the chromatographic parameters (*$logk_w$*, R_{MW}, S), which confirm that the analyzed 1-(2,4-dichlorophenoxy) thiosemicarbazide derivatives belong to the same congeneric compounds [31]. Relatively high values of the correlation coefficient (r> 0.82) were obtained for the following relationships: milogP and ACD/logP (r = 0.8485), clogP and S_HPLC (r = 0.8413), clogP and *$logk_w$* (r = 0.8278). The value of r > 0.72 for the following correlations: *$logk_w$* and R_MW, *$logk_w$* and S_{TLC}, *$logk_w$* and milogP, S_{HPLC} and S_TLC, clogP and milogP, clogP and logPcons, clogP and logPChemAxon. The high value of Pearson correlation coefficient for the experimental and calculated lipophilicity parameters confirms the usefulness of the applied chromatographic methods (Table 5S).

The highest value of Pearson correlation coefficient (r > 0.90) proves the highest correlation between the objects. In this case (Figure 2S), it can be concluded that the strongest correlation was obtained for clusters including the following parameter pairs: *$logk_w$* and S_{HPLC}, R_MW and S_{TLC}, logcons, logPChemAxon. A good correlation (r = 0.85) was also obtained for the following groups: milogP and ACDlogP; clogP and *$logk_w$* and S_{HPLC}.

Figure S1. The relationship of PC1 vs. PC2 for the parameters of lipophilicity (experimentally determined and calculated).

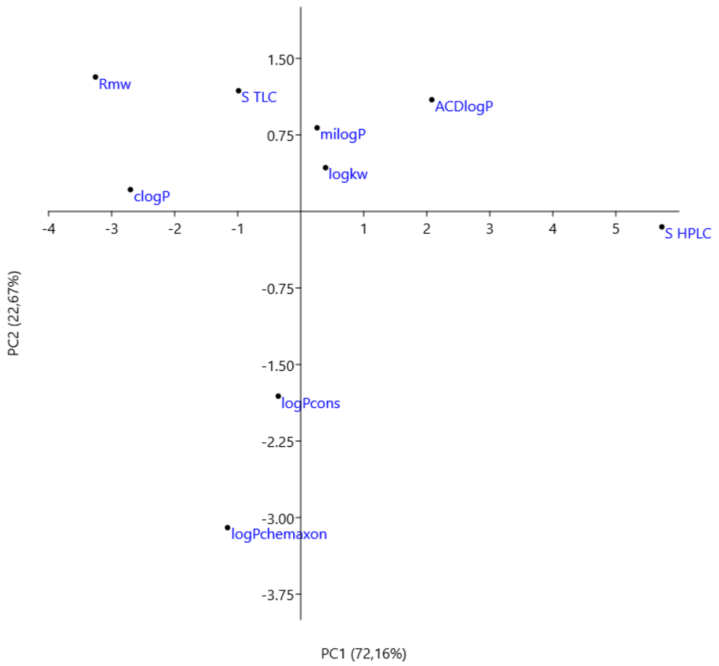

Figure S2. Dendrogram of experimentally and computationally obtained lipophilicity parameters (based on the Pearson correlation coefficient).

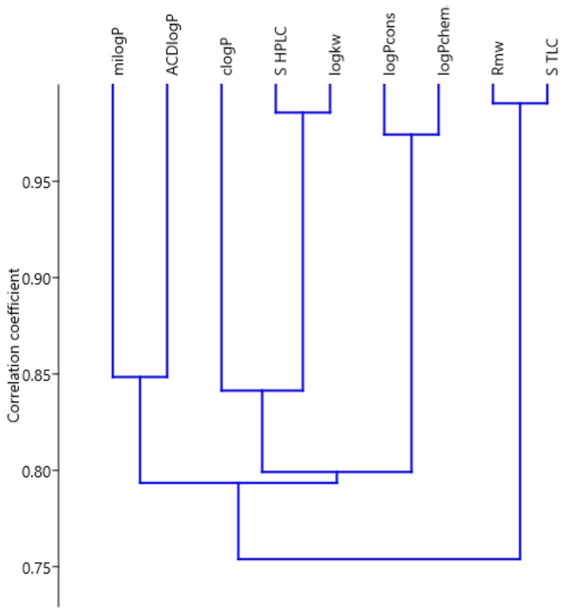

X-ray analysis

PK1

```
data_shelx

_audit_creation_method           'SHELXL-2014/7'
_shelx_SHELXL_version_number      '2014/7'
_chemical_name_systematic         ?
_chemical_name_common             ?
_chemical_melting_point           ?
_chemical_formula_moiety          'C15 H12 Cl2 F N3 O2 S'
_chemical_formula_sum             'C15 H12 Cl2 F N3 O2 S'
_chemical_formula_weight          388.24

loop_
 _atom_type_symbol
 _atom_type_description
 _atom_type_scat_dispersion_real
 _atom_type_scat_dispersion_imag
 _atom_type_scat_source
 'C'  'C'   0.0033   0.0016
 'International Tables Vol C Tables 4.2.6.8 and 6.1.1.4'
 'H'  'H'   0.0000   0.0000
 'International Tables Vol C Tables 4.2.6.8 and 6.1.1.4'
 'N'  'N'   0.0061   0.0033
 'International Tables Vol C Tables 4.2.6.8 and 6.1.1.4'
 'O'  'O'   0.0106   0.0060
 'International Tables Vol C Tables 4.2.6.8 and 6.1.1.4'
 'F'  'F'   0.0171   0.0103
 'International Tables Vol C Tables 4.2.6.8 and 6.1.1.4'
 'S'  'S'   0.1246   0.1234
 'International Tables Vol C Tables 4.2.6.8 and 6.1.1.4'
 'Cl'  'Cl'   0.1484   0.1585
 'International Tables Vol C Tables 4.2.6.8 and 6.1.1.4'

_space_group_crystal_system       triclinic
_space_group_IT_number            2
_space_group_name_H-M_alt         'P -1'
_space_group_name_Hall            '-P 1'

_shelx_space_group_comment
;
The symmetry employed for this shelxl refinement is uniquely defined
by the following loop, which should always be used as a source of
symmetry information in preference to the above space-group names.
They are only intended as comments.
;

loop_
 _space_group_symop_operation_xyz
 'x, y, z'
 '-x, -y, -z'
```

_cell_length_a 7.3174(13)
_cell_length_b 8.2010(16)
_cell_length_c 15.629(2)
_cell_angle_alpha 82.251(14)
_cell_angle_beta 78.022(14)
_cell_angle_gamma 67.01(2)
_cell_volume 843.1(3)
_cell_formula_units_Z 2
_cell_measurement_temperature 296(2)
_cell_measurement_reflns_used 1110
_cell_measurement_theta_min 2.6770
_cell_measurement_theta_max 26.5420

_exptl_crystal_description plate
_exptl_crystal_colour colourless
_exptl_crystal_density_meas ?
_exptl_crystal_density_method ?
_exptl_crystal_density_diffrn 1.529
_exptl_crystal_F_000 396
_exptl_transmission_factor_min ?
_exptl_transmission_factor_max ?
_exptl_crystal_size_max 0.6
_exptl_crystal_size_mid 0.2
_exptl_crystal_size_min 0.1
_exptl_absorpt_coefficient_mu 0.532
_shelx_estimated_absorpt_T_min ?
_shelx_estimated_absorpt_T_max ?
_exptl_absorpt_correction_T_min 0.78247
_exptl_absorpt_correction_T_max 1.00000
_exptl_absorpt_correction_type 'multi-scan'
_exptl_absorpt_process_details
;
CrysAlisPro, Agilent Technologies,
Version 1.171.37.35h (release 09-02-2015 CrysAlis171 .NET)
(compiled Feb 9 2015,16:26:32)
Empirical absorption correction using spherical harmonics,
implemented in SCALE3 ABSPACK scaling algorithm.
;
_diffrn_ambient_temperature 296(2)
_diffrn_radiation_wavelength 0.71073
_diffrn_radiation_type MoK\a
_diffrn_source ?
_diffrn_measurement_device_type 'KM4 CCD four-circle diffractometer'
_diffrn_measurement_method '\w scans'
_diffrn_detector_area_resol_mean ?
_diffrn_reflns_number 5915
_diffrn_reflns_av_unetI/netI 0.0857
_diffrn_reflns_av_R_equivalents 0.0468
_diffrn_reflns_limit_h_min -9
_diffrn_reflns_limit_h_max 4
_diffrn_reflns_limit_k_min -10
_diffrn_reflns_limit_k_max 8
_diffrn_reflns_limit_l_min -20

_diffrn_reflns_limit_l_max 18
_diffrn_reflns_theta_min 2.669
_diffrn_reflns_theta_max 28.805
_diffrn_reflns_theta_full 25.242
_diffrn_measured_fraction_theta_max 0.856
_diffrn_measured_fraction_theta_full 0.996
_diffrn_reflns_Laue_measured_fraction_max 0.856
_diffrn_reflns_Laue_measured_fraction_full 0.996
_diffrn_reflns_point_group_measured_fraction_max 0.856
_diffrn_reflns_point_group_measured_fraction_full 0.996
_reflns_number_total 3773
_reflns_number_gt 1954
_reflns_threshold_expression 'I > 2\s(I)'
_reflns_Friedel_coverage 0.000
_reflns_Friedel_fraction_max .
_reflns_Friedel_fraction_full .

_reflns_special_details
;
Reflections were merged by SHELXL according to the crystal
class for the calculation of statistics and refinement.

_reflns_Friedel_fraction is defined as the number of unique
Friedel pairs measured divided by the number that would be
possible theoretically, ignoring centric projections and
systematic absences.
;

_computing_data_collection
;
CrysAlisPro, Agilent Technologies,
Version 1.171.37.35h (release 09-02-2015 CrysAlis171 .NET)
(compiled Feb 9 2015,16:26:32)
;
_computing_cell_refinement
;
CrysAlisPro, Agilent Technologies,
Version 1.171.37.35h (release 09-02-2015 CrysAlis171 .NET)
(compiled Feb 9 2015,16:26:32)
;
_computing_data_reduction
;
CrysAlisPro, Agilent Technologies,
Version 1.171.37.35h (release 09-02-2015 CrysAlis171 .NET)
(compiled Feb 9 2015,16:26:32)
;
_computing_structure_solution 'Sir 92'
_computing_structure_refinement 'SHELXL-2014/7 (Sheldrick, 2014)'
_computing_molecular_graphics ?
_computing_publication_material ?
_refine_special_details ?
_refine_ls_structure_factor_coef Fsqd
_refine_ls_matrix_type full

_refine_ls_weighting_scheme calc
_refine_ls_weighting_details
'w=1/[\s^2^(Fo^2^)+(0.0965P)^2^+0.0499P] where P=(Fo^2^+2Fc^2^)/3'
_atom_sites_solution_primary refxyz
_atom_sites_solution_secondary ?
_atom_sites_solution_hydrogens mixed
_refine_ls_hydrogen_treatment mixed
_refine_ls_extinction_method none
_refine_ls_extinction_coef .
_refine_ls_number_reflns 3773
_refine_ls_number_parameters 226
_refine_ls_number_restraints 0
_refine_ls_R_factor_all 0.1353
_refine_ls_R_factor_gt 0.0678
_refine_ls_wR_factor_ref 0.2275
_refine_ls_wR_factor_gt 0.1588
_refine_ls_goodness_of_fit_ref 1.011
_refine_ls_restrained_S_all 1.011
_refine_ls_shift/su_max 0.000
_refine_ls_shift/su_mean 0.000

loop_
_atom_site_label
_atom_site_type_symbol
_atom_site_fract_x
_atom_site_fract_y
_atom_site_fract_z
_atom_site_U_iso_or_equiv
_atom_site_adp_type
_atom_site_occupancy
_atom_site_site_symmetry_order
_atom_site_calc_flag
_atom_site_refinement_flags_posn
_atom_site_refinement_flags_adp
_atom_site_refinement_flags_occupancy
_atom_site_disorder_assembly
_atom_site_disorder_group
C2 C 0.2206(6) 0.6428(5) 0.1250(3) 0.0481(10) Uani 1 1 d . . . . .
C6 C 0.1078(6) 0.7493(6) -0.0927(3) 0.0505(10) Uani 1 1 d . . . . .
C8 C 0.1430(7) 0.6477(5) -0.1719(3) 0.0553(11) Uani 1 1 d . . . . .
H8A H 0.0159 0.6734 -0.1909 0.083 Uiso 1 1 calc R U . . .
H8B H 0.2308 0.6830 -0.2196 0.083 Uiso 1 1 calc R U . . .
C21 C 0.2217(6) 0.7131(6) 0.2775(3) 0.0511(10) Uani 1 1 d . . . . .
C22 C 0.1902(7) 0.8574(6) 0.3238(3) 0.0593(12) Uani 1 1 d . . . . .
C23 C 0.2036(8) 0.8461(7) 0.4105(3) 0.0732(14) Uani 1 1 d . . . . .
H23 H 0.1790 0.9468 0.4392 0.110 Uiso 1 1 calc R U . . .
C24 C 0.2549(8) 0.6800(7) 0.4546(3) 0.0781(15) Uani 1 1 d . . . . .
H24 H 0.2668 0.6677 0.5135 0.117 Uiso 1 1 calc R U . . .
C25 C 0.2879(8) 0.5346(7) 0.4112(3) 0.0732(14) Uani 1 1 d . . . . .
H25 H 0.3202 0.4240 0.4414 0.110 Uiso 1 1 calc R U . . .
C26 C 0.2744(7) 0.5479(6) 0.3227(3) 0.0659(13) Uani 1 1 d . . . . .
H26 H 0.3006 0.4468 0.2940 0.099 Uiso 1 1 calc R U . . .
C31 C 0.2613(6) 0.3471(5) -0.2104(3) 0.0474(10) Uani 1 1 d . . . . .

C32 C 0.3406(6) 0.1683(5) -0.1835(3) 0.0514(10) Uani 1 1 d . . . . .
C33 C 0.3762(6) 0.0392(5) -0.2386(3) 0.0555(11) Uani 1 1 d . . . . .
H33 H 0.4313 -0.0800 -0.2203 0.083 Uiso 1 1 calc R U . . .
C34 C 0.3295(7) 0.0875(6) -0.3216(3) 0.0577(11) Uani 1 1 d . . . . .
C35 C 0.2507(7) 0.2638(6) -0.3507(3) 0.0596(12) Uani 1 1 d . . . . .
H35 H 0.2200 0.2951 -0.4069 0.089 Uiso 1 1 calc R U . . .
C36 C 0.2183(6) 0.3932(6) -0.2944(3) 0.0543(11) Uani 1 1 d . . . . .
H36 H 0.1672 0.5123 -0.3135 0.081 Uiso 1 1 calc R U . . .
N1 N 0.1907(6) 0.7525(5) 0.1891(2) 0.0535(9) Uani 1 1 d . . . . .
H1 H 0.127(7) 0.873(7) 0.178(3) 0.080 Uiso 1 1 d . U . . .
N4 N 0.1476(6) 0.7371(5) 0.0536(2) 0.0563(10) Uani 1 1 d . . . . .
H4 H 0.105(8) 0.842(7) 0.050(3) 0.085 Uiso 1 1 d . U . . .
N5 N 0.1652(6) 0.6539(5) -0.0208(2) 0.0531(9) Uani 1 1 d . . . . .
H5 H 0.231(8) 0.553(6) -0.018(3) 0.080 Uiso 1 1 d . U . . .
O7 O 0.0273(5) 0.9126(4) -0.09606(18) 0.0644(9) Uani 1 1 d . . . . .
O9 O 0.2328(5) 0.4635(4) -0.14973(19) 0.0617(8) Uani 1 1 d . . . . .
F22 F 0.1367(4) 1.0207(3) 0.27979(17) 0.0774(8) Uani 1 1 d . . . . .
S3 S 0.33308(18) 0.42123(14) 0.12621(7) 0.0595(4) Uani 1 1 d . . . . .
Cl32 Cl 0.3970(2) 0.11022(16) -0.07871(8) 0.0750(4) Uani 1 1 d . . . . .
Cl34 Cl 0.3692(3) -0.07631(19) -0.39167(10) 0.0917(5) Uani 1 1 d . . . . .

loop_
 _atom_site_aniso_label
 _atom_site_aniso_U_11
 _atom_site_aniso_U_22
 _atom_site_aniso_U_33
 _atom_site_aniso_U_23
 _atom_site_aniso_U_13
 _atom_site_aniso_U_12
C2 0.042(2) 0.049(2) 0.045(2) 0.0017(18) -0.0088(18) -0.0099(19)
C6 0.048(2) 0.051(2) 0.049(2) 0.0023(19) -0.0103(19) -0.015(2)
C8 0.068(3) 0.044(2) 0.052(3) -0.0021(18) -0.016(2) -0.015(2)
C21 0.047(2) 0.060(3) 0.041(2) -0.0055(19) -0.0077(18) -0.012(2)
C22 0.057(3) 0.056(3) 0.055(3) -0.005(2) -0.007(2) -0.011(2)
C23 0.076(3) 0.078(3) 0.058(3) -0.019(3) -0.018(3) -0.012(3)
C24 0.087(4) 0.083(4) 0.052(3) -0.004(3) -0.022(3) -0.013(3)
C25 0.079(4) 0.065(3) 0.052(3) 0.008(2) -0.014(3) -0.005(3)
C26 0.074(3) 0.061(3) 0.052(3) 0.002(2) -0.009(2) -0.016(3)
C31 0.053(2) 0.043(2) 0.043(2) -0.0031(17) -0.0115(19) -0.0127(19)
C32 0.048(2) 0.048(2) 0.054(3) 0.0001(19) -0.014(2) -0.012(2)
C33 0.055(3) 0.044(2) 0.057(3) -0.003(2) -0.010(2) -0.007(2)
C34 0.060(3) 0.056(3) 0.054(3) -0.015(2) -0.005(2) -0.018(2)
C35 0.066(3) 0.068(3) 0.043(2) -0.005(2) -0.011(2) -0.022(3)
C36 0.061(3) 0.045(2) 0.051(3) 0.0004(19) -0.013(2) -0.013(2)
N1 0.061(2) 0.0426(19) 0.049(2) 0.0005(16) -0.0165(17) -0.0074(17)
N4 0.074(3) 0.046(2) 0.0390(19) -0.0045(16) -0.0198(18) -0.007(2)
N5 0.062(2) 0.0420(19) 0.045(2) -0.0001(16) -0.0135(17) -0.0068(18)
O7 0.093(2) 0.0392(16) 0.0544(18) 0.0000(13) -0.0240(16) -0.0123(16)
O9 0.086(2) 0.0407(16) 0.0536(18) -0.0047(13) -0.0265(16) -0.0113(15)
F22 0.101(2) 0.0525(16) 0.0723(19) -0.0028(13) -0.0290(16) -0.0155(15)
S3 0.0625(8) 0.0463(6) 0.0578(7) -0.0024(5) -0.0150(6) -0.0055(5)
Cl32 0.0971(10) 0.0647(8) 0.0578(7) 0.0106(6) -0.0364(7) -0.0175(7)
Cl34 0.1134(12) 0.0716(9) 0.0793(9) -0.0313(7) -0.0160(8) -0.0148(8)

_geom_special_details
;
 All esds (except the esd in the dihedral angle between two l.s. planes)
 are estimated using the full covariance matrix. The cell esds are taken
 into account individually in the estimation of esds in distances, angles
 and torsion angles; correlations between esds in cell parameters are only
 used when they are defined by crystal symmetry. An approximate (isotropic)
 treatment of cell esds is used for estimating esds involving l.s. planes.
;

loop_
 _geom_bond_atom_site_label_1
 _geom_bond_atom_site_label_2
 _geom_bond_distance
 _geom_bond_site_symmetry_2
 _geom_bond_publ_flag
C2 N4 1.350(5) . ?
C2 N1 1.363(5) . ?
C2 S3 1.676(4) . ?
C6 O7 1.233(5) . ?
C6 N5 1.323(5) . ?
C6 C8 1.507(5) . ?
C8 O9 1.418(5) . ?
C8 H8A 0.9700 . ?
C8 H8B 0.9700 . ?
C21 C22 1.388(6) . ?
C21 C26 1.389(6) . ?
C21 N1 1.420(5) . ?
C22 C23 1.367(6) . ?
C22 F22 1.369(5) . ?
C23 C24 1.388(7) . ?
C23 H23 0.9300 . ?
C24 C25 1.366(7) . ?
C24 H24 0.9300 . ?
C25 C26 1.394(6) . ?
C25 H25 0.9300 . ?
C26 H26 0.9300 . ?
C31 O9 1.364(5) . ?
C31 C36 1.383(6) . ?
C31 C32 1.390(6) . ?
C32 C33 1.368(5) . ?
C32 Cl32 1.730(4) . ?
C33 C34 1.376(6) . ?
C33 H33 0.9300 . ?
C34 C35 1.382(6) . ?
C34 Cl34 1.746(4) . ?
C35 C36 1.387(6) . ?
C35 H35 0.9300 . ?
C36 H36 0.9300 . ?
N1 H1 0.92(5) . ?
N4 N5 1.385(5) . ?
N4 H4 0.79(5) . ?

N5 H5 0.78(5) . ?

loop_
 _geom_angle_atom_site_label_1
 _geom_angle_atom_site_label_2
 _geom_angle_atom_site_label_3
 _geom_angle
 _geom_angle_site_symmetry_1
 _geom_angle_site_symmetry_3
 _geom_angle_publ_flag
N4 C2 N1 110.7(3) . . ?
N4 C2 S3 120.7(3) . . ?
N1 C2 S3 128.6(3) . . ?
O7 C6 N5 123.0(4) . . ?
O7 C6 C8 120.5(4) . . ?
N5 C6 C8 116.5(4) . . ?
O9 C8 C6 109.0(3) . . ?
O9 C8 H8A 109.9 . . ?
C6 C8 H8A 109.9 . . ?
O9 C8 H8B 109.9 . . ?
C6 C8 H8B 109.9 . . ?
H8A C8 H8B 108.3 . . ?
C22 C21 C26 117.0(4) . . ?
C22 C21 N1 115.7(4) . . ?
C26 C21 N1 127.2(4) . . ?
C23 C22 F22 118.7(4) . . ?
C23 C22 C21 124.0(4) . . ?
F22 C22 C21 117.3(4) . . ?
C22 C23 C24 118.1(5) . . ?
C22 C23 H23 121.0 . . ?
C24 C23 H23 121.0 . . ?
C25 C24 C23 119.7(5) . . ?
C25 C24 H24 120.2 . . ?
C23 C24 H24 120.2 . . ?
C24 C25 C26 121.6(5) . . ?
C24 C25 H25 119.2 . . ?
C26 C25 H25 119.2 . . ?
C21 C26 C25 119.6(5) . . ?
C21 C26 H26 120.2 . . ?
C25 C26 H26 120.2 . . ?
O9 C31 C36 125.4(4) . . ?
O9 C31 C32 115.8(4) . . ?
C36 C31 C32 118.8(4) . . ?
C33 C32 C31 121.1(4) . . ?
C33 C32 Cl32 120.0(3) . . ?
C31 C32 Cl32 118.9(3) . . ?
C32 C33 C34 119.3(4) . . ?
C32 C33 H33 120.4 . . ?
C34 C33 H33 120.4 . . ?
C33 C34 C35 121.3(4) . . ?
C33 C34 Cl34 119.6(4) . . ?
C35 C34 Cl34 119.1(4) . . ?
C34 C35 C36 118.7(4) . . ?

C34 C35 H35 120.6 . . ?
C36 C35 H35 120.6 . . ?
C31 C36 C35 120.7(4) . . ?
C31 C36 H36 119.6 . . ?
C35 C36 H36 119.6 . . ?
C2 N1 C21 130.6(4) . . ?
C2 N1 H1 118(3) . . ?
C21 N1 H1 111(3) . . ?
C2 N4 N5 121.0(3) . . ?
C2 N4 H4 122(4) . . ?
N5 N4 H4 116(4) . . ?
C6 N5 N4 120.2(3) . . ?
C6 N5 H5 126(4) . . ?
N4 N5 H5 113(4) . . ?
C31 O9 C8 118.3(3) . . ?

loop_
 _geom_torsion_atom_site_label_1
 _geom_torsion_atom_site_label_2
 _geom_torsion_atom_site_label_3
 _geom_torsion_atom_site_label_4
 _geom_torsion
 _geom_torsion_site_symmetry_1
 _geom_torsion_site_symmetry_2
 _geom_torsion_site_symmetry_3
 _geom_torsion_site_symmetry_4
 _geom_torsion_publ_flag
O7 C6 C8 O9 -178.7(4) . . . . ?
N5 C6 C8 O9 0.4(5) . . . . ?
C26 C21 C22 C23 -1.6(7) . . . . ?
N1 C21 C22 C23 176.8(4) . . . . ?
C26 C21 C22 F22 -179.5(4) . . . . ?
N1 C21 C22 F22 -1.1(6) . . . . ?
F22 C22 C23 C24 179.0(4) . . . . ?
C21 C22 C23 C24 1.1(8) . . . . ?
C22 C23 C24 C25 -0.7(8) . . . . ?
C23 C24 C25 C26 1.0(8) . . . . ?
C22 C21 C26 C25 1.7(7) . . . . ?
N1 C21 C26 C25 -176.5(4) . . . . ?
C24 C25 C26 C21 -1.5(8) . . . . ?
O9 C31 C32 C33 -179.9(4) . . . . ?
C36 C31 C32 C33 0.0(6) . . . . ?
O9 C31 C32 Cl32 -0.5(5) . . . . ?
C36 C31 C32 Cl32 179.3(3) . . . . ?
C31 C32 C33 C34 -1.0(7) . . . . ?
Cl32 C32 C33 C34 179.6(3) . . . . ?
C32 C33 C34 C35 1.1(7) . . . . ?
C32 C33 C34 Cl34 -178.6(3) . . . . ?
C33 C34 C35 C36 -0.1(7) . . . . ?
Cl34 C34 C35 C36 179.6(3) . . . . ?
O9 C31 C36 C35 -179.1(4) . . . . ?
C32 C31 C36 C35 1.0(6) . . . . ?
C34 C35 C36 C31 -0.9(7) . . . . ?

N4 C2 N1 C21 172.2(4) . . . . ?
S3 C2 N1 C21 -8.7(7) . . . . ?
C22 C21 N1 C2 175.3(4) . . . . ?
C26 C21 N1 C2 -6.5(7) . . . . ?
N1 C2 N4 N5 178.5(4) . . . . ?
S3 C2 N4 N5 -0.6(6) . . . . ?
O7 C6 N5 N4 -4.1(6) . . . . ?
C8 C6 N5 N4 176.8(4) . . . . ?
C2 N4 N5 C6 -174.4(4) . . . . ?
C36 C31 O9 C8 3.2(6) . . . . ?
C32 C31 O9 C8 -176.9(4) . . . . ?
C6 C8 O9 C31 174.5(3) . . . . ?

```
_refine_diff_density_max    0.354
_refine_diff_density_min   -0.358
_refine_diff_density_rms    0.071

_shelx_res_file
;

    shelx.res created by SHELXL-2014/7


TITL SIR92 run in space group P -1
CELL  0.71073   7.3174   8.2010  15.6288   82.251   78.022   67.010
ZERR     2.00   0.0013   0.0016   0.0021    0.014    0.014    0.020
LATT   1
SFAC  C    H    N    O    F    S    CL
UNIT  30 24 6 4 2 2 4
MERG   2
FMAP   2
PLAN   -10
ACTA
BOND   $H
CONF
L.S.  30
WGHT    0.096500    0.049900
FVAR       2.73510
C2    1    0.220626    0.642811    0.125039    11.00000    0.04234    0.04917 =
         0.04527    0.00170   -0.00879   -0.00994
C6    1    0.107835    0.749289   -0.092679    11.00000    0.04835    0.05054 =
         0.04882    0.00230   -0.01026   -0.01501
C8    1    0.142958    0.647653   -0.171879    11.00000    0.06759    0.04368 =
         0.05159   -0.00213   -0.01647   -0.01462
AFIX  23
H8A   2    0.015933    0.673443   -0.190877    11.00000   -1.50000
H8B   2    0.230779    0.682957   -0.219583    11.00000   -1.50000
AFIX   0
C21   1    0.221660    0.713068    0.277507    11.00000    0.04656    0.05968 =
         0.04059   -0.00550   -0.00770   -0.01195
C22   1    0.190213    0.857373    0.323809    11.00000    0.05692    0.05607 =
         0.05469   -0.00455   -0.00692   -0.01113
C23   1    0.203595    0.846116    0.410544    11.00000    0.07613    0.07772 =
```

0.05772 -0.01899 -0.01757 -0.01218
AFIX 43
H23 2 0.179032 0.946840 0.439158 11.00000 -1.50000
AFIX 0
C24 1 0.254917 0.679953 0.454644 11.00000 0.08663 0.08252 =
0.05246 -0.00369 -0.02214 -0.01314
AFIX 43
H24 2 0.266767 0.667661 0.513494 11.00000 -1.50000
AFIX 0
C25 1 0.287924 0.534604 0.411184 11.00000 0.07950 0.06506 =
0.05221 0.00803 -0.01408 -0.00482
AFIX 43
H25 2 0.320209 0.424015 0.441407 11.00000 -1.50000
AFIX 0
C26 1 0.274426 0.547889 0.322656 11.00000 0.07381 0.06066 =
0.05233 0.00221 -0.00865 -0.01643
AFIX 43
H26 2 0.300587 0.446791 0.294019 11.00000 -1.50000
AFIX 0
C31 1 0.261316 0.347059 -0.210353 11.00000 0.05318 0.04292 =
0.04304 -0.00307 -0.01151 -0.01274
C32 1 0.340640 0.168348 -0.183478 11.00000 0.04798 0.04824 =
0.05392 0.00009 -0.01360 -0.01213
C33 1 0.376171 0.039212 -0.238630 11.00000 0.05488 0.04355 =
0.05737 -0.00325 -0.01011 -0.00676
AFIX 43
H33 2 0.431281 -0.079982 -0.220277 11.00000 -1.50000
AFIX 0
C34 1 0.329507 0.087531 -0.321570 11.00000 0.06043 0.05636 =
0.05357 -0.01478 -0.00460 -0.01777
C35 1 0.250704 0.263790 -0.350672 11.00000 0.06610 0.06802 =
0.04344 -0.00541 -0.01144 -0.02206
AFIX 43
H35 2 0.219955 0.295053 -0.406901 11.00000 -1.50000
AFIX 0
C36 1 0.218339 0.393178 -0.294449 11.00000 0.06142 0.04508 =
0.05120 0.00038 -0.01329 -0.01345
AFIX 43
H36 2 0.167175 0.512277 -0.313485 11.00000 -1.50000
AFIX 0
N1 3 0.190740 0.752467 0.189114 11.00000 0.06061 0.04257 =
0.04872 0.00048 -0.01654 -0.00743
H1 2 0.126818 0.872675 0.177614 11.00000 -1.50000
N4 3 0.147616 0.737145 0.053584 11.00000 0.07392 0.04642 =
0.03903 -0.00447 -0.01975 -0.00651
H4 2 0.105274 0.841547 0.049600 11.00000 -1.50000
N5 3 0.165239 0.653940 -0.020846 11.00000 0.06183 0.04204 =
0.04481 -0.00014 -0.01345 -0.00680
H5 2 0.231366 0.552580 -0.017922 11.00000 -1.50000
O7 4 0.027253 0.912552 -0.096064 11.00000 0.09263 0.03919 =
0.05445 -0.00001 -0.02396 -0.01230
O9 4 0.232821 0.463490 -0.149730 11.00000 0.08644 0.04065 =
0.05361 -0.00466 -0.02649 -0.01127

```
F22   5    0.136727    1.020692    0.279788    11.00000    0.10140    0.05251 =
        0.07226   -0.00284   -0.02904   -0.01545
S3    6    0.333081    0.421226    0.126213    11.00000    0.06245    0.04634 =
        0.05779   -0.00238   -0.01498   -0.00552
CL32  7    0.396973    0.110223   -0.078711    11.00000    0.09713    0.06473 =
        0.05783    0.01063   -0.03641   -0.01751
CL34  7    0.369236   -0.076307   -0.391672    11.00000    0.11343    0.07162 =
        0.07929   -0.03133   -0.01600   -0.01476

HKLF    4

REM  SIR92 run in space group P -1
REM R1 =  0.0678 for    1954 Fo > 4sig(Fo)  and  0.1353 for all    3773 data
REM    226 parameters refined using      0 restraints

END

WGHT      0.0899      0.0000

REM Highest difference peak  0.354,  deepest hole -0.358,  1-sigma level  0.071
Q1    1   0.4668 -0.0614 -0.0517  11.00000  0.05    0.35
Q2    1   0.1211  1.0457  0.2056  11.00000  0.05    0.32
Q3    1   0.5270 -0.0777 -0.4115  11.00000  0.05    0.26
Q4    1   0.4157  0.0666 -0.0034  11.00000  0.05    0.26
Q5    1   0.4971  0.2470 -0.0201  11.00000  0.05    0.26
Q6    1   0.3743  0.4009  0.1943  11.00000  0.05    0.25
Q7    1   0.1689  0.9977  0.4314  11.00000  0.05    0.25
Q8    1   0.3971  0.1427 -0.1584  11.00000  0.05    0.25
Q9    1   0.4259 -0.0978 -0.3266  11.00000  0.05    0.25
Q10   1   0.5331 -0.1012 -0.3889  11.00000  0.05    0.25
;
```

PK2

```
data_shelx

_audit_creation_method            'SHELXL-2014/7'
_shelx_SHELXL_version_number      '2014/7'
_chemical_name_systematic         ?
_chemical_name_common             ?
_chemical_melting_point           ?
_chemical_formula_moiety          'C16 H15 Cl2 N3 O2 S2'
_chemical_formula_sum             'C16 H15 Cl2 N3 O2 S2'
_chemical_formula_weight          416.33

loop_
 _atom_type_symbol
 _atom_type_description
 _atom_type_scat_dispersion_real
 _atom_type_scat_dispersion_imag
 _atom_type_scat_source
```

'C' 'C' 0.0033 0.0016
'International Tables Vol C Tables 4.2.6.8 and 6.1.1.4'
'H' 'H' 0.0000 0.0000
'International Tables Vol C Tables 4.2.6.8 and 6.1.1.4'
'N' 'N' 0.0061 0.0033
'International Tables Vol C Tables 4.2.6.8 and 6.1.1.4'
'O' 'O' 0.0106 0.0060
'International Tables Vol C Tables 4.2.6.8 and 6.1.1.4'
'S' 'S' 0.1246 0.1234
'International Tables Vol C Tables 4.2.6.8 and 6.1.1.4'
'Cl' 'Cl' 0.1484 0.1585
'International Tables Vol C Tables 4.2.6.8 and 6.1.1.4'

_space_group_crystal_system triclinic
_space_group_IT_number 2
_space_group_name_H-M_alt 'P -1'
_space_group_name_Hall '-P 1'

_shelx_space_group_comment
;
The symmetry employed for this shelxl refinement is uniquely defined
by the following loop, which should always be used as a source of
symmetry information in preference to the above space-group names.
They are only intended as comments.
;

loop_
_space_group_symop_operation_xyz
'x, y, z'
'-x, -y, -z'

_cell_length_a 9.1146(7)
_cell_length_b 9.6738(8)
_cell_length_c 10.6620(8)
_cell_angle_alpha 85.735(6)
_cell_angle_beta 79.293(6)
_cell_angle_gamma 76.631(7)
_cell_volume 898.21(13)
_cell_formula_units_Z 2
_cell_measurement_temperature 296(2)
_cell_measurement_reflns_used 2425
_cell_measurement_theta_min 2.1860
_cell_measurement_theta_max 28.6400

_exptl_crystal_description block
_exptl_crystal_colour colourless
_exptl_crystal_density_meas ?
_exptl_crystal_density_method ?
_exptl_crystal_density_diffrn 1.539
_exptl_crystal_F_000 428
_exptl_transmission_factor_min ?
_exptl_transmission_factor_max ?
_exptl_crystal_size_max 0.60

_exptl_crystal_size_mid 0.50
_exptl_crystal_size_min 0.40
_exptl_absorpt_coefficient_mu 0.609
_shelx_estimated_absorpt_T_min ?
_shelx_estimated_absorpt_T_max ?
_exptl_absorpt_correction_T_min 0.86045
_exptl_absorpt_correction_T_max 1.00000
_exptl_absorpt_correction_type 'multi-scan'
_exptl_absorpt_process_details
;
CrysAlisPro, Agilent Technologies,
Version 1.171.37.35h (release 09-02-2015 CrysAlis171 .NET)
(compiled Feb 9 2015,16:26:32)
Empirical absorption correction using spherical harmonics,
implemented in SCALE3 ABSPACK scaling algorithm.
;
_exptl_absorpt_special_details ?
_diffrn_ambient_temperature 296(2)
_diffrn_radiation_wavelength 0.71073
_diffrn_radiation_type MoK\a
_diffrn_source ?
_diffrn_measurement_device_type 'KM4 CCD four-circle diffractometer'
_diffrn_measurement_method ?
_diffrn_detector_area_resol_mean ?
_diffrn_reflns_number 6597
_diffrn_reflns_av_unetI/netI 0.0395
_diffrn_reflns_av_R_equivalents 0.0216
_diffrn_reflns_limit_h_min -11
_diffrn_reflns_limit_h_max 11
_diffrn_reflns_limit_k_min -12
_diffrn_reflns_limit_k_max 12
_diffrn_reflns_limit_l_min -12
_diffrn_reflns_limit_l_max 13
_diffrn_reflns_theta_min 2.165
_diffrn_reflns_theta_max 28.846
_diffrn_reflns_theta_full 25.242
_diffrn_measured_fraction_theta_max 0.863
_diffrn_measured_fraction_theta_full 1.000
_diffrn_reflns_Laue_measured_fraction_max 0.863
_diffrn_reflns_Laue_measured_fraction_full 1.000
_diffrn_reflns_point_group_measured_fraction_max 0.863
_diffrn_reflns_point_group_measured_fraction_full 1.000
_reflns_number_total 4067
_reflns_number_gt 3088
_reflns_threshold_expression 'I > 2\s(I)'
_reflns_Friedel_coverage 0.000
_reflns_Friedel_fraction_max .
_reflns_Friedel_fraction_full .

_reflns_special_details
;
Reflections were merged by SHELXL according to the crystal
class for the calculation of statistics and refinement.

_reflns_Friedel_fraction is defined as the number of unique
Friedel pairs measured divided by the number that would be
possible theoretically, ignoring centric projections and
systematic absences.
;

_computing_data_collection
;
CrysAlisPro, Agilent Technologies,
Version 1.171.37.35h (release 09-02-2015 CrysAlis171 .NET)
(compiled Feb 9 2015,16:26:32)
;
_computing_cell_refinement
;
CrysAlisPro, Agilent Technologies,
Version 1.171.37.35h (release 09-02-2015 CrysAlis171 .NET)
(compiled Feb 9 2015,16:26:32)
;
_computing_data_reduction
;
CrysAlisPro, Agilent Technologies,
Version 1.171.37.35h (release 09-02-2015 CrysAlis171 .NET)
(compiled Feb 9 2015,16:26:32)
;
_computing_structure_solution ?
_computing_structure_refinement 'SHELXL-2014/7 (Sheldrick, 2014)'
_computing_molecular_graphics ?
_computing_publication_material ?
_refine_special_details ?
_refine_ls_structure_factor_coef Fsqd
_refine_ls_matrix_type full
_refine_ls_weighting_scheme calc
_refine_ls_weighting_details
'w=1/[\s^2^(Fo^2^)+(0.0616P)^2^+0.0996P] where P=(Fo^2^+2Fc^2^)/3'
_atom_sites_solution_primary direct
_atom_sites_solution_secondary difmap
_atom_sites_solution_hydrogens difmap
_refine_ls_hydrogen_treatment mixed
_refine_ls_extinction_method 'SHELXL-2014/7 (Sheldrick 2014'
_refine_ls_extinction_coef 0.0065(19)
_refine_ls_extinction_expression
'Fc^*^=kFc[1+0.001xFc^2^\l^3^/sin(2\q)]^-1/4^'
_refine_ls_number_reflns 4067
_refine_ls_number_parameters 236
_refine_ls_number_restraints 0
_refine_ls_R_factor_all 0.0564
_refine_ls_R_factor_gt 0.0391
_refine_ls_wR_factor_ref 0.1196
_refine_ls_wR_factor_gt 0.1068
_refine_ls_goodness_of_fit_ref 1.026
_refine_ls_restrained_S_all 1.026
_refine_ls_shift/su_max 0.001

_refine_ls_shift/su_mean 0.000

loop_
 _atom_site_label
 _atom_site_type_symbol
 _atom_site_fract_x
 _atom_site_fract_y
 _atom_site_fract_z
 _atom_site_U_iso_or_equiv
 _atom_site_adp_type
 _atom_site_occupancy
 _atom_site_site_symmetry_order
 _atom_site_calc_flag
 _atom_site_refinement_flags_posn
 _atom_site_refinement_flags_adp
 _atom_site_refinement_flags_occupancy
 _atom_site_disorder_assembly
 _atom_site_disorder_group
C2 C 0.4590(2) 0.2092(2) 0.3819(2) 0.0393(5) Uani 1 1 d . . . . .
C6 C 0.2303(2) 0.4713(2) 0.59658(19) 0.0359(5) Uani 1 1 d . . . . .
C8 C 0.0659(2) 0.5008(2) 0.6644(2) 0.0407(5) Uani 1 1 d . . . . .
H81 H 0.0621 0.4930 0.7561 0.061 Uiso 1 1 calc R U . . .
H82 H 0.0138 0.5963 0.6425 0.061 Uiso 1 1 calc R U . . .
C21 C 0.6735(2) 0.0870(2) 0.2222(2) 0.0399(5) Uani 1 1 d . . . . .
C22 C 0.8026(3) -0.0106(3) 0.2403(2) 0.0490(6) Uani 1 1 d . . . . .
H22 H 0.8375 -0.0144 0.3173 0.074 Uiso 1 1 calc R U . . .
C23 C 0.8827(3) -0.1039(3) 0.1464(2) 0.0497(6) Uani 1 1 d . . . . .
H23 H 0.9704 -0.1698 0.1608 0.075 Uiso 1 1 calc R U . . .
C24 C 0.8330(2) -0.0997(2) 0.0309(2) 0.0394(5) Uani 1 1 d . . . . .
C25 C 0.7008(3) -0.0018(3) 0.0130(2) 0.0492(6) Uani 1 1 d . . . . .
H25 H 0.6657 0.0021 -0.0640 0.074 Uiso 1 1 calc R U . . .
C26 C 0.6203(3) 0.0903(3) 0.1078(2) 0.0495(6) Uani 1 1 d . . . . .
H26 H 0.5307 0.1544 0.0951 0.074 Uiso 1 1 calc R U . . .
C28 C 1.0950(3) -0.3096(3) -0.0439(3) 0.0703(8) Uani 1 1 d . . . . .
H281 H 1.1532 -0.3750 -0.1078 0.105 Uiso 1 1 calc R U . . .
H282 H 1.1549 -0.2444 -0.0298 0.105 Uiso 1 1 calc R U . . .
H283 H 1.0687 -0.3612 0.0344 0.105 Uiso 1 1 calc R U . . .
C31 C -0.1524(2) 0.3955(2) 0.68688(19) 0.0351(4) Uani 1 1 d . . . . .
C32 C -0.2088(2) 0.2806(2) 0.6594(2) 0.0358(5) Uani 1 1 d . . . . .
C33 C -0.3526(2) 0.2646(2) 0.7162(2) 0.0407(5) Uani 1 1 d . . . . .
H33 H -0.3890 0.1873 0.6983 0.061 Uiso 1 1 calc R U . . .
C34 C -0.4415(2) 0.3652(2) 0.8001(2) 0.0394(5) Uani 1 1 d . . . . .
C35 C -0.3901(2) 0.4812(2) 0.8265(2) 0.0424(5) Uani 1 1 d . . . . .
H35 H -0.4524 0.5491 0.8820 0.064 Uiso 1 1 calc R U . . .
C36 C -0.2445(2) 0.4957(2) 0.7697(2) 0.0412(5) Uani 1 1 d . . . . .
H36 H -0.2088 0.5734 0.7876 0.062 Uiso 1 1 calc R U . . .
N1 N 0.6032(2) 0.1899(2) 0.3168(2) 0.0490(5) Uani 1 1 d . . . . .
H1 H 0.656(3) 0.242(3) 0.338(3) 0.074 Uiso 1 1 d . U . . .
N4 N 0.4254(2) 0.3143(2) 0.46681(19) 0.0441(5) Uani 1 1 d . . . . .
H4 H 0.483(3) 0.371(3) 0.463(2) 0.066 Uiso 1 1 d . U . . .
N5 N 0.2786(2) 0.3514(2) 0.53477(18) 0.0422(5) Uani 1 1 d . . . . .
H5 H 0.223(3) 0.295(3) 0.534(2) 0.063 Uiso 1 1 d . U . . .
O7 O 0.31237(17) 0.55590(16) 0.60108(15) 0.0446(4) Uani 1 1 d . . . . .

O9 O -0.00717(17) 0.39962(16) 0.62584(14) 0.0454(4) Uani 1 1 d . . . . .
S3 S 0.33160(7) 0.11565(7) 0.36549(7) 0.0561(2) Uani 1 1 d . . . . .
S27 S 0.92471(8) -0.21336(8) -0.09638(6) 0.0574(2) Uani 1 1 d . . . . .
Cl32 Cl -0.09596(6) 0.15841(6) 0.55079(6) 0.05271(19) Uani 1 1 d . . . . .
Cl34 Cl -0.62152(7) 0.34292(7) 0.87563(6) 0.0570(2) Uani 1 1 d . . . . .

loop_
 _atom_site_aniso_label
 _atom_site_aniso_U_11
 _atom_site_aniso_U_22
 _atom_site_aniso_U_33
 _atom_site_aniso_U_23
 _atom_site_aniso_U_13
 _atom_site_aniso_U_12
C2 0.0369(11) 0.0353(11) 0.0485(13) -0.0096(9) -0.0075(9) -0.0113(9)
C6 0.0367(11) 0.0387(12) 0.0363(11) -0.0063(9) -0.0068(8) -0.0142(9)
C8 0.0399(11) 0.0438(12) 0.0430(12) -0.0147(10) -0.0001(9) -0.0204(9)
C21 0.0327(10) 0.0404(12) 0.0490(13) -0.0133(10) -0.0031(9) -0.0125(9)
C22 0.0468(13) 0.0574(15) 0.0441(13) -0.0109(11) -0.0123(10) -0.0076(11)
C23 0.0421(12) 0.0531(15) 0.0518(14) -0.0112(11) -0.0120(10) -0.0005(10)
C24 0.0380(11) 0.0406(12) 0.0423(12) -0.0084(9) -0.0016(9) -0.0163(9)
C25 0.0513(14) 0.0534(15) 0.0478(13) -0.0094(11) -0.0157(11) -0.0136(11)
C26 0.0418(12) 0.0458(14) 0.0625(15) -0.0104(11) -0.0179(11) -0.0034(10)
C28 0.0553(16) 0.0722(19) 0.079(2) -0.0291(15) -0.0003(14) -0.0053(14)
C31 0.0336(10) 0.0350(11) 0.0388(11) -0.0059(9) -0.0045(8) -0.0118(8)
C32 0.0347(10) 0.0296(10) 0.0450(12) -0.0064(9) -0.0094(9) -0.0072(8)
C33 0.0382(11) 0.0342(11) 0.0541(13) -0.0028(10) -0.0110(10) -0.0139(9)
C34 0.0330(10) 0.0415(12) 0.0446(12) 0.0036(10) -0.0062(9) -0.0122(9)
C35 0.0418(12) 0.0415(12) 0.0434(12) -0.0072(10) -0.0012(9) -0.0114(9)
C36 0.0435(12) 0.0368(11) 0.0466(12) -0.0100(9) -0.0032(9) -0.0166(9)
N1 0.0350(10) 0.0515(12) 0.0646(13) -0.0264(10) -0.0013(9) -0.0160(9)
N4 0.0327(9) 0.0445(11) 0.0584(12) -0.0202(9) 0.0024(8) -0.0175(8)
N5 0.0369(9) 0.0424(11) 0.0512(11) -0.0167(8) 0.0023(8) -0.0198(8)
O7 0.0416(8) 0.0430(9) 0.0554(9) -0.0133(7) -0.0035(7) -0.0220(7)
O9 0.0371(8) 0.0506(9) 0.0539(9) -0.0221(7) 0.0039(7) -0.0233(7)
S3 0.0461(3) 0.0520(4) 0.0766(5) -0.0296(3) 0.0038(3) -0.0264(3)
S27 0.0532(4) 0.0628(4) 0.0583(4) -0.0264(3) -0.0023(3) -0.0151(3)
Cl32 0.0440(3) 0.0410(3) 0.0739(4) -0.0255(3) -0.0047(3) -0.0080(2)
Cl34 0.0380(3) 0.0653(4) 0.0678(4) 0.0006(3) 0.0010(3) -0.0209(3)

_geom_special_details
;
 All esds (except the esd in the dihedral angle between two l.s. planes)
 are estimated using the full covariance matrix. The cell esds are taken
 into account individually in the estimation of esds in distances, angles
 and torsion angles; correlations between esds in cell parameters are only
 used when they are defined by crystal symmetry. An approximate (isotropic)
 treatment of cell esds is used for estimating esds involving l.s. planes.
;

loop_
 _geom_bond_atom_site_label_1
 _geom_bond_atom_site_label_2

_geom_bond_distance
_geom_bond_site_symmetry_2
_geom_bond_publ_flag
C2 N1 1.346(3) . ?
C2 N4 1.356(3) . ?
C2 S3 1.668(2) . ?
C6 O7 1.238(2) . ?
C6 N5 1.320(3) . ?
C6 C8 1.510(3) . ?
C8 O9 1.426(2) . ?
C8 H81 0.9700 . ?
C8 H82 0.9700 . ?
C21 C22 1.362(3) . ?
C21 C26 1.388(3) . ?
C21 N1 1.426(3) . ?
C22 C23 1.381(3) . ?
C22 H22 0.9300 . ?
C23 C24 1.383(3) . ?
C23 H23 0.9300 . ?
C24 C25 1.384(3) . ?
C24 S27 1.767(2) . ?
C25 C26 1.382(3) . ?
C25 H25 0.9300 . ?
C26 H26 0.9300 . ?
C28 S27 1.779(3) . ?
C28 H281 0.9600 . ?
C28 H282 0.9600 . ?
C28 H283 0.9600 . ?
C31 O9 1.370(2) . ?
C31 C36 1.378(3) . ?
C31 C32 1.398(3) . ?
C32 C33 1.376(3) . ?
C32 Cl32 1.731(2) . ?
C33 C34 1.378(3) . ?
C33 H33 0.9300 . ?
C34 C35 1.377(3) . ?
C34 Cl34 1.742(2) . ?
C35 C36 1.386(3) . ?
C35 H35 0.9300 . ?
C36 H36 0.9300 . ?
N1 H1 0.85(3) . ?
N4 N5 1.378(2) . ?
N4 H4 0.83(3) . ?
N5 H5 0.83(3) . ?

loop_
_geom_angle_atom_site_label_1
_geom_angle_atom_site_label_2
_geom_angle_atom_site_label_3
_geom_angle
_geom_angle_site_symmetry_1
_geom_angle_site_symmetry_3
_geom_angle_publ_flag

N1 C2 N4 112.67(18) . . ?
N1 C2 S3 125.51(16) . . ?
N4 C2 S3 121.81(16) . . ?
O7 C6 N5 123.4(2) . . ?
O7 C6 C8 120.72(19) . . ?
N5 C6 C8 115.85(17) . . ?
O9 C8 C6 108.46(16) . . ?
O9 C8 H81 110.0 . . ?
C6 C8 H81 110.0 . . ?
O9 C8 H82 110.0 . . ?
C6 C8 H82 110.0 . . ?
H81 C8 H82 108.4 . . ?
C22 C21 C26 119.1(2) . . ?
C22 C21 N1 119.2(2) . . ?
C26 C21 N1 121.6(2) . . ?
C21 C22 C23 121.3(2) . . ?
C21 C22 H22 119.4 . . ?
C23 C22 H22 119.4 . . ?
C22 C23 C24 120.3(2) . . ?
C22 C23 H23 119.8 . . ?
C24 C23 H23 119.8 . . ?
C23 C24 C25 118.5(2) . . ?
C23 C24 S27 124.65(18) . . ?
C25 C24 S27 116.89(17) . . ?
C26 C25 C24 121.0(2) . . ?
C26 C25 H25 119.5 . . ?
C24 C25 H25 119.5 . . ?
C25 C26 C21 119.9(2) . . ?
C25 C26 H26 120.0 . . ?
C21 C26 H26 120.0 . . ?
S27 C28 H281 109.5 . . ?
S27 C28 H282 109.5 . . ?
H281 C28 H282 109.5 . . ?
S27 C28 H283 109.5 . . ?
H281 C28 H283 109.5 . . ?
H282 C28 H283 109.5 . . ?
O9 C31 C36 125.04(17) . . ?
O9 C31 C32 115.87(17) . . ?
C36 C31 C32 119.08(18) . . ?
C33 C32 C31 121.00(19) . . ?
C33 C32 Cl32 119.89(16) . . ?
C31 C32 Cl32 119.10(16) . . ?
C32 C33 C34 118.73(19) . . ?
C32 C33 H33 120.6 . . ?
C34 C33 H33 120.6 . . ?
C35 C34 C33 121.42(19) . . ?
C35 C34 Cl34 119.65(17) . . ?
C33 C34 Cl34 118.92(16) . . ?
C34 C35 C36 119.4(2) . . ?
C34 C35 H35 120.3 . . ?
C36 C35 H35 120.3 . . ?
C31 C36 C35 120.33(19) . . ?
C31 C36 H36 119.8 . . ?

C35 C36 H36 119.8 . . ?
C2 N1 C21 126.24(18) . . ?
C2 N1 H1 114.9(19) . . ?
C21 N1 H1 118.8(19) . . ?
C2 N4 N5 118.90(17) . . ?
C2 N4 H4 120.0(19) . . ?
N5 N4 H4 118.6(19) . . ?
C6 N5 N4 121.26(17) . . ?
C6 N5 H5 122.3(19) . . ?
N4 N5 H5 116.5(19) . . ?
C31 O9 C8 118.54(15) . . ?
C24 S27 C28 103.43(12) . . ?

loop_
 _geom_torsion_atom_site_label_1
 _geom_torsion_atom_site_label_2
 _geom_torsion_atom_site_label_3
 _geom_torsion_atom_site_label_4
 _geom_torsion
 _geom_torsion_site_symmetry_1
 _geom_torsion_site_symmetry_2
 _geom_torsion_site_symmetry_3
 _geom_torsion_site_symmetry_4
 _geom_torsion_publ_flag
O7 C6 C8 O9 170.48(19) . . . . ?
N5 C6 C8 O9 -10.0(3) . . . . ?
C26 C21 C22 C23 1.2(4) . . . . ?
N1 C21 C22 C23 -174.0(2) . . . . ?
C21 C22 C23 C24 0.2(4) . . . . ?
C22 C23 C24 C25 -0.9(3) . . . . ?
C22 C23 C24 S27 -179.82(19) . . . . ?
C23 C24 C25 C26 0.3(3) . . . . ?
S27 C24 C25 C26 179.23(19) . . . . ?
C24 C25 C26 C21 1.2(4) . . . . ?
C22 C21 C26 C25 -1.9(4) . . . . ?
N1 C21 C26 C25 173.2(2) . . . . ?
O9 C31 C32 C33 179.25(19) . . . . ?
C36 C31 C32 C33 -1.7(3) . . . . ?
O9 C31 C32 Cl32 -1.5(3) . . . . ?
C36 C31 C32 Cl32 177.61(17) . . . . ?
C31 C32 C33 C34 0.8(3) . . . . ?
Cl32 C32 C33 C34 -178.51(16) . . . . ?
C32 C33 C34 C35 0.8(3) . . . . ?
C32 C33 C34 Cl34 -178.26(17) . . . . ?
C33 C34 C35 C36 -1.4(3) . . . . ?
Cl34 C34 C35 C36 177.64(17) . . . . ?
O9 C31 C36 C35 -180.0(2) . . . . ?
C32 C31 C36 C35 1.1(3) . . . . ?
C34 C35 C36 C31 0.4(3) . . . . ?
N4 C2 N1 C21 179.3(2) . . . . ?
S3 C2 N1 C21 0.2(4) . . . . ?
C22 C21 N1 C2 -121.0(3) . . . . ?
C26 C21 N1 C2 63.9(3) . . . . ?

N1 C2 N4 N5 176.2(2) . . . . ?
S3 C2 N4 N5 -4.7(3) . . . . ?
O7 C6 N5 N4 -2.0(3) . . . . ?
C8 C6 N5 N4 178.50(19) . . . . ?
C2 N4 N5 C6 -164.8(2) . . . . ?
C36 C31 O9 C8 10.6(3) . . . . ?
C32 C31 O9 C8 -170.36(18) . . . . ?
C6 C8 O9 C31 172.41(18) . . . . ?
C23 C24 S27 C28 -6.2(2) . . . . ?
C25 C24 S27 C28 174.88(19) . . . . ?

```
_refine_diff_density_max    0.256
_refine_diff_density_min   -0.300
_refine_diff_density_rms    0.047

_shelx_res_file
;

    shelx.res created by SHELXL-2014/7


TITL SIR92 run in space group P -1
CELL  0.71073   9.1146   9.6738  10.6620   85.735   79.293   76.631
ZERR     2.00   0.0007   0.0008   0.0008    0.006    0.006    0.007
LATT   1
SFAC  C    H    N    O    S    CL
UNIT  32 30 6 4 4 4
MERG   2
FMAP   2
PLAN   -2
OMIT   0  0  1
ACTA
BOND   $H
CONF
L.S.  80
TEMP    23.00
WGHT    0.061600    0.099600
EXTI    0.006535
FVAR       6.36053
C2    1    0.458978    0.209235    0.381875    11.00000    0.03694    0.03533 =
         0.04853   -0.00964   -0.00745   -0.01125
C6    1    0.230314    0.471338    0.596577    11.00000    0.03673    0.03868 =
         0.03627   -0.00629   -0.00679   -0.01420
C8    1    0.065941    0.500776    0.664354    11.00000    0.03990    0.04380 =
         0.04303   -0.01474   -0.00005   -0.02039
AFIX  23
H81   2    0.062132    0.493036    0.756078    11.00000   -1.50000
H82   2    0.013807    0.596340    0.642459    11.00000   -1.50000
AFIX   0
C21   1    0.673520    0.086951    0.222151    11.00000    0.03265    0.04041 =
         0.04905   -0.01330   -0.00306   -0.01247
C22   1    0.802632   -0.010585    0.240254    11.00000    0.04678    0.05745 =
         0.04406   -0.01088   -0.01228   -0.00760
```

```
AFIX  43
H22   2    0.837548   -0.014412    0.317314    11.00000   -1.50000
AFIX   0
C23   1    0.882658   -0.103889    0.146352    11.00000    0.04212    0.05312 =
         0.05178   -0.01124   -0.01201   -0.00047
AFIX  43
H23   2    0.970356   -0.169816    0.160821    11.00000   -1.50000
AFIX   0
C24   1    0.832986   -0.099739    0.030894    11.00000    0.03797    0.04057 =
         0.04227   -0.00836   -0.00161   -0.01633
C25   1    0.700828   -0.001805    0.013003    11.00000    0.05128    0.05337 =
         0.04784   -0.00936   -0.01571   -0.01365
AFIX  43
H25   2    0.665666    0.002088   -0.063953    11.00000   -1.50000
AFIX   0
C26   1    0.620348    0.090290    0.107833    11.00000    0.04181    0.04582 =
         0.06253   -0.01043   -0.01788   -0.00338
AFIX  43
H26   2    0.530716    0.154399    0.095093    11.00000   -1.50000
AFIX   0
C28   1    1.094957   -0.309558   -0.043864    11.00000    0.05533    0.07219 =
         0.07854   -0.02906   -0.00032   -0.00533
AFIX  33
H281  2    1.153196   -0.374999   -0.107803    11.00000   -1.50000
H282  2    1.154877   -0.244403   -0.029816    11.00000   -1.50000
H283  2    1.068667   -0.361182    0.034363    11.00000   -1.50000
AFIX   0
C31   1   -0.152426    0.395516    0.686880    11.00000    0.03360    0.03495 =
         0.03877   -0.00585   -0.00445   -0.01181
C32   1   -0.208832    0.280637    0.659385    11.00000    0.03467    0.02960 =
         0.04500   -0.00640   -0.00942   -0.00717
C33   1   -0.352579    0.264597    0.716193    11.00000    0.03816    0.03418 =
         0.05410   -0.00275   -0.01098   -0.01391
AFIX  43
H33   2   -0.389049    0.187337    0.698340    11.00000   -1.50000
AFIX   0
C34   1   -0.441515    0.365243    0.800084    11.00000    0.03301    0.04152 =
         0.04465    0.00358   -0.00617   -0.01222
C35   1   -0.390076    0.481166    0.826508    11.00000    0.04184    0.04152 =
         0.04335   -0.00718   -0.00122   -0.01138
AFIX  43
H35   2   -0.452439    0.549121    0.881956    11.00000   -1.50000
AFIX   0
C36   1   -0.244532    0.495678    0.769738    11.00000    0.04353    0.03677 =
         0.04655   -0.00995   -0.00320   -0.01659
AFIX  43
H36   2   -0.208843    0.573388    0.787644    11.00000   -1.50000
AFIX   0
N1    3    0.603231    0.189875    0.316787    11.00000    0.03500    0.05152 =
         0.06460   -0.02642   -0.00131   -0.01596
H1    2    0.656317    0.242488    0.338320    11.00000   -1.50000
N4    3    0.425408    0.314331    0.466810    11.00000    0.03267    0.04451 =
         0.05840   -0.02019    0.00242   -0.01746
```

```
H4    2    0.482648    0.371071    0.462616    11.00000   -1.50000
N5    3    0.278621    0.351426    0.534765    11.00000    0.03687    0.04239 =
         0.05122   -0.01673    0.00231   -0.01984
H5    2    0.222695    0.295053    0.533819    11.00000   -1.50000
O7    4    0.312371    0.555904    0.601081    11.00000    0.04158    0.04303 =
         0.05543   -0.01334   -0.00348   -0.02200
O9    4   -0.007174    0.399621    0.625838    11.00000    0.03707    0.05060 =
         0.05392   -0.02206    0.00387   -0.02328
S3    5    0.331603    0.115652    0.365485    11.00000    0.04610    0.05198 =
         0.07662   -0.02958    0.00384   -0.02643
S27   5    0.924709   -0.213359   -0.096378    11.00000    0.05324    0.06282 =
         0.05826   -0.02645   -0.00229   -0.01510
CL32  6   -0.095956    0.158411    0.550785    11.00000    0.04399    0.04099 =
         0.07394   -0.02546   -0.00467   -0.00804
CL34  6   -0.621521    0.342918    0.875628    11.00000    0.03798    0.06534 =
         0.06777    0.00064    0.00097   -0.02086
HKLF    4

REM  SIR92 run in space group P -1
REM R1 =  0.0391 for    3088 Fo > 4sig(Fo)  and  0.0564 for all    4067 data
REM    236 parameters refined using      0 restraints

END

WGHT      0.0426      0.1831

REM Highest difference peak  0.256,  deepest hole -0.300,  1-sigma level  0.047
Q1    1  -0.1912  0.4331  0.7382  11.00000  0.05    0.26
Q2    1   0.8989 -0.2832 -0.0368  11.00000  0.05    0.25
;
```

PK7

```
data_shelx

_audit_creation_method            'SHELXL-2014/7'
_shelx_SHELXL_version_number      '2014/7'
_chemical_name_systematic         ?
_chemical_name_common             ?
_chemical_melting_point           ?
_chemical_formula_moiety          'C15 H12 Cl2 I N3 O2 S'
_chemical_formula_sum             'C15 H12 Cl2 I N3 O2 S'
_chemical_formula_weight          496.14

loop_
 _atom_type_symbol
 _atom_type_description
 _atom_type_scat_dispersion_real
 _atom_type_scat_dispersion_imag
 _atom_type_scat_source
 'C'  'C'   0.0033   0.0016
 'International Tables Vol C Tables 4.2.6.8 and 6.1.1.4'
```

'H' 'H' 0.0000 0.0000
'International Tables Vol C Tables 4.2.6.8 and 6.1.1.4'
'N' 'N' 0.0061 0.0033
'International Tables Vol C Tables 4.2.6.8 and 6.1.1.4'
'O' 'O' 0.0106 0.0060
'International Tables Vol C Tables 4.2.6.8 and 6.1.1.4'
'S' 'S' 0.1246 0.1234
'International Tables Vol C Tables 4.2.6.8 and 6.1.1.4'
'Cl' 'Cl' 0.1484 0.1585
'International Tables Vol C Tables 4.2.6.8 and 6.1.1.4'
'I' 'I' -0.4742 1.8119
'International Tables Vol C Tables 4.2.6.8 and 6.1.1.4'

_space_group_crystal_system monoclinic
_space_group_IT_number 15
_space_group_name_H-M_alt 'C 2/c'
_space_group_name_Hall '-C 2yc'

_shelx_space_group_comment
;
The symmetry employed for this shelxl refinement is uniquely defined
by the following loop, which should always be used as a source of
symmetry information in preference to the above space-group names.
They are only intended as comments.
;

loop_
_space_group_symop_operation_xyz
'x, y, z'
'-x, y, -z+1/2'
'x+1/2, y+1/2, z'
'-x+1/2, y+1/2, -z+1/2'
'-x, -y, -z'
'x, -y, z-1/2'
'-x+1/2, -y+1/2, -z'
'x+1/2, -y+1/2, z-1/2'

_cell_length_a 14.7517(12)
_cell_length_b 11.2308(13)
_cell_length_c 22.628(3)
_cell_angle_alpha 90
_cell_angle_beta 103.123(10)
_cell_angle_gamma 90
_cell_volume 3651.0(7)
_cell_formula_units_Z 8
_cell_measurement_temperature 296(2)
_cell_measurement_reflns_used 2790
_cell_measurement_theta_min 2.2900
_cell_measurement_theta_max 28.9880

_exptl_crystal_description block
_exptl_crystal_colour yellow
_exptl_crystal_density_meas ?

_exptl_crystal_density_method ?
_exptl_crystal_density_diffrn 1.805
_exptl_crystal_F_000 1936
_exptl_transmission_factor_min ?
_exptl_transmission_factor_max ?
_exptl_crystal_size_max 0.50
_exptl_crystal_size_mid 0.40
_exptl_crystal_size_min 0.40
_exptl_absorpt_coefficient_mu 2.174
_shelx_estimated_absorpt_T_min ?
_shelx_estimated_absorpt_T_max ?
_exptl_absorpt_correction_T_min 0.52595
_exptl_absorpt_correction_T_max 1.00000
_exptl_absorpt_correction_type 'multi-scan'
_exptl_absorpt_process_details
;
CrysAlisPro, Agilent Technologies,
Version 1.171.37.35h (release 09-02-2015 CrysAlis171 .NET)
(compiled Feb 9 2015,16:26:32)
Empirical absorption correction using spherical harmonics,
implemented in SCALE3 ABSPACK scaling algorithm.
;
_exptl_absorpt_special_details ?
_diffrn_ambient_temperature 296(2)
_diffrn_radiation_wavelength 0.71073
_diffrn_radiation_type MoK\a
_diffrn_source ?
_diffrn_measurement_device_type 'KM4 CCD four-circle diffractometer'
_diffrn_measurement_method ?
_diffrn_detector_area_resol_mean ?
_diffrn_reflns_number 8148
_diffrn_reflns_av_unetI/netI 0.0519
_diffrn_reflns_av_R_equivalents 0.0419
_diffrn_reflns_limit_h_min -18
_diffrn_reflns_limit_h_max 19
_diffrn_reflns_limit_k_min -14
_diffrn_reflns_limit_k_max 14
_diffrn_reflns_limit_l_min -30
_diffrn_reflns_limit_l_max 28
_diffrn_reflns_theta_min 2.302
_diffrn_reflns_theta_max 29.083
_diffrn_reflns_theta_full 25.242
_diffrn_measured_fraction_theta_max 0.845
_diffrn_measured_fraction_theta_full 0.994
_diffrn_reflns_Laue_measured_fraction_max 0.845
_diffrn_reflns_Laue_measured_fraction_full 0.994
_diffrn_reflns_point_group_measured_fraction_max 0.845
_diffrn_reflns_point_group_measured_fraction_full 0.994
_reflns_number_total 4142
_reflns_number_gt 3086
_reflns_threshold_expression 'I > 2\s(I)'
_reflns_Friedel_coverage 0.000
_reflns_Friedel_fraction_max .

_reflns_Friedel_fraction_full .

_reflns_special_details
;
Reflections were merged by SHELXL according to the crystal
class for the calculation of statistics and refinement.

_reflns_Friedel_fraction is defined as the number of unique
Friedel pairs measured divided by the number that would be
possible theoretically, ignoring centric projections and
systematic absences.
;

_computing_data_collection
;
CrysAlisPro, Agilent Technologies,
Version 1.171.37.35h (release 09-02-2015 CrysAlis171 .NET)
(compiled Feb 9 2015,16:26:32)
;
_computing_cell_refinement
;
CrysAlisPro, Agilent Technologies,
Version 1.171.37.35h (release 09-02-2015 CrysAlis171 .NET)
(compiled Feb 9 2015,16:26:32)
;
_computing_data_reduction
;
CrysAlisPro, Agilent Technologies,
Version 1.171.37.35h (release 09-02-2015 CrysAlis171 .NET)
(compiled Feb 9 2015,16:26:32)
;
_computing_structure_solution ?
_computing_structure_refinement 'SHELXL-2014/7 (Sheldrick, 2014)'
_computing_molecular_graphics ?
_computing_publication_material ?
_refine_special_details ?
_refine_ls_structure_factor_coef Fsqd
_refine_ls_matrix_type full
_refine_ls_weighting_scheme calc
_refine_ls_weighting_details
'w=1/[\s^2^(Fo^2^)+(0.0801P)^2^+0.0750P] where P=(Fo^2^+2Fc^2^)/3'
_atom_sites_solution_primary difmap
_atom_sites_solution_secondary difmap
_atom_sites_solution_hydrogens difmap
_refine_ls_hydrogen_treatment mixed
_refine_ls_extinction_method none
_refine_ls_extinction_coef .
_refine_ls_number_reflns 4142
_refine_ls_number_parameters 236
_refine_ls_number_restraints 0
_refine_ls_R_factor_all 0.0740
_refine_ls_R_factor_gt 0.0543
_refine_ls_wR_factor_ref 0.1562

_refine_ls_wR_factor_gt 0.1341
_refine_ls_goodness_of_fit_ref 1.058
_refine_ls_restrained_S_all 1.058
_refine_ls_shift/su_max 0.001
_refine_ls_shift/su_mean 0.000

loop_
_atom_site_label
_atom_site_type_symbol
_atom_site_fract_x
_atom_site_fract_y
_atom_site_fract_z
_atom_site_U_iso_or_equiv
_atom_site_adp_type
_atom_site_occupancy
_atom_site_site_symmetry_order
_atom_site_calc_flag
_atom_site_refinement_flags_posn
_atom_site_refinement_flags_adp
_atom_site_refinement_flags_occupancy
_atom_site_disorder_assembly
_atom_site_disorder_group
C2 C 0.6621(2) 0.6234(4) 0.94301(15) 0.0397(9) Uani 1 1 d . . . . .
C21 C 0.6125(2) 0.7735(4) 0.86253(17) 0.0417(9) Uani 1 1 d . . . . .
C22 C 0.5988(3) 0.8934(5) 0.8656(2) 0.0595(12) Uani 1 1 d . . . . .
H22 H 0.5832 0.9265 0.8997 0.089 Uiso 1 1 calc R U . . .
C23 C 0.6084(4) 0.9667(5) 0.8168(3) 0.0687(14) Uani 1 1 d . . . . .
H23 H 0.5989 1.0483 0.8185 0.103 Uiso 1 1 calc R U . . .
C24 C 0.6312(3) 0.9188(5) 0.7679(2) 0.0551(12) Uani 1 1 d . . . . .
C25 C 0.6469(4) 0.7989(5) 0.7648(2) 0.0691(14) Uani 1 1 d . . . . .
H25 H 0.6639 0.7668 0.7310 0.104 Uiso 1 1 calc R U . . .
C26 C 0.6374(4) 0.7261(4) 0.8120(2) 0.0614(12) Uani 1 1 d . . . . .
H26 H 0.6477 0.6446 0.8099 0.092 Uiso 1 1 calc R U . . .
C33 C 1.0375(2) 0.2009(4) 1.10739(16) 0.0403(8) Uani 1 1 d . . . . .
H33 H 1.0939 0.2135 1.0966 0.060 Uiso 1 1 calc R U . . .
C34 C 1.0302(3) 0.1167(4) 1.15065(17) 0.0470(10) Uani 1 1 d . . . . .
C6 C 0.6480(2) 0.3933(4) 1.04844(15) 0.0392(9) Uani 1 1 d . . . . .
C8 C 0.7149(2) 0.3041(5) 1.08411(17) 0.0481(10) Uani 1 1 d . . . . .
H8A H 0.7226 0.3183 1.1273 0.072 Uiso 1 1 calc R U . . .
H8B H 0.6912 0.2240 1.0752 0.072 Uiso 1 1 calc R U . . .
C31 C 0.8744(2) 0.2474(4) 1.09669(16) 0.0389(9) Uani 1 1 d . . . . .
C32 C 0.9600(2) 0.2657(4) 1.08056(15) 0.0379(8) Uani 1 1 d . . . . .
C35 C 0.9477(3) 0.0976(5) 1.16741(19) 0.0525(11) Uani 1 1 d . . . . .
H35 H 0.9443 0.0415 1.1971 0.079 Uiso 1 1 calc R U . . .
C36 C 0.8697(3) 0.1621(4) 1.13993(17) 0.0486(10) Uani 1 1 d . . . . .
H36 H 0.8134 0.1479 1.1506 0.073 Uiso 1 1 calc R U . . .
N1 N 0.5985(2) 0.6977(4) 0.91000(15) 0.0507(9) Uani 1 1 d . . . . .
H1 H 0.546(4) 0.676(5) 0.916(2) 0.076 Uiso 1 1 d . U . . .
N4 N 0.6277(2) 0.5555(4) 0.98179(15) 0.0487(9) Uani 1 1 d . . . . .
H4 H 0.562(4) 0.554(5) 0.981(2) 0.073 Uiso 1 1 d . U . . .
N5 N 0.6837(2) 0.4714(4) 1.01724(16) 0.0462(9) Uani 1 1 d . . . . .
H5 H 0.738(4) 0.482(5) 1.010(2) 0.069 Uiso 1 1 d . U . . .
O7 O 0.56486(16) 0.3906(3) 1.04965(12) 0.0485(7) Uani 1 1 d . . . . .

O9 O 0.80247(16) 0.3170(3) 1.06739(12) 0.0504(7) Uani 1 1 d . . . . .
S3 S 0.77450(6) 0.61598(12) 0.94058(5) 0.0507(3) Uani 1 1 d . . . . .
Cl32 Cl 0.96720(7) 0.37241(10) 1.02692(5) 0.0534(3) Uani 1 1 d . . . . .
Cl34 Cl 1.12884(9) 0.03691(15) 1.18657(6) 0.0706(4) Uani 1 1 d . . . . .
I24A I 0.65332(16) 1.0147(3) 0.69073(10) 0.0620(7) Uani 0.54(2) 1 d . . P A 1
I24B I 0.6481(3) 1.0470(12) 0.7059(6) 0.107(2) Uani 0.46(2) 1 d . . P A 2

loop_
 _atom_site_aniso_label
 _atom_site_aniso_U_11
 _atom_site_aniso_U_22
 _atom_site_aniso_U_33
 _atom_site_aniso_U_23
 _atom_site_aniso_U_13
 _atom_site_aniso_U_12
C2 0.0311(16) 0.052(3) 0.0400(17) 0.0043(16) 0.0165(14) -0.0010(17)
C21 0.0326(17) 0.048(2) 0.0478(19) 0.0110(18) 0.0161(15) 0.0063(18)
C22 0.063(3) 0.056(3) 0.061(3) -0.007(2) 0.018(2) 0.010(3)
C23 0.068(3) 0.044(3) 0.093(4) 0.017(3) 0.015(3) 0.009(3)
C24 0.0364(19) 0.061(3) 0.067(3) 0.028(2) 0.0106(19) 0.002(2)
C25 0.085(3) 0.074(4) 0.061(3) 0.011(3) 0.042(2) 0.004(3)
C26 0.083(3) 0.044(3) 0.070(3) 0.008(2) 0.045(2) 0.003(3)
C33 0.0260(15) 0.045(2) 0.0508(19) -0.0036(17) 0.0106(14) 0.0012(16)
C34 0.0341(17) 0.054(3) 0.049(2) -0.0020(19) 0.0019(16) 0.0049(19)
C6 0.0274(15) 0.053(3) 0.0398(17) 0.0036(16) 0.0129(13) 0.0024(17)
C8 0.0263(16) 0.066(3) 0.055(2) 0.017(2) 0.0166(15) 0.0074(19)
C31 0.0261(16) 0.047(2) 0.0439(18) 0.0007(16) 0.0072(14) 0.0014(16)
C32 0.0338(16) 0.039(2) 0.0426(18) -0.0025(15) 0.0131(14) -0.0030(17)
C35 0.042(2) 0.056(3) 0.057(2) 0.013(2) 0.0050(18) -0.004(2)
C36 0.0312(17) 0.062(3) 0.055(2) 0.009(2) 0.0132(16) -0.0036(19)
N1 0.0346(15) 0.066(3) 0.0594(19) 0.0197(18) 0.0265(15) 0.0102(18)
N4 0.0278(14) 0.067(3) 0.0547(19) 0.0217(18) 0.0162(14) 0.0078(17)
N5 0.0252(14) 0.061(3) 0.0547(18) 0.0186(17) 0.0144(14) 0.0041(16)
O7 0.0269(12) 0.065(2) 0.0573(15) 0.0161(14) 0.0169(11) 0.0034(13)
O9 0.0292(12) 0.065(2) 0.0614(16) 0.0226(15) 0.0200(11) 0.0099(14)
S3 0.0295(4) 0.0693(8) 0.0588(6) 0.0114(5) 0.0219(4) 0.0012(5)
Cl32 0.0468(5) 0.0518(7) 0.0685(6) 0.0147(5) 0.0275(5) 0.0005(5)
Cl34 0.0473(6) 0.0875(11) 0.0718(7) 0.0112(7) 0.0027(5) 0.0246(7)
I24A 0.0491(6) 0.0737(9) 0.0659(11) 0.0328(8) 0.0188(4) 0.0093(6)
I24B 0.0662(10) 0.118(4) 0.129(3) 0.087(3) 0.0068(14) -0.0113(14)

_geom_special_details
;
 All esds (except the esd in the dihedral angle between two l.s. planes)
 are estimated using the full covariance matrix. The cell esds are taken
 into account individually in the estimation of esds in distances, angles
 and torsion angles; correlations between esds in cell parameters are only
 used when they are defined by crystal symmetry. An approximate (isotropic)
 treatment of cell esds is used for estimating esds involving l.s. planes.
;

loop_
 _geom_bond_atom_site_label_1

```
 _geom_bond_atom_site_label_2
 _geom_bond_distance
 _geom_bond_site_symmetry_2
 _geom_bond_publ_flag
C2 N4 1.346(5) . ?
C2 N1 1.347(5) . ?
C2 S3 1.674(3) . ?
C21 C22 1.366(6) . ?
C21 C26 1.384(6) . ?
C21 N1 1.423(5) . ?
C22 C23 1.410(7) . ?
C22 H22 0.9300 . ?
C23 C24 1.340(8) . ?
C23 H23 0.9300 . ?
C24 C25 1.370(8) . ?
C24 I24B 2.065(5) . ?
C24 I24A 2.139(5) . ?
C25 C26 1.376(6) . ?
C25 H25 0.9300 . ?
C26 H26 0.9300 . ?
C33 C32 1.375(5) . ?
C33 C34 1.383(6) . ?
C33 H33 0.9300 . ?
C34 C35 1.372(6) . ?
C34 Cl34 1.745(4) . ?
C6 O7 1.234(4) . ?
C6 N5 1.309(5) . ?
C6 C8 1.506(5) . ?
C8 O9 1.433(4) . ?
C8 H8A 0.9700 . ?
C8 H8B 0.9700 . ?
C31 O9 1.363(4) . ?
C31 C36 1.383(6) . ?
C31 C32 1.406(5) . ?
C32 Cl32 1.726(4) . ?
C35 C36 1.383(6) . ?
C35 H35 0.9300 . ?
C36 H36 0.9300 . ?
N1 H1 0.85(6) . ?
N4 N5 1.385(5) . ?
N4 H4 0.97(6) . ?
N5 H5 0.85(6) . ?

loop_
 _geom_angle_atom_site_label_1
 _geom_angle_atom_site_label_2
 _geom_angle_atom_site_label_3
 _geom_angle
 _geom_angle_site_symmetry_1
 _geom_angle_site_symmetry_3
 _geom_angle_publ_flag
N4 C2 N1 113.3(3) . . ?
N4 C2 S3 121.1(3) . . ?
```

N1 C2 S3 125.6(3) . . ?
C22 C21 C26 119.5(4) . . ?
C22 C21 N1 120.2(4) . . ?
C26 C21 N1 120.3(4) . . ?
C21 C22 C23 119.5(4) . . ?
C21 C22 H22 120.3 . . ?
C23 C22 H22 120.3 . . ?
C24 C23 C22 120.1(5) . . ?
C24 C23 H23 120.0 . . ?
C22 C23 H23 120.0 . . ?
C23 C24 C25 120.9(4) . . ?
C23 C24 I24B 111.9(7) . . ?
C25 C24 I24B 127.1(7) . . ?
C23 C24 I24A 125.8(4) . . ?
C25 C24 I24A 113.3(4) . . ?
C24 C25 C26 119.8(5) . . ?
C24 C25 H25 120.1 . . ?
C26 C25 H25 120.1 . . ?
C25 C26 C21 120.3(5) . . ?
C25 C26 H26 119.9 . . ?
C21 C26 H26 119.9 . . ?
C32 C33 C34 119.0(3) . . ?
C32 C33 H33 120.5 . . ?
C34 C33 H33 120.5 . . ?
C35 C34 C33 121.3(4) . . ?
C35 C34 Cl34 119.3(3) . . ?
C33 C34 Cl34 119.4(3) . . ?
O7 C6 N5 123.5(4) . . ?
O7 C6 C8 120.4(3) . . ?
N5 C6 C8 116.1(3) . . ?
O9 C8 C6 108.2(3) . . ?
O9 C8 H8A 110.1 . . ?
C6 C8 H8A 110.1 . . ?
O9 C8 H8B 110.1 . . ?
C6 C8 H8B 110.1 . . ?
H8A C8 H8B 108.4 . . ?
O9 C31 C36 125.6(3) . . ?
O9 C31 C32 115.8(3) . . ?
C36 C31 C32 118.5(3) . . ?
C33 C32 C31 120.9(3) . . ?
C33 C32 Cl32 120.0(3) . . ?
C31 C32 Cl32 119.1(3) . . ?
C34 C35 C36 119.6(4) . . ?
C34 C35 H35 120.2 . . ?
C36 C35 H35 120.2 . . ?
C35 C36 C31 120.7(4) . . ?
C35 C36 H36 119.7 . . ?
C31 C36 H36 119.7 . . ?
C2 N1 C21 126.0(3) . . ?
C2 N1 H1 107(4) . . ?
C21 N1 H1 125(3) . . ?
C2 N4 N5 120.0(3) . . ?
C2 N4 H4 121(3) . . ?

N5 N4 H4 118(3) . . ?
C6 N5 N4 120.6(3) . . ?
C6 N5 H5 134(4) . . ?
N4 N5 H5 105(4) . . ?
C31 O9 C8 117.6(3) . . ?

loop_
 _geom_torsion_atom_site_label_1
 _geom_torsion_atom_site_label_2
 _geom_torsion_atom_site_label_3
 _geom_torsion_atom_site_label_4
 _geom_torsion
 _geom_torsion_site_symmetry_1
 _geom_torsion_site_symmetry_2
 _geom_torsion_site_symmetry_3
 _geom_torsion_site_symmetry_4
 _geom_torsion_publ_flag
C26 C21 C22 C23 1.2(7) . . . . ?
N1 C21 C22 C23 -176.8(4) . . . . ?
C21 C22 C23 C24 -0.3(8) . . . . ?
C22 C23 C24 C25 -1.0(8) . . . . ?
C22 C23 C24 I24B -176.6(4) . . . . ?
C22 C23 C24 I24A -177.9(4) . . . . ?
C23 C24 C25 C26 1.3(8) . . . . ?
I24B C24 C25 C26 176.2(4) . . . . ?
I24A C24 C25 C26 178.6(4) . . . . ?
C24 C25 C26 C21 -0.3(8) . . . . ?
C22 C21 C26 C25 -0.9(7) . . . . ?
N1 C21 C26 C25 177.1(4) . . . . ?
C32 C33 C34 C35 -0.5(6) . . . . ?
C32 C33 C34 Cl34 -178.2(3) . . . . ?
O7 C6 C8 O9 171.6(4) . . . . ?
N5 C6 C8 O9 -8.5(5) . . . . ?
C34 C33 C32 C31 0.2(6) . . . . ?
C34 C33 C32 Cl32 179.4(3) . . . . ?
O9 C31 C32 C33 179.7(3) . . . . ?
C36 C31 C32 C33 -0.4(6) . . . . ?
O9 C31 C32 Cl32 0.4(5) . . . . ?
C36 C31 C32 Cl32 -179.7(3) . . . . ?
C33 C34 C35 C36 1.1(7) . . . . ?
Cl34 C34 C35 C36 178.8(4) . . . . ?
C34 C35 C36 C31 -1.4(7) . . . . ?
O9 C31 C36 C35 -179.1(4) . . . . ?
C32 C31 C36 C35 1.1(6) . . . . ?
N4 C2 N1 C21 -175.2(4) . . . . ?
S3 C2 N1 C21 7.5(7) . . . . ?
C22 C21 N1 C2 -122.9(5) . . . . ?
C26 C21 N1 C2 59.2(6) . . . . ?
N1 C2 N4 N5 177.6(4) . . . . ?
S3 C2 N4 N5 -5.0(6) . . . . ?
O7 C6 N5 N4 -1.6(6) . . . . ?
C8 C6 N5 N4 178.5(4) . . . . ?
C2 N4 N5 C6 -170.0(4) . . . . ?

C36 C31 O9 C8 2.3(6) . . . . ?
C32 C31 O9 C8 -177.8(4) . . . . ?
C6 C8 O9 C31 177.2(3) . . . . ?

```
_refine_diff_density_max    0.892
_refine_diff_density_min   -0.852
_refine_diff_density_rms    0.125

_shelx_res_file
;

    shelx.res created by SHELXL-2014/7


TITL PK7_20190409 in C2/c
CELL  0.71073  14.7517  11.2308  22.6284   90.000  103.123   90.000
ZERR    8.00   0.0012   0.0013   0.0026    0.000    0.010    0.000
LATT   7
SYMM  - X,   Y, 1/2 - Z
SFAC  C  H  N  O  S  CL  I
UNIT  120 96 24 16 8 16 8
MERG   2
FMAP   2
ACTA
OMIT   -4   4   2
OMIT       0   0   2
OMIT      -3   7   3
OMIT      -7   3   4
OMIT      -7   3   5
OMIT     -13   1   7
OMIT      -9   5   7
OMIT      -5  11  14
OMIT      -5   1   3
BOND   $H
CONF
L.S.  50
WGHT    0.080100    0.075000
FVAR       1.07902   0.54061
C2    1    0.662060    0.623362    0.943008    11.00000    0.03114    0.05200 =
         0.04000    0.00430    0.01648   -0.00098
C21   1    0.612539    0.773506    0.862525    11.00000    0.03256    0.04805 =
         0.04783    0.01104    0.01607    0.00629
C22   1    0.598771    0.893409    0.865623    11.00000    0.06266    0.05628 =
         0.06138   -0.00675    0.01796    0.00952
AFIX  43
H22   2    0.583183    0.926477    0.899673    11.00000   -1.50000
AFIX   0
C23   1    0.608377    0.966682    0.816790    11.00000    0.06808    0.04361 =
         0.09282    0.01727    0.01490    0.00861
AFIX  43
H23   2    0.598864    1.048346    0.818524    11.00000   -1.50000
AFIX   0
C24   1    0.631199    0.918766    0.767886    11.00000    0.03640    0.06110 =
```

0.06718 0.02762 0.01063 0.00182
C25 1 0.646878 0.798884 0.764836 11.00000 0.08475 0.07353 =
0.06123 0.01148 0.04180 0.00405
AFIX 43
H25 2 0.663878 0.766844 0.731024 11.00000 -1.50000
AFIX 0
C26 1 0.637382 0.726058 0.811982 11.00000 0.08282 0.04438 =
0.07015 0.00791 0.04461 0.00296
AFIX 43
H26 2 0.647698 0.644636 0.809876 11.00000 -1.50000
AFIX 0
C33 1 1.037471 0.200928 1.107388 11.00000 0.02595 0.04506 =
0.05080 -0.00357 0.01058 0.00123
AFIX 43
H33 2 1.093906 0.213550 1.096607 11.00000 -1.50000
AFIX 0
C34 1 1.030222 0.116716 1.150647 11.00000 0.03405 0.05419 =
0.04912 -0.00196 0.00195 0.00486

C6 1 0.648026 0.393323 1.048439 11.00000 0.02740 0.05282 =
0.03983 0.00357 0.01294 0.00241
C8 1 0.714927 0.304137 1.084113 11.00000 0.02633 0.06627 =
0.05516 0.01659 0.01656 0.00736
AFIX 23
H8A 2 0.722588 0.318267 1.127255 11.00000 -1.50000
H8B 2 0.691159 0.224040 1.075165 11.00000 -1.50000
AFIX 0
C31 1 0.874410 0.247419 1.096687 11.00000 0.02607 0.04651 =
0.04389 0.00074 0.00716 0.00137
C32 1 0.959964 0.265721 1.080555 11.00000 0.03375 0.03935 =
0.04264 -0.00246 0.01309 -0.00299
C35 1 0.947716 0.097554 1.167408 11.00000 0.04234 0.05560 =
0.05659 0.01335 0.00503 -0.00360
AFIX 43
H35 2 0.944290 0.041512 1.197080 11.00000 -1.50000
AFIX 0
C36 1 0.869657 0.162053 1.139931 11.00000 0.03115 0.06191 =
0.05450 0.00929 0.01321 -0.00359
AFIX 43
H36 2 0.813371 0.147878 1.150642 11.00000 -1.50000
AFIX 0
N1 3 0.598525 0.697653 0.910002 11.00000 0.03462 0.06561 =
0.05945 0.01972 0.02650 0.01017
H1 2 0.545961 0.676254 0.915677 11.00000 -1.50000
N4 3 0.627686 0.555476 0.981785 11.00000 0.02784 0.06674 =
0.05474 0.02170 0.01617 0.00782
H4 2 0.561529 0.554244 0.980802 11.00000 -1.50000
N5 3 0.683717 0.471382 1.017240 11.00000 0.02520 0.06130 =
0.05470 0.01862 0.01444 0.00414
H5 2 0.737623 0.481736 1.010440 11.00000 -1.50000
O7 4 0.564861 0.390592 1.049646 11.00000 0.02694 0.06462 =
0.05735 0.01610 0.01691 0.00343
O9 4 0.802474 0.317010 1.067393 11.00000 0.02918 0.06529 =

```
        0.06141    0.02263    0.01996    0.00992
S3    5    0.774498    0.615979    0.940579    11.00000    0.02954    0.06934 =
        0.05884    0.01143    0.02189    0.00119
CL32  6    0.967203    0.372407    1.026916    11.00000    0.04681    0.05184 =
        0.06853    0.01467    0.02746    0.00054
CL34  6    1.128839    0.036913    1.186565    11.00000    0.04729    0.08753 =
        0.07183    0.01119    0.00268    0.02462
PART 1 21
I24A  7    0.653325    1.014738    0.690730    21.00000    0.04908    0.07367 =
        0.06595    0.03282    0.01878    0.00934
PART 0
PART 2 -21
I24B  7    0.648128    1.047033    0.705873   -21.00000    0.06624    0.11809 =
        0.12936    0.08726    0.00682   -0.01126
PART 0
HKLF    4

REM  PK7_20190409 in C2/c
REM R1 =  0.0543 for    3086 Fo > 4sig(Fo)  and  0.0740 for all    4142 data
REM    236 parameters refined using      0 restraints

END

WGHT      0.0801      0.0750

REM Highest difference peak  0.892,  deepest hole -0.852,  1-sigma level  0.125
Q1    1   0.7077  1.0049  0.7392  11.00000  0.05    0.89
Q2    1   0.5975  1.0386  0.6498  11.00000  0.05    0.85
Q3    1   0.6129  1.0912  0.6725  11.00000  0.05    0.82
Q4    1   0.6972  0.9570  0.7233  11.00000  0.05    0.82
Q5    1   0.6046  0.9586  0.6682  11.00000  0.05    0.63
Q6    1   0.6928  1.1174  0.7371  11.00000  0.05    0.56
Q7    1   0.7311  1.0371  0.8292  11.00000  0.05    0.51
Q8    1   0.7379  0.6888  0.9218  11.00000  0.05    0.51
Q9    1   0.7125  1.0276  0.7996  11.00000  0.05    0.48
Q10   1   0.5823  1.0379  0.6006  11.00000  0.05    0.47
Q11   1   0.9974  0.2966  1.0444  11.00000  0.05    0.46
Q12   1   0.9356  0.2847  1.0088  11.00000  0.05    0.45
Q13   1   0.7626  0.4312  1.1432  11.00000  0.05    0.45
Q14   1   1.0829  0.1089  1.1656  11.00000  0.05    0.43
Q15   1   0.9856  0.4741  1.0370  11.00000  0.05    0.41
Q16   1   0.7755  0.7239  0.9377  11.00000  0.05    0.40
Q17   1   0.5524  1.0199  0.6920  11.00000  0.05    0.39
Q18   1   1.0852 -0.0088  1.1768  11.00000  0.05    0.37
Q19   1   1.0382  0.1837  1.1441  11.00000  0.05    0.37
Q20   1   0.7460  0.5369  0.9288  11.00000  0.05    0.37
;
```

^{1}H NMR for compound PK1

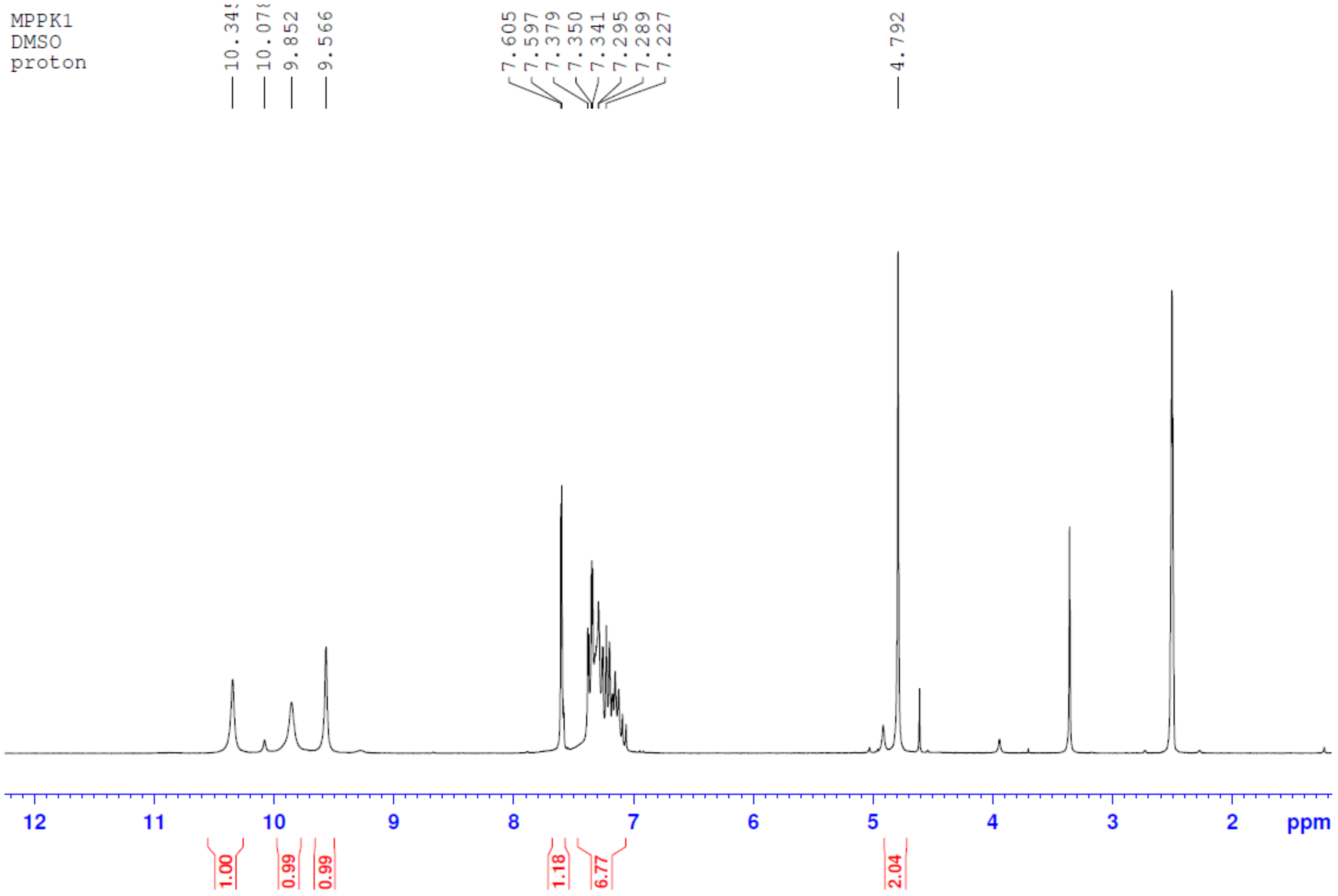

^{1}H NMR for compound PK2

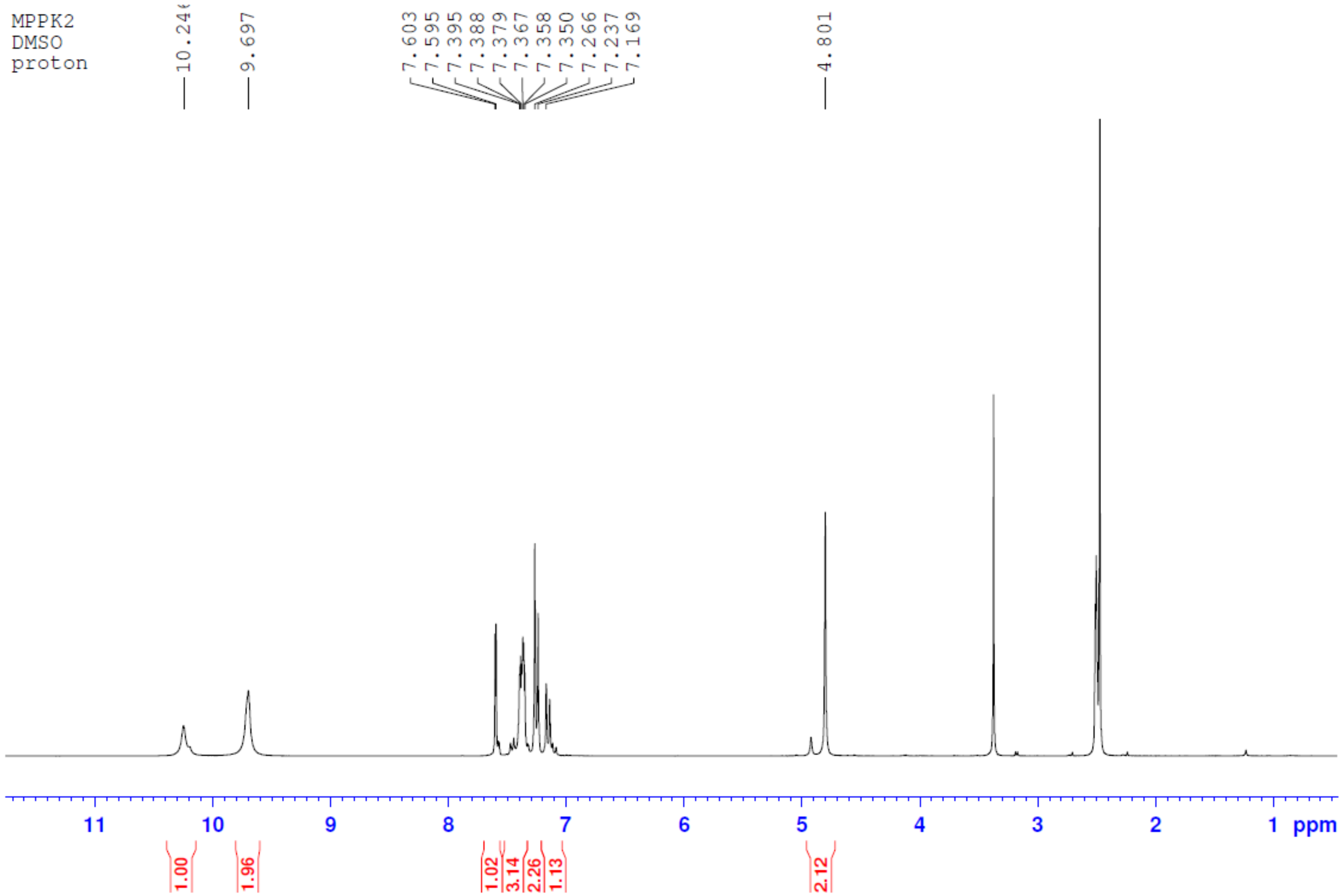

^{1}H NMR for compound PK3

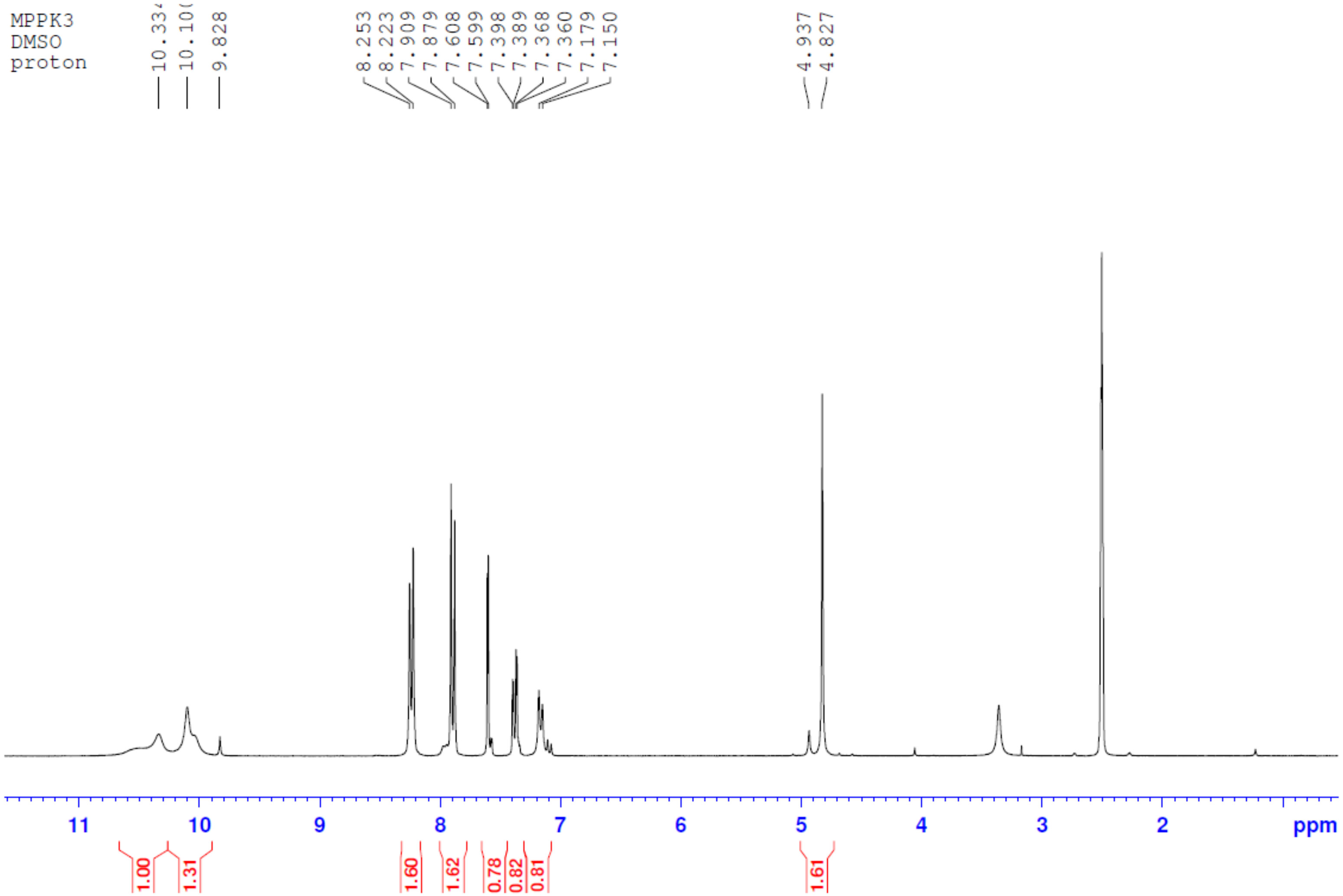

^{1}H NMR for compound PK4

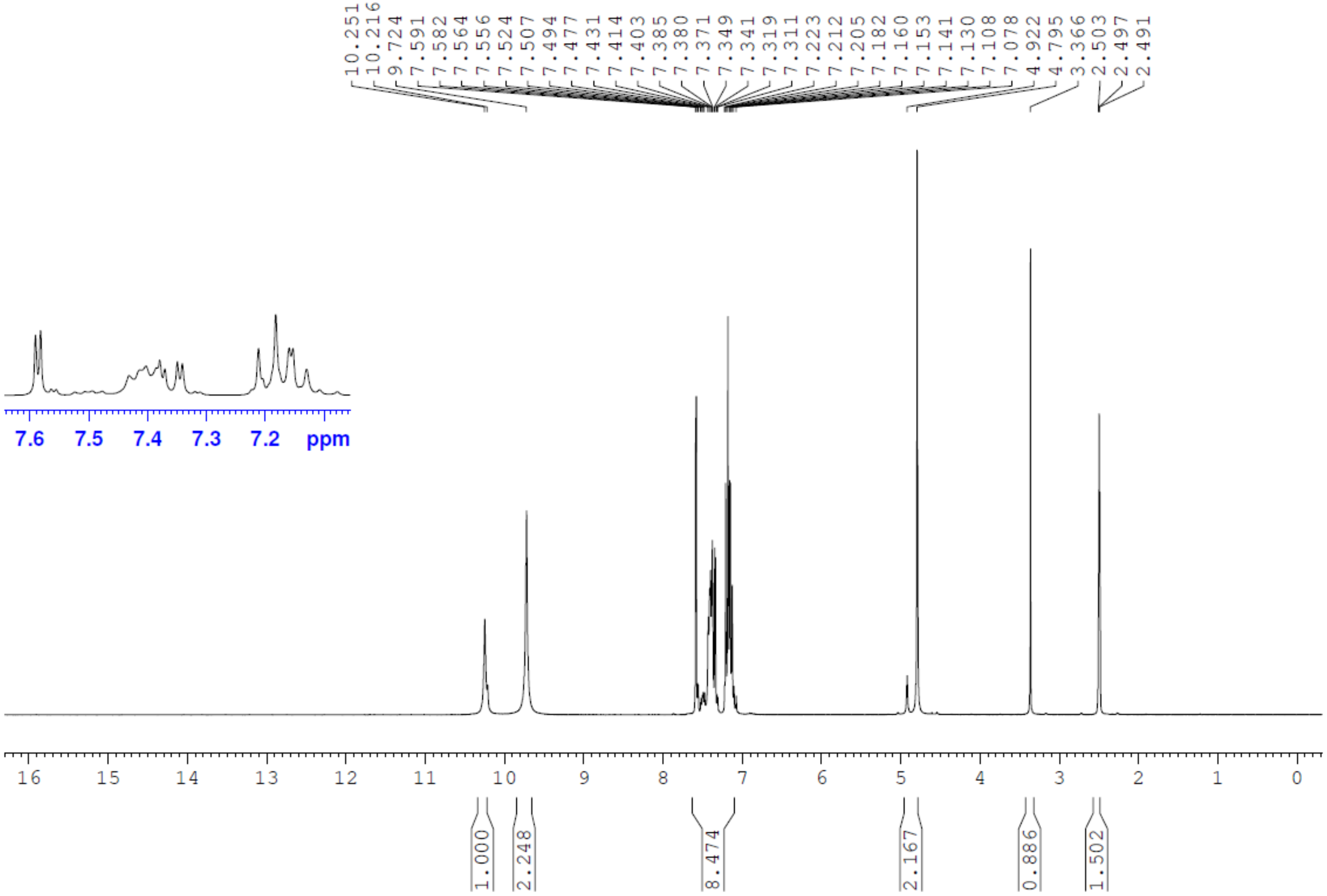

^{1}H NMR for compound PK5

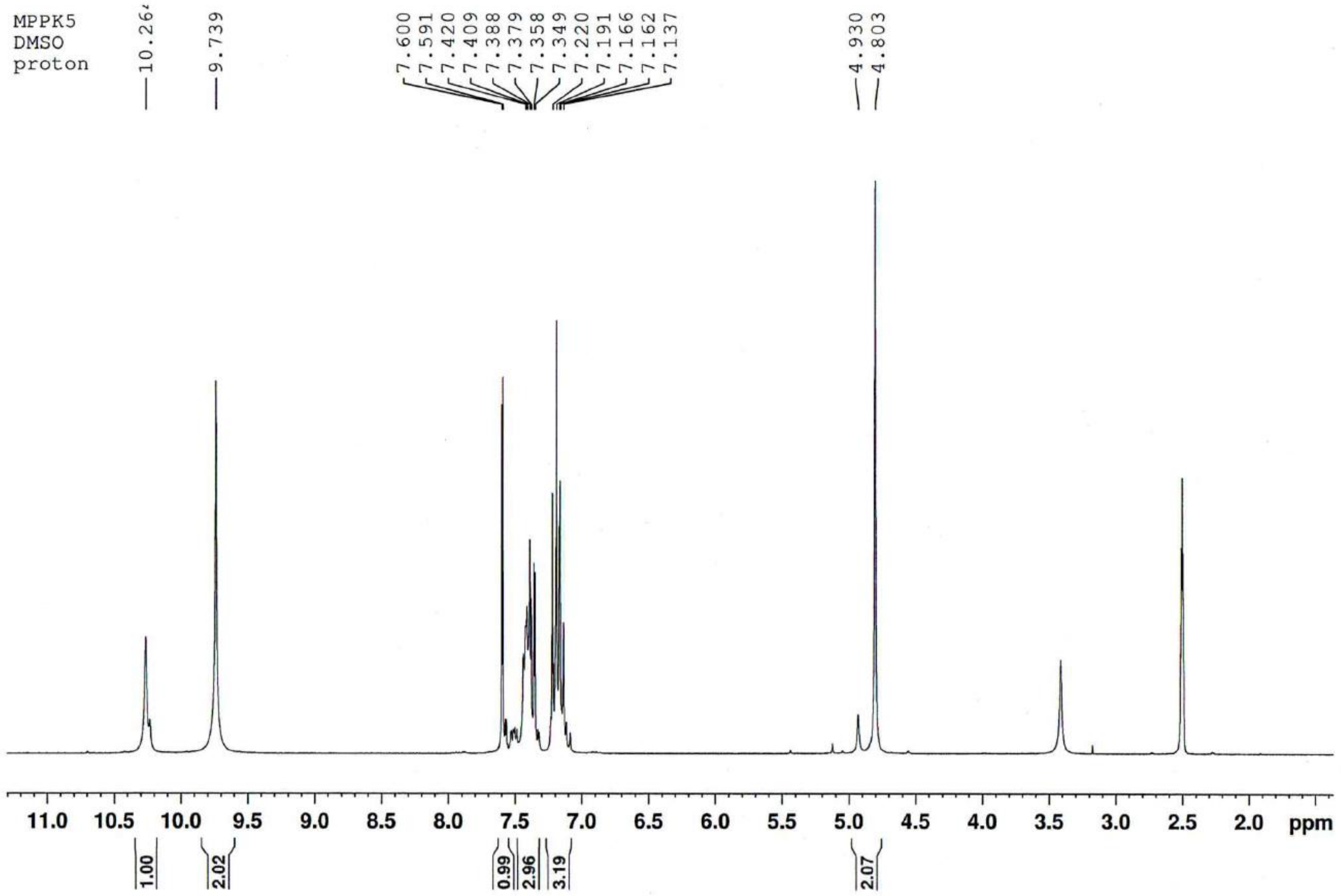

^{1}H NMR for compound PK6

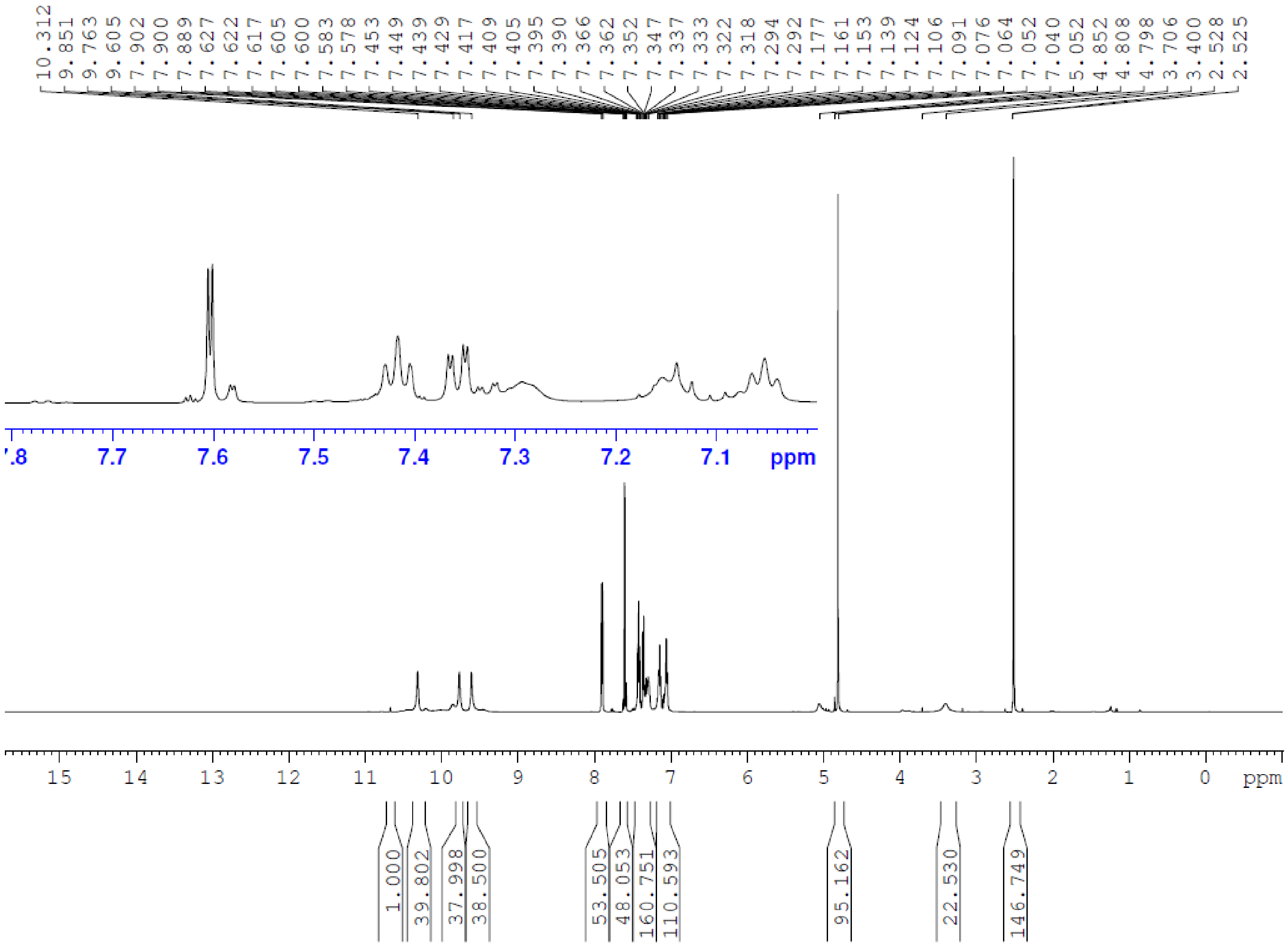

^{1}H NMR for compound PK7

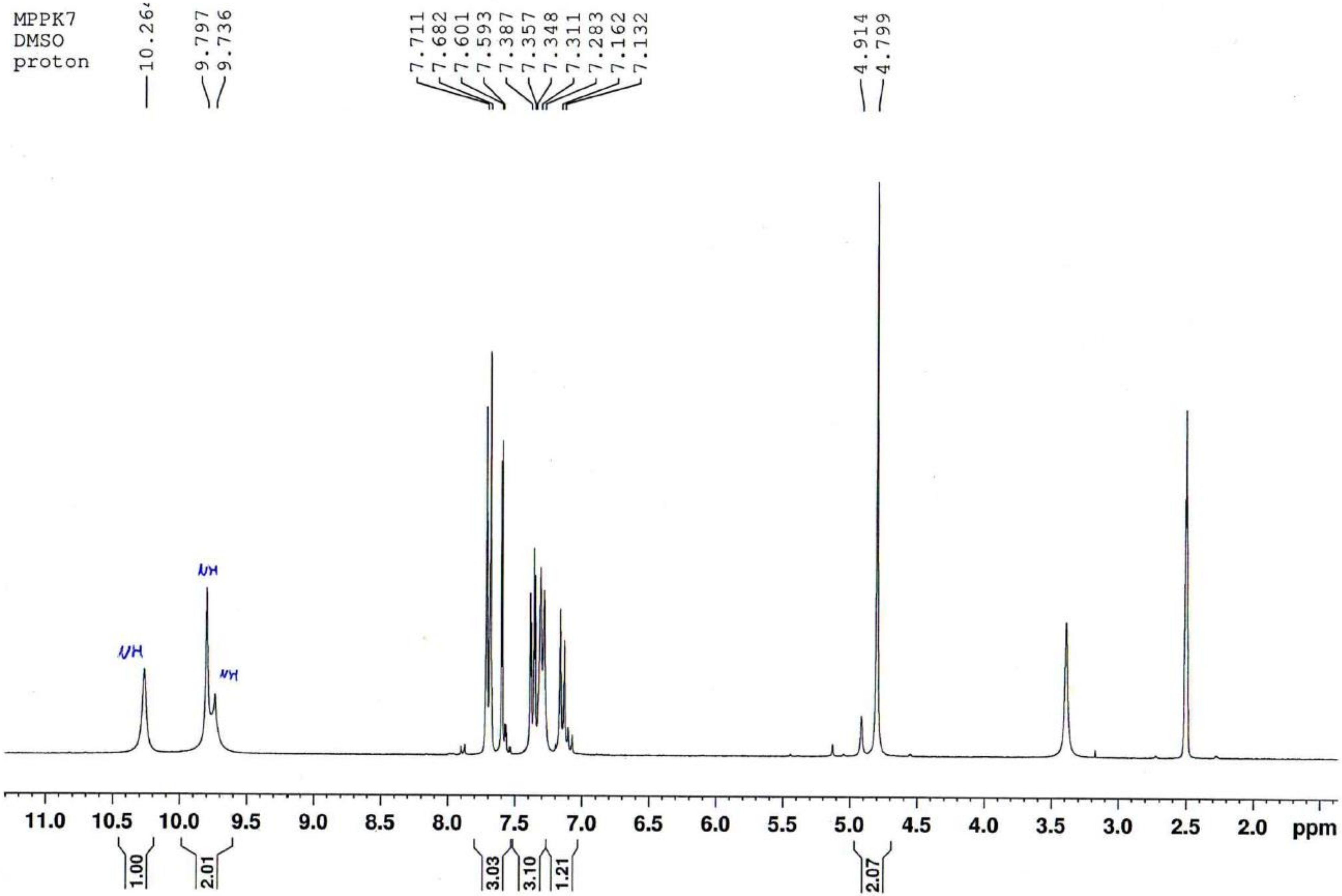

^{1}H NMR for compound PK8

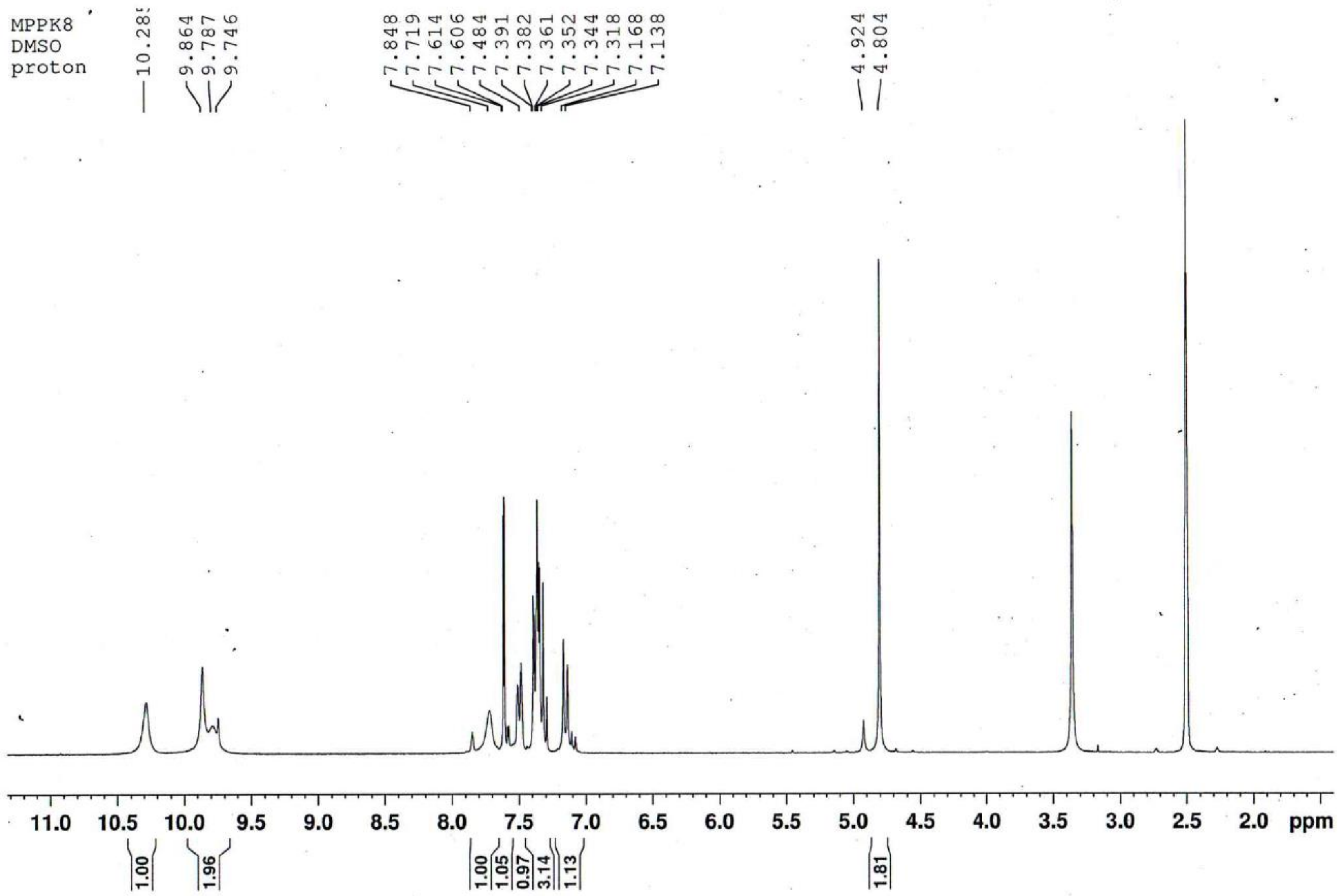

^{1}H NMR for compound PK10

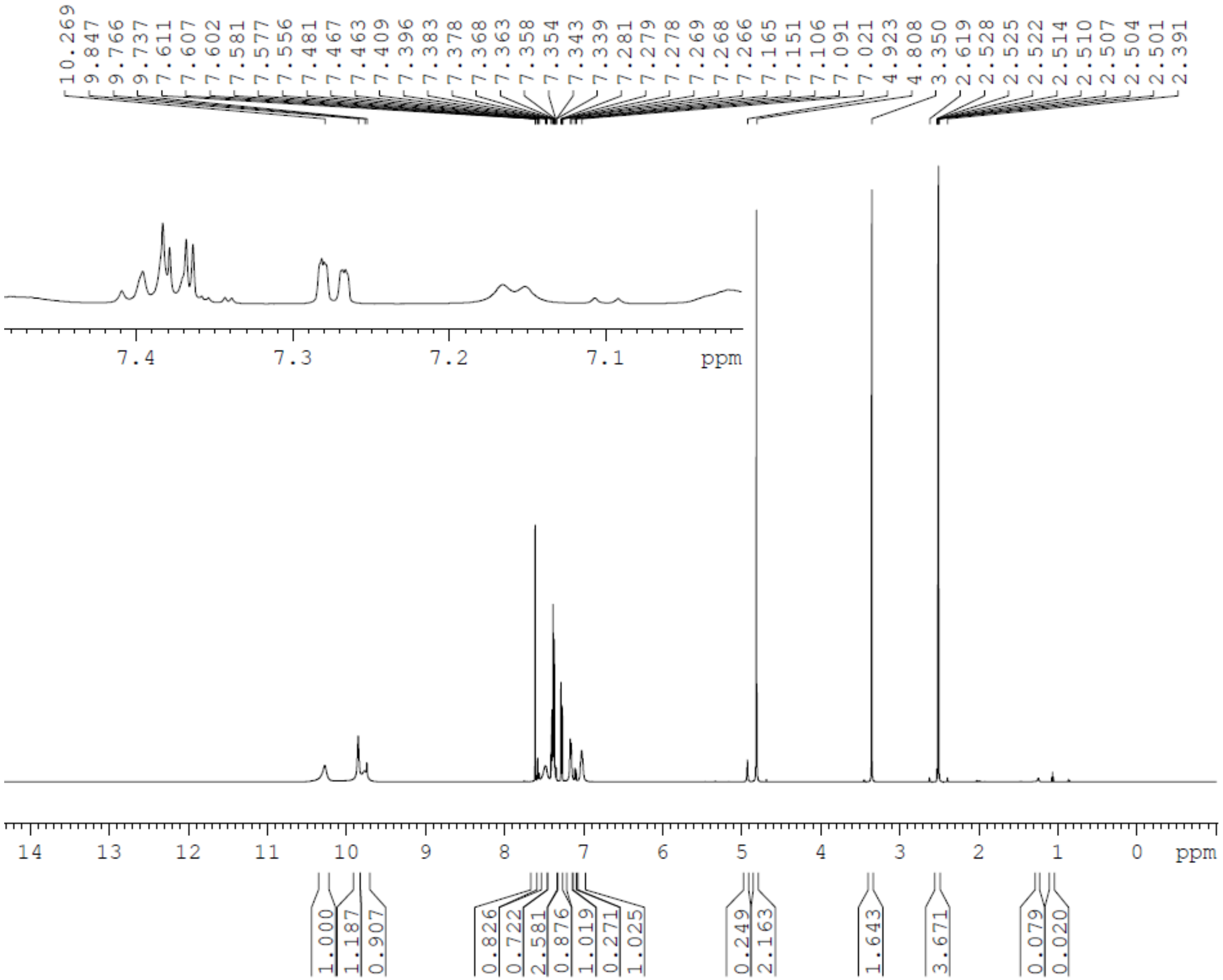

^{1}H NMR for compound PK11

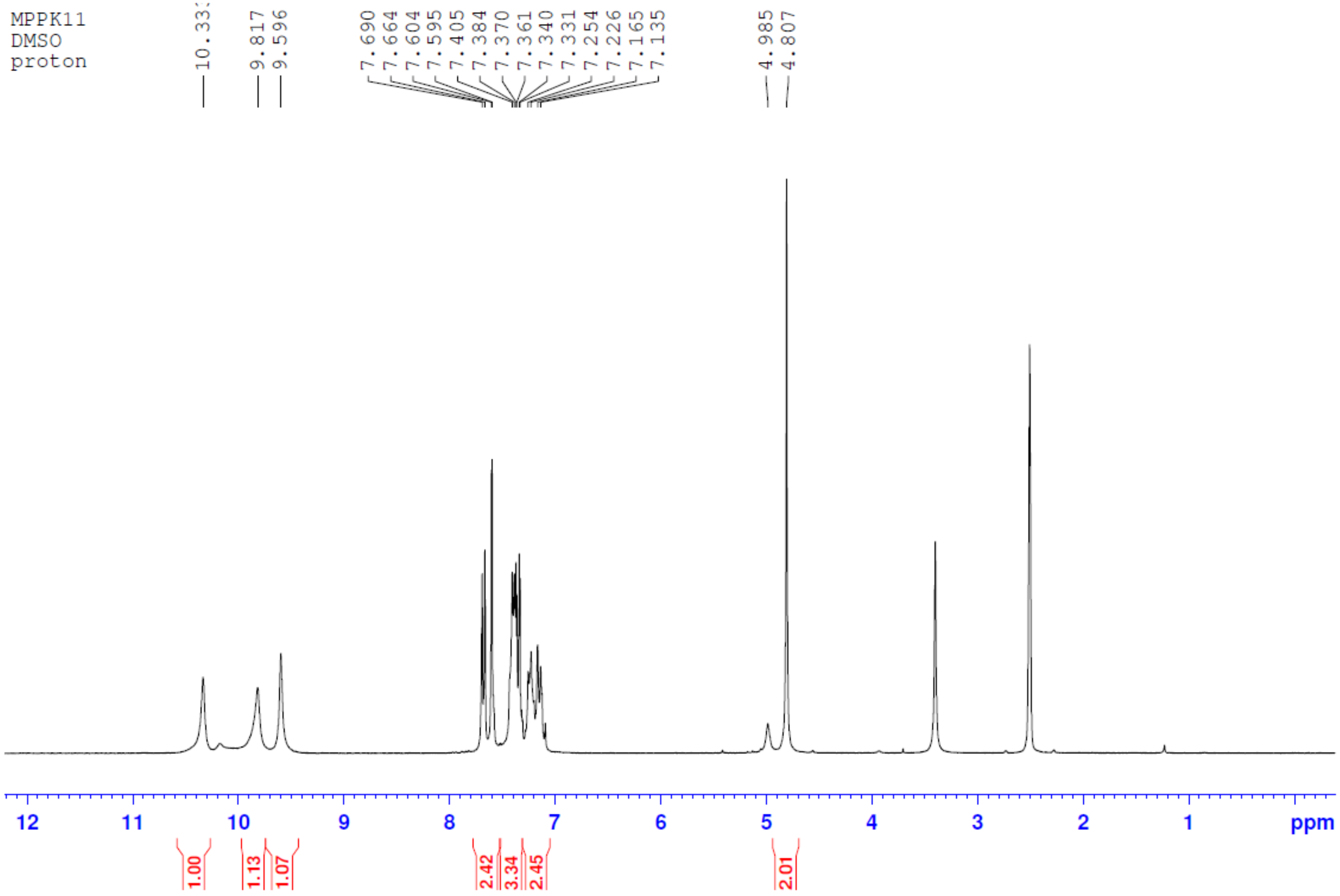

^{1}H NMR for compound PK12

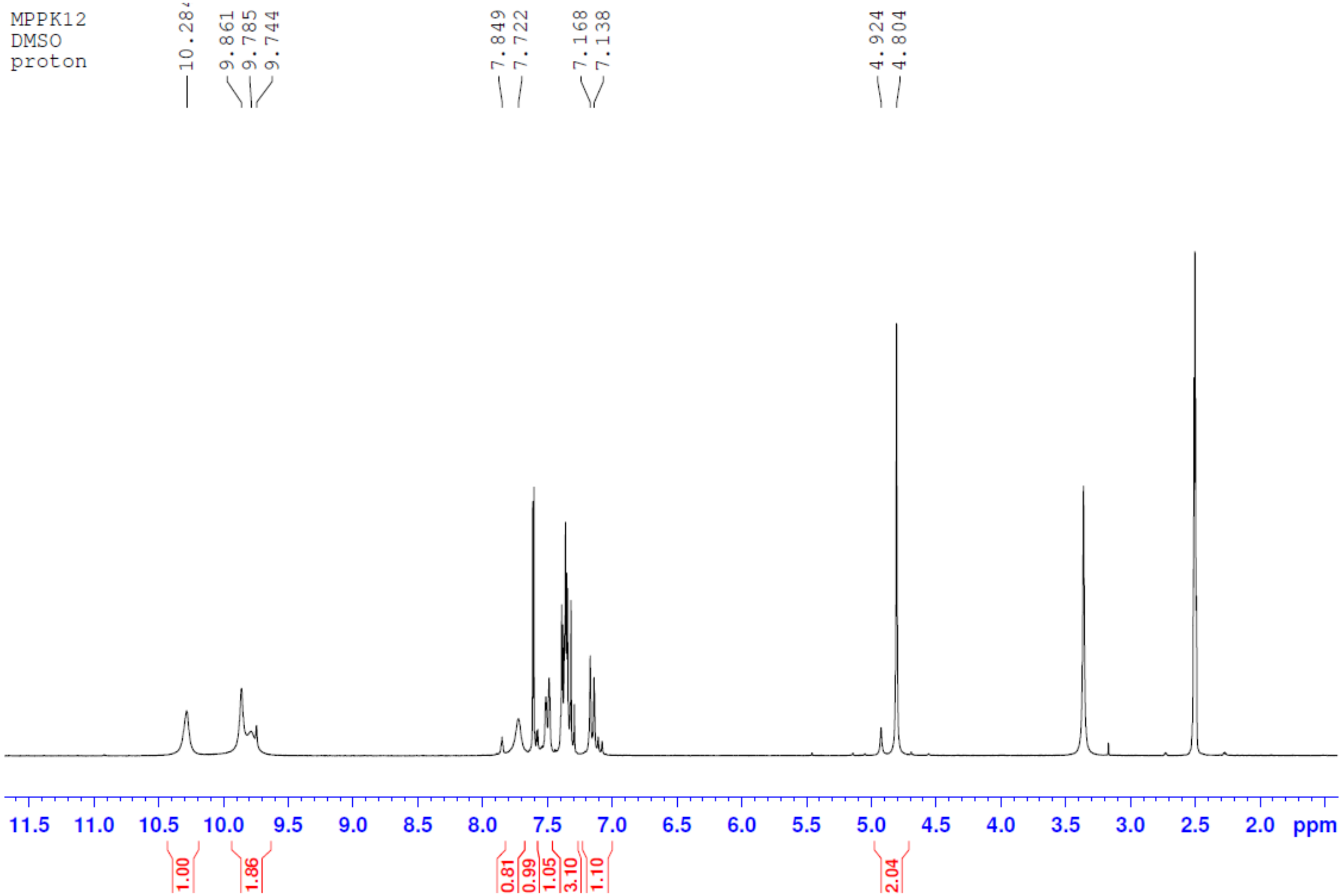

MS spectra

PK1

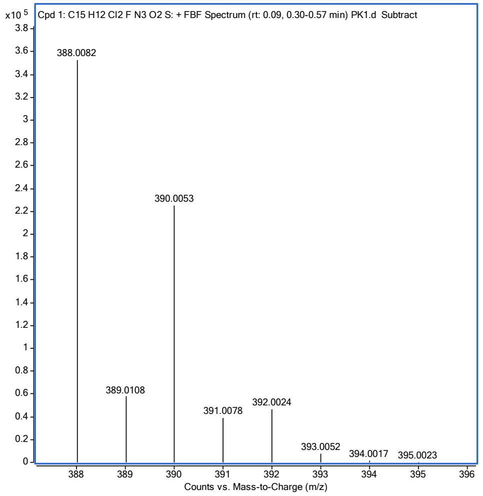

PK2

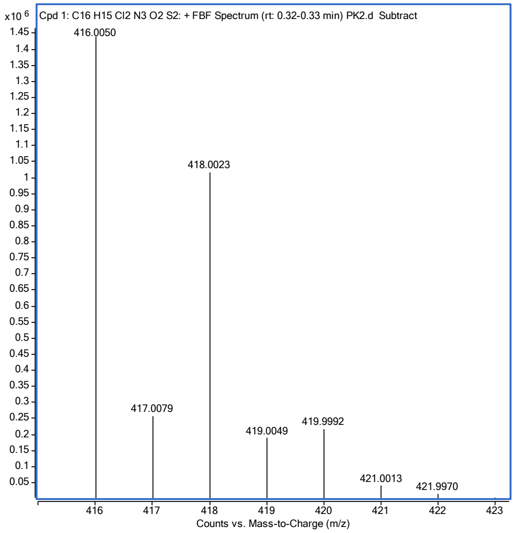

PK3

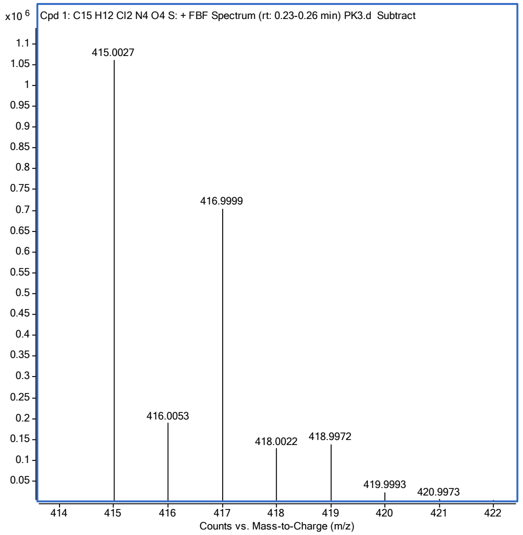

PK4

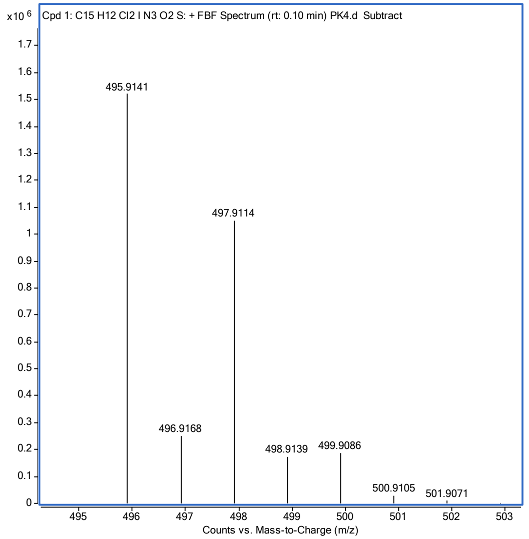

Cpd 1: C15 H12 Cl2 I N3 O2 S: + FBF Spectrum (rt: 0.10 min) PK4.d Subtract
x10 6
495.9141
497.9114
496.9168
498.9139
499.9086
500.9105
501.9071
Counts vs. Mass-to-Charge (m/z)

PK7

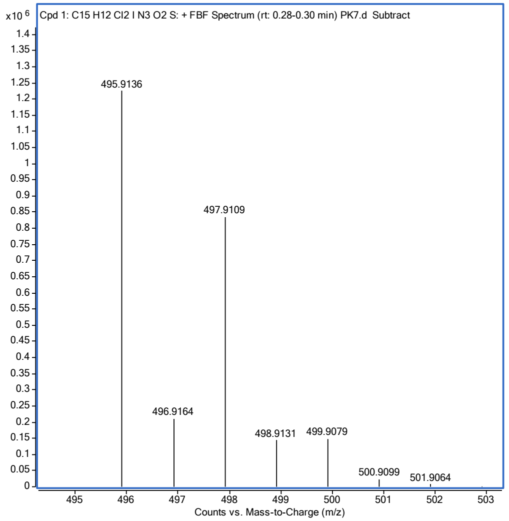

Cpd 1: C15 H12 Cl2 I N3 O2 S: + FBF Spectrum (rt: 0.28-0.30 min) PK7.d Subtract
x10 6
495.9136
497.9109
496.9164
498.9131
499.9079
500.9099
501.9064
Counts vs. Mass-to-Charge (m/z)

PK8

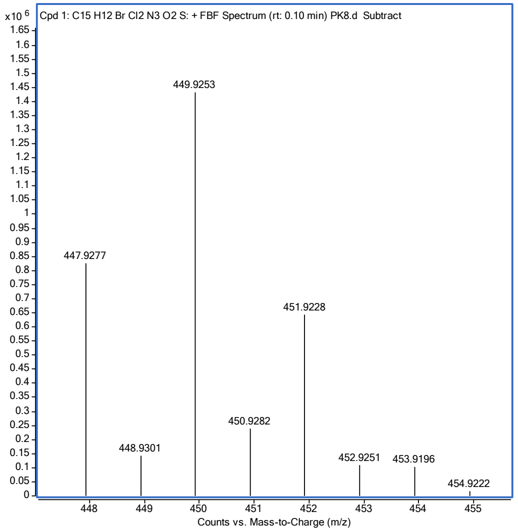

Cpd 1: C15 H12 Br Cl2 N3 O2 S: + FBF Spectrum (rt: 0.10 min) PK8.d Subtract
x10 6
449.9253
447.9277
451.9228
450.9282
448.9301
452.9251
453.9196
454.9222
Counts vs. Mass-to-Charge (m/z)

PK10

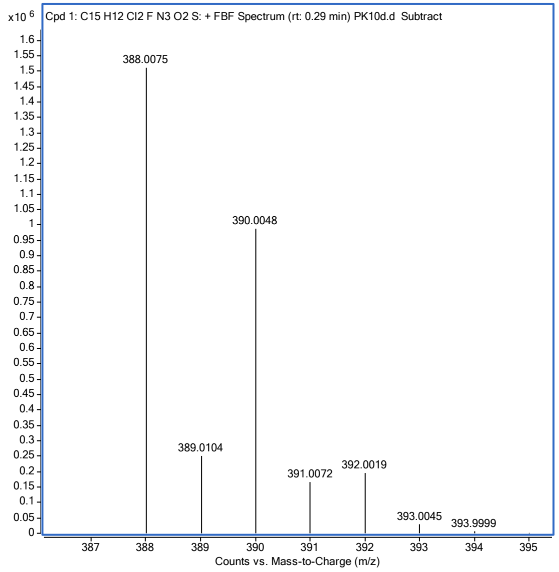

PK11

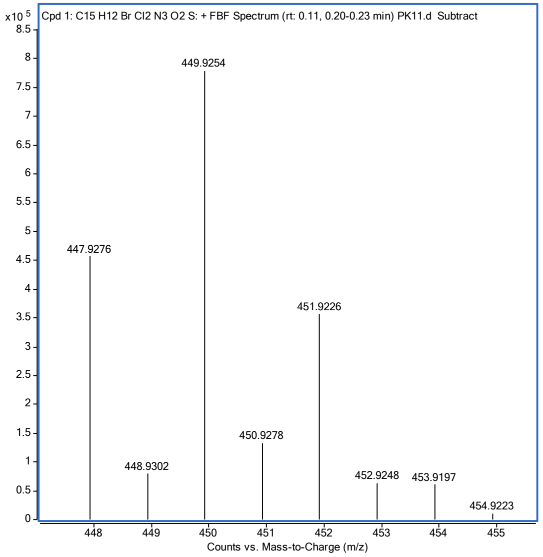

PK12

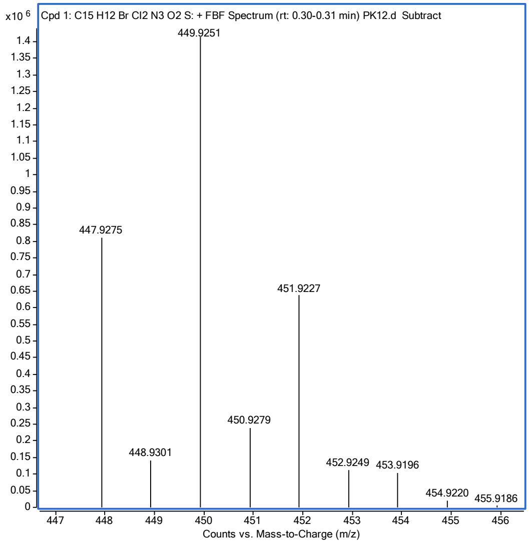

Supplement: Supplementary file 1 [file biomolecules-12-00151-s001.zip › biomolecules-1534197-supplementary.pdf]
